# Ammonia at the net-zero crossroads

**DOI:** 10.1038/s41467-026-75880-2

**Published:** 2026-07-21

**Authors:** Thorin Daniel, Qiong Cai, Lei Xing, Huizhi Wang, Xinli Xu, Lirong Liu, Jin Xuan

**Affiliations:** 1 Faculty of Engineering and Physical Sciences, University of Surrey, Guildford, United Kingdom; 2 Department of Mechanical Engineering, Imperial College London, London, United Kingdom

**Keywords:** Chemical engineering, Environmental impact

## Abstract

Ammonia is indispensable for food production and an emerging carbon-free energy vector, yet its synthesis via the fossil-fuelled Haber–Bosch process makes it a major source of carbon dioxide. We introduce the Ammonia Rainbow, a classification framework combining physics-based modelling, learning-curve analysis, and life-cycle sustainability assessment to evaluate net-zero transitions across eight ammonia production technologies. Here we show that, assuming global ammonia demand doubles to meet projected food and energy needs, net-zero ammonia is not achievable by 2050 under any current policy or technological scenario. Even optimistic deployment of green routes demands extensive infrastructure, consuming over 40 percent of available green hydrogen alongside large fractions of carbon capture and renewable energy capacity. Electrochemical routes offer long-term promise but are unlikely to reach commercial viability before 2070 without disruptive innovation. By exposing these hidden systems-level burdens, we challenge the prevailing assumption of unchecked demand growth and argue for demand-side measures, efficiency improvements, and alternative decarbonisation strategies.

## Abstract (web summary)

This study shows that even under optimistic green technology rollout, net-zero ammonia production remains unachievable by 2050, revealing hidden infrastructure demands that challenge assumptions of unchecked demand growth.

## Introduction

Ammonia is a cornerstone of modern civilisation, supporting food production for nearly half of the global population^1^. Yet its manufacture accounts for over 1% of global carbon emissions, presenting a critical sustainability paradox^2^. In recent years, ammonia has also gained traction as a potential carbon-free energy carrier, projected to play a key role in decarbonising sectors such as shipping and heavy industry^3^. This puts us at a crucial crossroads where decisions on which ammonia production route to follow are being taken.

These dual roles have led to widespread projections of rapidly rising ammonia demand doubling or more by 2050^4–7^. However, these projections may rest on optimistic or insufficiently scrutinised assumptions, both about future demand growth and about the pace and feasibility of technological transitions. Ammonia is currently produced almost exclusively through the Haber–Bosch process, which emits ~2.5 tonnes of CO_2_ per tonne of ammonia^8^. Alternative production routes are emerging, including low-carbon Haber-Bosch variants, electrochemical nitrogen reduction, and decentralised methods using water and air^9–11^. These technologies differ widely in energy sources, feedstocks, maturity, and scalability. Yet existing technological analyses remain fragmented and static, often failing to account for full life-cycle emissions, infrastructure constraints, and the timeline for technological deployment^12,13^. For example, projections assume rapid scale-up of green ammonia without collectively considering the massive requirements for renewable electricity and green hydrogen, or the socioeconomic risks of competing with food systems for ammonia supply^14–16^.

To address these gaps, we introduce the Ammonia Rainbow, a physics-based classification and predictive framework for assessing the future sustainability of eight ammonia production pathways that represent the full range of technological options^3,17–23^. The framework combines process-level modelling with learning curve dynamics, full life-cycle sustainability assessment, and AI-powered social impact analysis. This enables a systematic comparison of both mature and emerging technologies under consistent assumptions, including future cost and emissions trajectories, infrastructure dependencies, and social trade-offs.

Our results show that net-zero ammonia is not achievable by 2050 under any existing policy or technology pathway, assuming global demand doubles. While green and blue ammonia combined can reduce direct emissions by up to 95%, they still face significant lifecycle limitations and impose unexpectedly high demands on various energy infrastructures. Meanwhile, emerging routes such as direct electrochemical nitrogen reduction remain unlikely to contribute meaningfully before 2070 unless breakthrough disruptive innovations occur.

Here, we use the Ammonia Rainbow to evaluate the technological, economic, and environmental viability of these eight ammonia production pathways under realistic demand growth scenarios. We assess whether net-zero ammonia production is achievable by 2050, and quantify the infrastructure, resource, and timeline implications of each pathway. In doing so, we highlight the need not only to accelerate technology development but also to re-evaluate the assumption of unconstrained ammonia demand growth, and demonstrate how predictive, dynamic classification frameworks can support evidence-based decisions in fast-evolving clean technology sectors.

## Results

### The ammonia rainbow

**Fig. 1:**
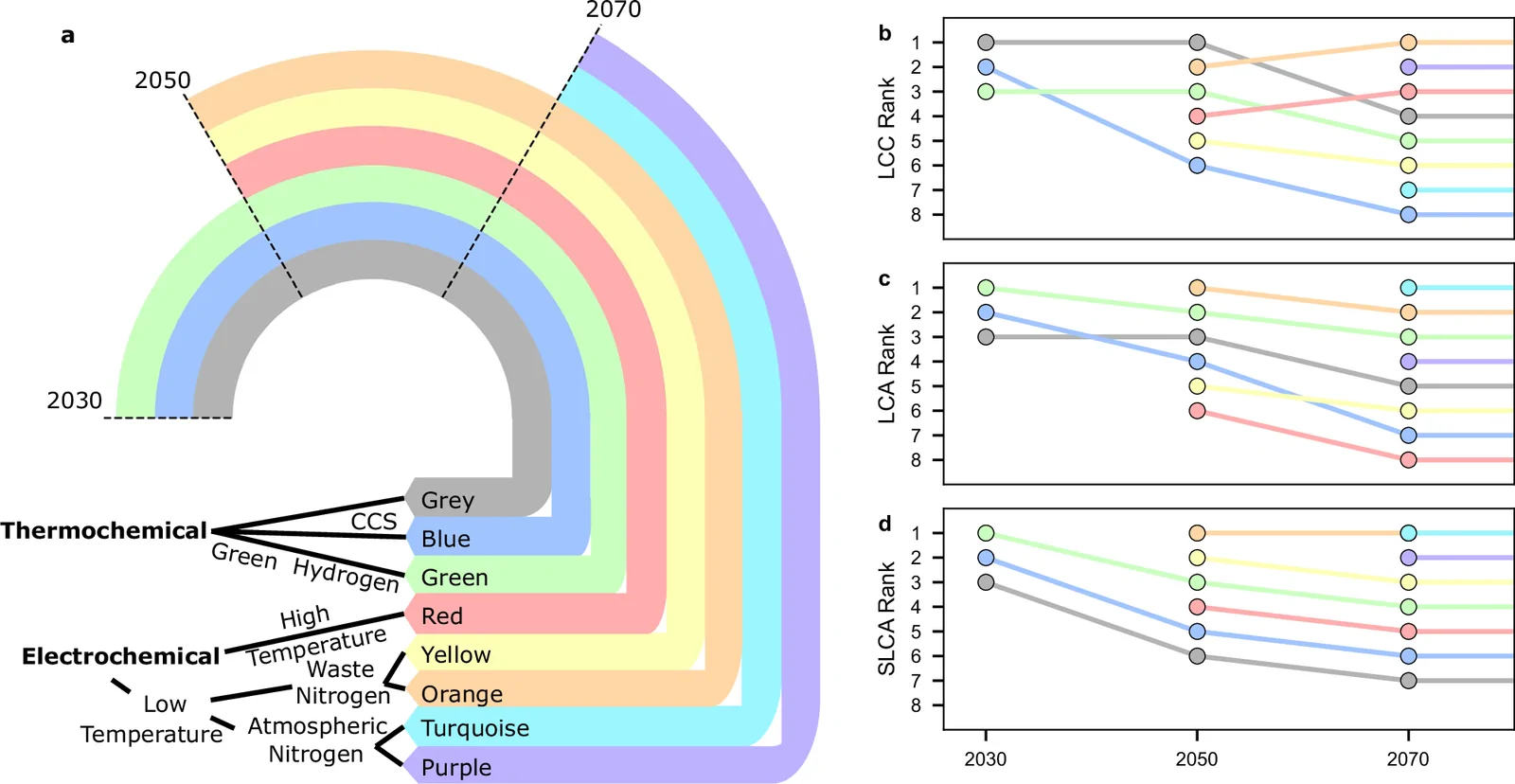
Overview of ammonia production pathways and sustainability rankings. **a** Illustrates the ammonia rainbow; **b**–**d** show technology rankings from 2030 to 2070 based on life cycle assessment (LCA) of allocated emissions, life cycle cost (LCC), and social LCA (SLCA), respectively. In Fig. 1d, orange and turquoise ammonia are ranked equal first for 2070. An additional span is given for 2070 for ease of illustration of these rankings and does not represent a further prediction. CCS is Carbon Capture and Storage.

The Ammonia Rainbow framework (Fig. 1a) offers a systematic evaluation of the rapidly evolving ammonia production landscape. It builds on the ‘Hydrogen Rainbow’ concept but extends it to specifically capture ammonia’s unique inputs: the sources of nitrogen, protons, and energy, with further information available in Supplementary Table 1^24^. Unlike previous static classifications, our framework is predictive and comparative, integrating process-level modelling, probabilistic technology maturation, and life-cycle sustainability metrics to assess both mature and emerging routes on a common basis. The framework encompasses eight representative production pathways, spanning incumbent, low-carbon, and next-generation electrochemical options with detailed definitions shown in Table 1.

**Table 1:** Ammonia Rainbow Colour Description

Grey ammonia	Conventional Haber–Bosch using fossil-derived hydrogen and grid energy
Blue ammonia	Grey route with carbon capture and storage (CCS), offering partial emissions mitigation
Green ammonia	Haber–Bosch fed with green hydrogen from low-temperature water electrolysis
Red ammonia	High-temperature electrolysis of steam and nitrogen, coupled with nuclear energy
Yellow ammonia	Low-temperature electrochemical production from air pollutants (NOx) using renewable electricity
Orange ammonia	Low-temperature electrochemical production from nitrate-rich water using renewable electricity
Turquoise ammonia	Two-step electrochemical synthesis using green hydrogen as an intermediate
Purple ammonia	The ultimate single-step electrochemical nitrogen reduction from water and air

Using a probabilistic learning model underpinned by process-level simulations, we project the technological development of each pathway from 2025 to 2070 based on the progression of their underlying performance metrics, such as efficiency and material usage, over time. This predictive capability allows us to identify the time windows in which each route may become viable. Our results show that by 2030, only grey, blue, and green ammonia are expected to be widely deployable. By 2050, red, yellow, and orange routes begin to emerge. Turquoise and purple ammonia are unlikely to achieve commercial maturity before 2070.

At each milestone (2030, 2050, 2070), we perform life cycle costing (LCC), life-cycle assessment (LCA), and social life-cycle assessments (SLCA), ensuring fair comparison across radically different pathways. The resultant rankings (Fig. 1b–d) reveal how sustainability leadership shifts over time. Underpinning LCC results are in Supplementary Discussion–Fundamental Life Cycle Costing Results, LCA (including midpoint indicators, available in Supplementary Discussion–Fundamental Life Cycle Assessment Results), and SLCA results can be found in sections Supplementary Discussion–Fundamental Social Life Cycle Assessment Results. Economic results are given in US dollars ($).

We find that green ammonia performs well in all categories initially but drops off as new technologies develop. Blue ammonia provides a transitional option with middling performance across all categories. Red ammonia has good cost performance but is poor in other categories mainly due to its association with nuclear energy. Orange ammonia performs well over time, but yellow is poor in both cost performance and environmental impact due to its higher energy and material requirements. Purple and turquoise ammonias have middling costs and good social performance, and Turquoise has good environmental performance, whereas purple, due to its high energy requirements, performs poorly.

By combining dynamic technology forecasting with integrated sustainability assessment, the Ammonia Rainbow framework provides a clear, predictive roadmap for comparing diverse ammonia pathways. In the following sections, we use the framework to show how the diverse ammonia production pathways contribute to the global net-zero transition.

**Fig. 2:**
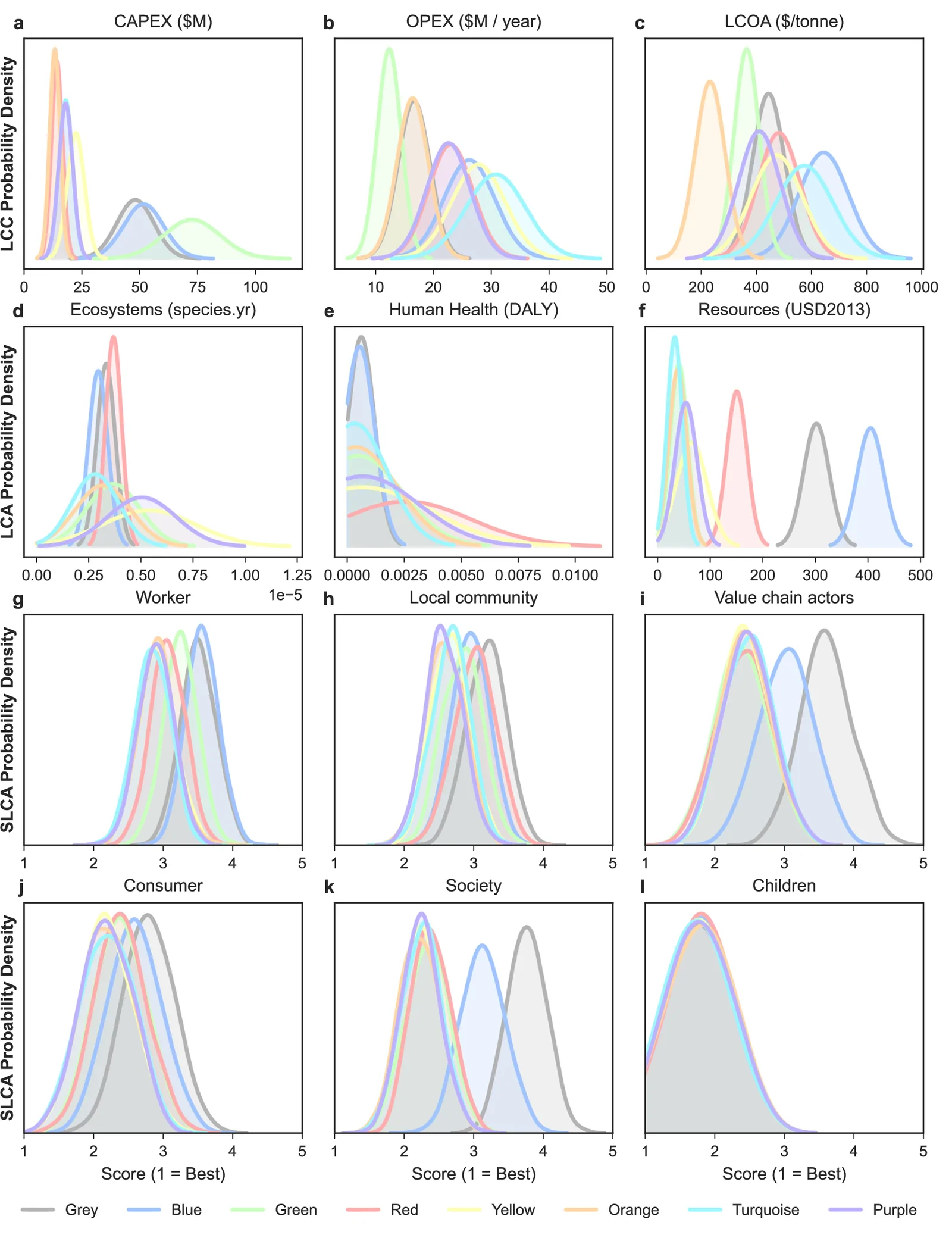
Probability density curves for technology performance in Life Cycle Costing (LCC) (a-c), Life Cycle Assessment (LCA) (d-f), and Social LCA (SLCA) (g-l) categories, for 2070. LCC probabilities are for the variance in the CAPEX and OPEX, which vary by 50%, and the subsequent effect on LCOA. LCA probabilities are the uncertainty in endpoint indicators. SLCA uncertainties are the distribution of score each technology receives within an SLCA category from the subcategory scores, categories and subcategories are shown and defined in Supplementary Table 6.

Figure 2 presents the probabilistic Life Cycle Sustainability Assessment (LCSA) results that underpin the technology rankings in Fig. 1b–d and their associated rank uncertainties in Supplementary Fig. 21. For LCC (Fig. 2a–c), independent variations of up to ±50% in capital expenditure (CAPEX) and operating expenditure (OPEX) are propagated into the levelised cost distributions. Lower-cost options (orange, purple) exhibit narrower absolute spreads, but the resulting levelised costs still overlap substantially across technologies. In 2070, the central 50% credible intervals for several pairs (grey–green, yellow–turquoise, orange–purple) intersect, explaining the rank crossovers in Supplementary Fig. 21a.

Uncertainty across midpoint indicators, which feed into the endpoint indicators of the LCA, comes from the ecoinvent database, where the individual measurements for each component of each technology have a variance which ultimately contributes to the probability distributions shown in Fig. 2d–f. Grey and blue ammonia have very distinct, narrow distributions, particularly for the resources indicator. This results in the region around ranks 5 & 6 in Supplementary Fig. 21b being highly concentrated, with the other technologies being shifted away either higher or lower. This shows that while certain groupings of technologies may have higher uncertainty in the ranking, it will likely be with each other in small changes rather than large overall shifts.

Figure 2g–l illustrates the uncertainty in the SLCA. In many cases, the social aspects of the technologies are not strongly differentiated. Therefore, the overall ranking rests on a small number of radically different subcategories with strong differentiation between technologies, such as Society and Value Chains. Even in these subcategories, only blue and grey show a clear difference from other technologies. As a result, blue and grey are very likely to occupy ranks 7 and 8, respectively, whereas other technologies have a shared probability of occupying the top quartile of the SLCA ranking (Supplementary Fig. 21c).

In addition to the rank uncertainty shown in Fig. 2, the learning curves that model the development of these technologies over time contribute another source of uncertainty. To account for this, we varied both the development rates (*k* values) and the type of learning curve employed, as shown in Supplementary Table 2 and Supplementary Fig. 19. Perturbations in the *k* value cause variations through the middle phase of the curve’s development but ultimately do not significantly alter the overall trend. A similar observation applies to the comparison between the sigmoidal and Gompertz logistic curves; whilst they vary at specific timepoints (particularly around 2050), they generally show a similar upwards development trend. Crucially, regardless of these temporal perturbations, the rankings and their overarching narrative remain robust.

### Net zero transition pathways

Here we show different net zero transition scenarios for the three largest ammonia producing locations: the People’s Republic of China (PRC), the United States of America (USA), and the European Union (EU), between 2025 and 2050 (2060 for PRC due to policy time horizons), noting from aforementioned analysis, only green and blue ammonia will be meaningful options for the mid-century timeframe, due to the long development time of other technologies. We analyse direct and indirect emissions, exposing the gulf between the two for green, blue and grey ammonia. From the life cycle assessment, we calculate grey, blue, and green ammonia to have total emissions of 3.94, 2.59, and 0.63 kg_CO2e_ kg_NH3_^−1^, which are significantly above the direct CO_2_ emissions of 2.5, 0.25, and 0 kg_CO2e_ kg_NH3_^−1^ widely reported for each technology^8,17,25^. Emissions are given in units of mass of CO_2_ equivalent, i.e., global warming potential (GWP), per unit mass of ammonia produced.

We recognise that regional policy strategies strongly influence the pace and composition of the transition. For example, in the USA, historical policy reliance on Carbon Capture and Storage (CCS) strongly favours blue ammonia adoption and hence only blue ammonia is considered^26,27^. The EU policy has a strong focus on green hydrogen to drive the economy, making green ammonia likely a preferred route^28,29^. Here, we assume a 3:1 green/blue mixed ratio for new technology deployments during the transition. In contrast, the PRC, as the world’s largest ammonia producer, adopts a more incremental approach focused on efficiency improvements and gradual CCS deployment^30,31^. Therefore, a lower green/blue ratio of 2:3 is assumed. For each region, we examined fast, medium, and slow roll-out scenarios which are derived from varying technology development rates, to capture the impact of both policy support and technological progress. Further details of the scenario rollouts are summarised in supplementary Methods 9: Net Zero Pathways.

**Fig. 3:**
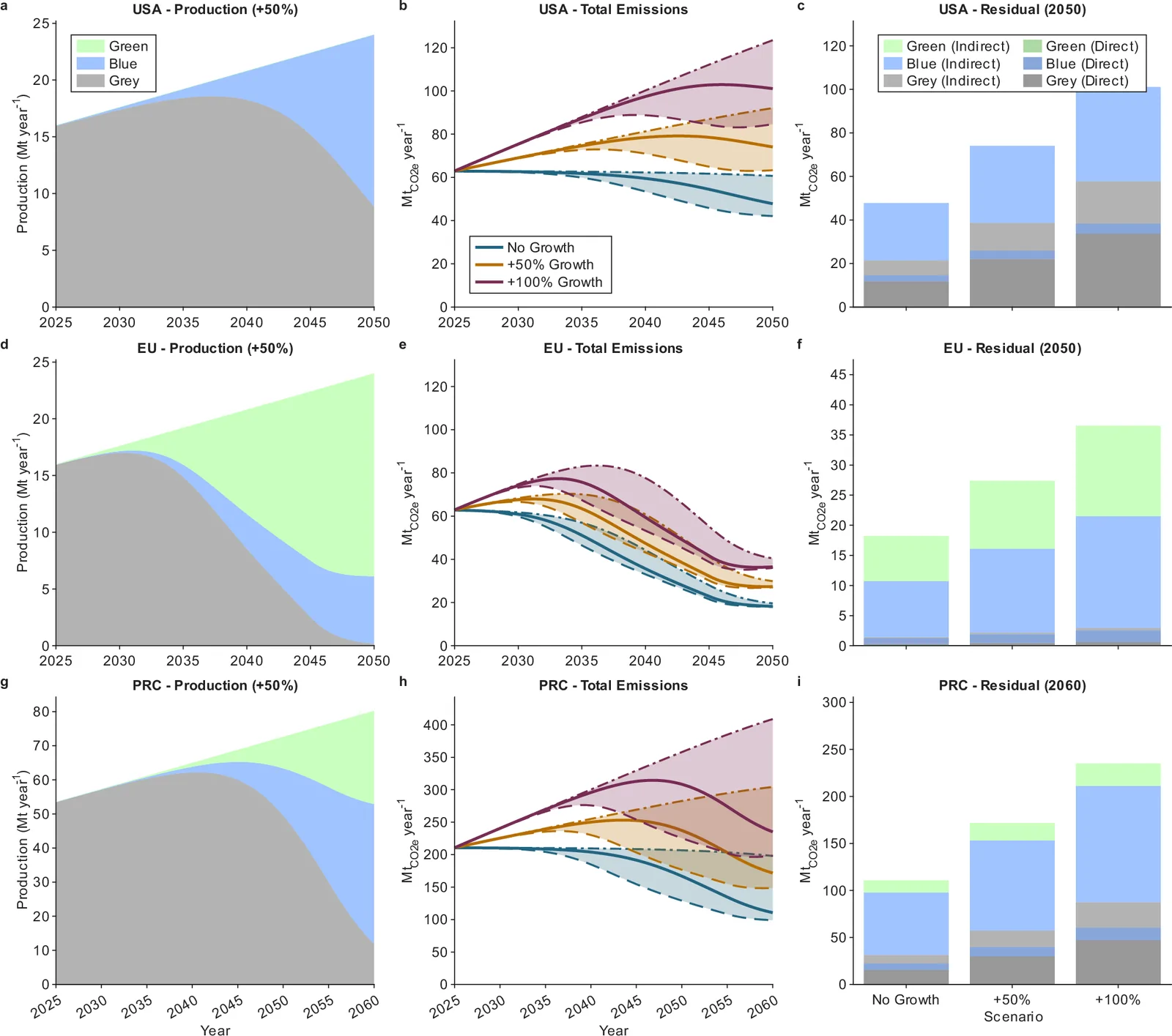
Life cycle emissions, and residual emissions of ammonia production technologies under 2050/60 demand and growth scenarios. Emissions for the USA (**a**–**c**), EU (**d**–**f**), and PRC (**g**–**i**) are shown directly under a 50% increase assumption (**a**, **d**, **g**). Panels **a**, **d**, **g** show the deployment of technology and the relative production of each ammonia technology under a 50% growth scenario–with 0% and 100% growth shown in Supplementary Fig. 20. Panels **b**, **e**, **h** show the life cycle emissions under 0%, 50%, and 100% growth scenarios, with 5^th^, median, and 95^th^ percentile lines representing fast, average, and slow deployment speeds. In **c**, **f**, **i** the residual emissions in 2050 for each growth scenario are broken down into the contributing technology, from both from indirect life-cycle emissions and direct emissions. PRC results include 2060 values where applicable, due to PRC policy targets in 2060 rather than 2050.

Recognising the uncertainty in ammonia’s dual role as a vital fertiliser and an emerging energy carrier, our analysis explores three demand trajectories: a doubling of global ammonia demand (100% growth) by 2050 to meet projected growth in food and energy applications, as projected by various studies and reports^4,6^; a moderate 50% growth reflecting partial energy adoption and efficiency improvements; and a flat demand (0% growth) scenario representing strong demand-side interventions and circular nitrogen management. Combining the above growth rate (0%, 50%, 100%) with the policy scenarios (USA, EU, PRC) results in various patterns of grey/blue/green mix evolving over time. They are visualised in Fig. 3 (Panels a, d, g) for the 50% growth, and Supplementary Fig. 20 for 0% and 100% growth. The growth rate also affects the final mix of ammonia in 2050/60, for example under the 0% growth scenario, the USA achieves greater than 70% blue ammonia penetration, while under the 100% growth scenario this is less than 60%.

As depicted in Fig. 3, our analysis indicates that a twofold increase in global demand renders the 2050 net-zero target unattainable, regardless of the existing policy and technology mix. Even the most aggressive decarbonisation pathway, dominated by green ammonia (e.g., the EU case) retains at least 29.5% residual life-cycle emissions, leaving a clear gap to net-zero. By comparing the EU and PRC subfigures we can see that the green-to-blue deployment ratio emerges as the most critical factor in determining decarbonisation potential, as blue ammonia, despite low direct emissions, maintains significant life-cycle emissions from increased energy usage from upstream hydrogen production and CO₂ capture. For deployment scenarios with high green ammonia ratio, as seen in Fig. 3b, e, h technology deployment rates have only a modest influence on overall decarbonisation, highlighting that scaling the right technology mix matters more than marginal efficiency gains. On the other hand for blue ammonia dominated cases, the deployment rate is crucial in overall decarbonisation, potentially the difference between several hundreds of millions of tonnes of CO_2_ equivalent reductions.

Moderating ammonia demand (1.5x growth scenario, Fig. 3b, e, h substantially narrows the net-zero gap, but residual life-cycle emissions still prevent full net-zero alignment. The flat demand, i.e., no growth scenario, enables the possibility of most closely approaching net-zero direct emissions by 2050, showing that green ammonia needs to dominate to approach net zero under any scenario. This underscores the pivotal role of demand-side management. Improving fertiliser use efficiency, implementing nitrogen recycling strategies, prioritising high-value energy applications for ammonia, and restraining non-essential expansion for energy storage are all critical for aligning ammonia’s growth with climate goals.

As Fig. 3c, f, i illustrates, under the 100% increase scenario, the USA’s residual emissions are driven primarily by direct grey ( ~ 34%) and indirect blue ( ~ 43%) ammonia production. While transitioning away from grey ammonia significantly reduces direct emissions, relying heavily on blue ammonia introduces substantial hidden upstream costs. Conversely, although the EU scenario projects similar absolute emissions to the USA, its footprint is overwhelmingly dominated ( > 93%) by indirect life-cycle emissions from blue and green technologies. Similarly, the PRC demonstrates a much lower direct emissions share ( ~ 27%) but generates a vastly larger overall footprint of nearly 240 Mt_CO₂eq_. Ultimately, as regions adopt alternative ammonia technologies, the emissions burden shifts from point-source manufacturing to the upstream supply chain, making supply chain efficiency the primary bottleneck for true decarbonisation.

### System constraints

The net zero transition of ammonia production imposes substantial, often hidden, burdens on supporting energy and carbon management systems. Our analysis extends beyond emissions to evaluate the feasibility of large-scale rollout under current and projected infrastructure for green hydrogen, CCS, and renewable electricity, providing a systems-level perspective on the practical limits of low-carbon ammonia deployment.

**Fig. 4:**
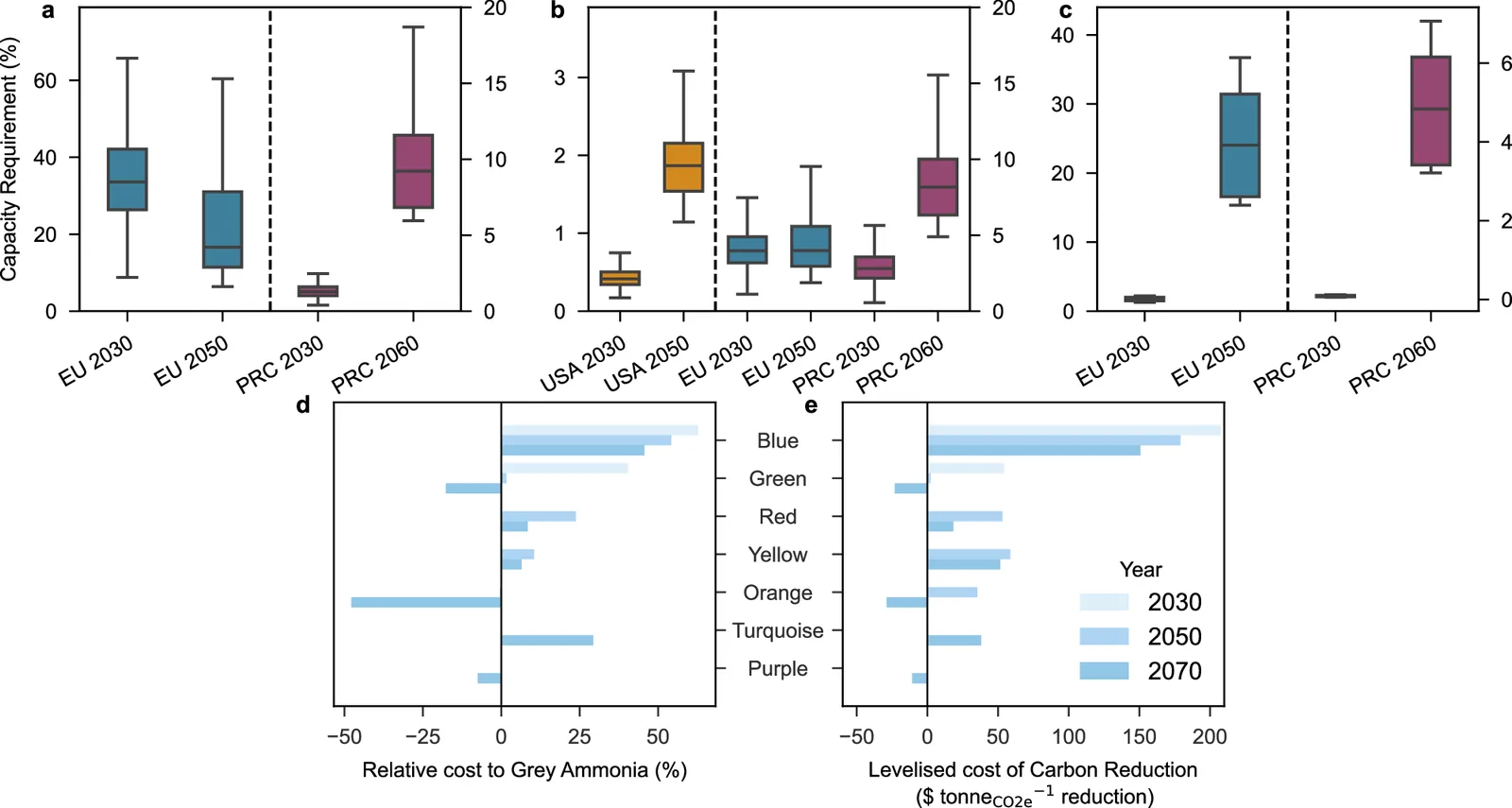
Regional capacity requirements and economic performance of ammonia production pathways. Panels show 2030 and 2050/60 requirements of assumed available capacity for green hydrogen (**a**), Carbon Capture and Storage (CCS) (**b**), and renewable electricity (**c**) in the EU, USA, and PRC. Box-and-whisker plots show the distribution of capacity requirement across 30,000 combinations of three demand scenarios (no change, +50%, +100% increase, as in Fig. 3) and 10,000 Monte Carlo simulations of technology deployment rate, whiskers follow the 1.5×IQR convention; points beyond this range are omitted and full data is available in Supplementary Data 4–Source Data Fig. 4. USA has no values in panels a and c as there is no green hydrogen for the USA scenario. Panel d shows the levelised cost of ammonia (LCOA) relative to grey ammonia; panel e presents the levelised cost of Global Warming Potential (GWP - CO_2eq_) abatement in 2030, 2050, and 2070 relative to grey ammonia. Both d & e represent location-insensitive trends.

For all the net zero transition pathways of ammonia production shown in Fig. 3, large amounts of CCS, green hydrogen, and renewable energy capacity would be required. Without adequate supply of these utilities, the rollout of low carbon ammonia risks being bottlenecked. We create three scenarios, using 2030 and 2050 targets for carbon capture, green hydrogen, and renewable energy capacity (Supplementary Table 8), to encompass the spectrum of possible combinations of capacity and requirement, the results of which are shown in Fig. 4. The upper bounds of Fig. 4a–c whisker plots represent the maximum requirement for a utility, and minimum deployment, the lower represents the inverse, and the median line represents the median capacity and requirement–which we term the moderate transition scenario.

Our results show that, even under moderate transition scenarios, ammonia production rapidly becomes a major consumer of CCS and green hydrogen capacity. As shown in Fig. 4a, by 2030 ammonia production could require as much as 65% of green hydrogen production available in the EU and more than the 60% by 2050 if green ammonia deployment rate significantly outstripped green hydrogen deployment. Alternative ammonia deployment could consume 5 and 7% of the PRC and EU’s planned 2030 CCS capacity, as shown in Fig. 4b. Yet, as Fig. 4b also shows, by 2050 ammonia’s share of CCS capacity grows sharply in all regions, rising as high as 15%, implying that dedicated CCS infrastructure would be required to prevent ammonia decarbonisation from competing with other sectors for limited storage capacity. Initially in 2030, the renewable energy requirement of ammonia isn’t significant, but as demonstrated by Fig. 4c, by 2050 the total amount of renewable energy required for low carbon ammonia production in the EU and PRC may be as much as 35% and 7.5% of total capacity required, respectively.

Affordability adds another critical dimension. As Fig. 4d shows, blue ammonia remains substantially more expensive than grey ammonia through 2030, falling gradually over time and never achieving cost parity without carbon pricing^32^. Green ammonia is initially nearly 40% more expensive than grey, though its cost declines sharply by 2050 as electrolyser and renewable prices fall. This reflects the assumed cost reduction trajectory of green hydrogen. Red, yellow, and orange ammonia remain more costly through mid-century, and by 2070 yellow and red ammonia remain above grey due to their higher energy and capital intensity. By this time, turquoise and purple ammonia become viable, with purple potentially cost competitive.

Cost per tonne of CO₂-equivalent reduction is a widely used indicator to assess how expensive it is to decarbonise a given sector^33^. Figure 4e shows that blue ammonia performs poorly on this metric: in 2030 it is roughly 25% more expensive than green ammonia but delivers only 34% life-cycle mitigation, translating to an abatement cost nearly seven times higher per tonne of CO₂-equivalent reduced. When benchmarked against values reported for other sectors, in US dollars, such as power generation (<$100 tonne_CO2_^−1^)^34,35^, Iron and steel production (<$100 tonne_CO2_^−1^)^36^, and cement production (<$150 tonne_CO2_^−1^)^37^, our estimate for blue ammonia emerges as a relatively costly option, particularly in the near term, although this cost is for life cycle emissions. This high marginal cost of abatement may lead to de-prioritisation of ammonia within broader net-zero planning, especially if other sectors can achieve larger emission reductions at lower cost. Nevertheless, by 2070, several low-carbon ammonia routes achieve negative carbon costs, meaning their levelised production cost falls below that of grey ammonia while delivering substantial GWP reductions, offering a net economic benefit once technologies mature. Overall, from a cost perspective, green ammonia offers the most realistic route for ammonia decarbonisation, particularly in the near term, this route is highly dependent on the price of green hydrogen and is subject to renewable energy and green hydrogen availability restrictions.

We further calculated the overall additional cost for net-zero transition of ammonia production under the scenarios analysed earlier, which implies a significant macroeconomic burden, with the additional cost of decarbonised ammonia production reaching $30 bn by 2050 in the high-demand scenario for the PRC. This indicates that substantial additional investment would be required to align ammonia’s decarbonisation with broader climate goals without compromising fertiliser affordability or industrial competitiveness.

### Progressive steps or disruptive leaps for ammonia beyond 2050

The decades after 2050 will test whether ammonia’s transition is achieved through incremental substitution or disruptive technological leaps. In this section, we focus on purple ammonia, direct nitrogen reduction from air and water, and assess its potential through a combination of techno-economic analysis, environmental life cycle assessment, and social life cycle assessment. By systematically modelling how shifts in key technological parameters such as current density, faradaic efficiency, electricity prices, and material lifetimes affect production costs, emissions profiles, and social outcomes, we explored both incremental and disruptive innovation pathways. This integrated assessment highlights not just the scale of improvement required, but also where transformative breakthroughs would matter most.

**Fig. 5:**
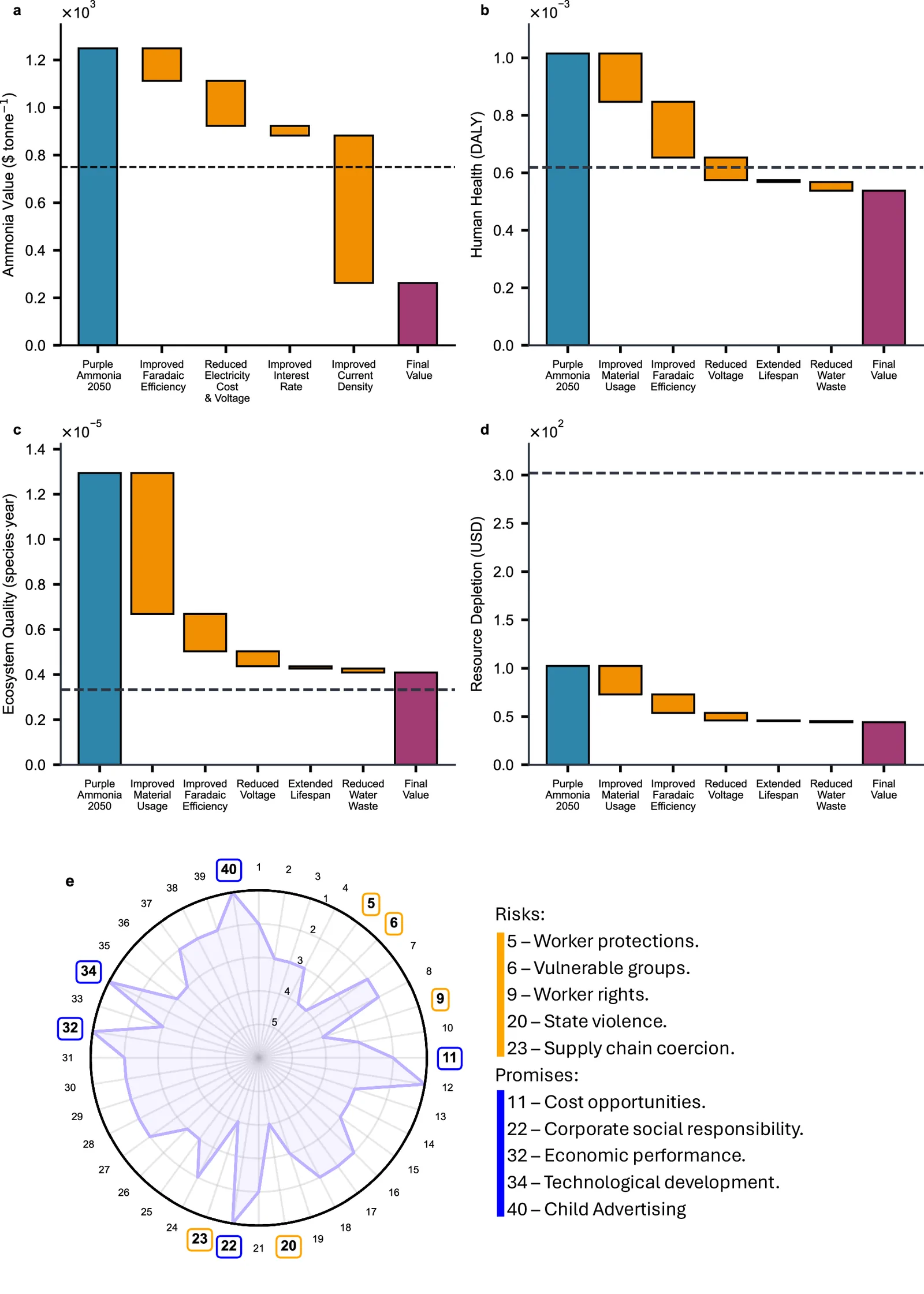
Full breakdown of Economic, Environmental, and Social dimensions for purple ammonia with disruptive pathways for development. **a** Waterfall diagram of purple ammonia production cost pathways, beginning from a hypothetical 2050 baseline cost. Life cycle assessment (LCA) results for (**b**) Human Health (**c**) Ecosystems (**d**) Resources. **e** Radar diagram of social LCA categories for purple ammonia and full list of subcategories. **a**–**d** show a dashed horizontal line where the grey ammonia value is for comparison.

As shown in Fig. 5a, economically, purple is constrained by both its electrochemical performance and its dependence on cheap renewable electricity. Our analysis shows that only disruptive improvements, such as current densities surpassing 1 A cm⁻² while electricity price and voltage falling towards $10 MWh⁻¹ and 1.3 V, respectively, could bring costs close to parity with grey ammonia. Without these leaps, purple would struggle to compete commercially. Figure 5b–d shows that, environmentally, purple’s early-stage footprint exceeds that of grey ammonia due to the high material and energy demands of overcoming difficult reaction kinetics. Lifecycle impacts on ecosystems are particularly persistent, even as efficiencies improve. Significant reductions in voltage and water waste, coupled with faradaic efficiency approaching 98%, are necessary for purple to surpass grey in human health impacts. Yet even under optimistic assumptions, its ecosystem performance remains challenging, pointing to the need for entirely new catalytic materials and reaction pathways to unlock genuine sustainability gains.

The social LCA results are displayed in Fig. 5e, representing both risk and promise. Our results highlight strong potential for positive contributions for smallholders and farmers, local economic development, and technological innovation. Decentralised, infrastructure-light production could empower farmers and off-grid communities by buffering them from volatile global markets. Innovative green technologies are likely to bring positive economic growth and new job opportunities. However, these opportunities are fragile. Without strong protections for workers and meaningful community involvement, the high upfront costs of new technologies risk amplifying exploitation and inequality. Women are particularly vulnerable workers in emerging green technology markets, and so additional protections may be necessary to ensure gender equality. Thus, disruptive innovation must extend beyond the lab bench, embedding social safeguards into the technology’s development and deployment.

Taken together, purple ammonia illustrates the duality of the Ammonia Rainbow: a technology that could radically decouple production from fossil fuels and centralised infrastructures, but only if disruptive advances in electrochemistry, materials, and governance are realised. Absent such breakthroughs, purple will remain aspirational, arriving too late to shape 2050 net-zero goals and instead confined to a niche role beyond 2070.

## Discussion

Our results call into question the prevailing narrative that net-zero ammonia by 2050 is both achievable and desirable under continued growth in demand. Even under the most optimistic assumptions no pathway delivers full decarbonisation by mid-century when ammonia demand doubles. Green and blue routes, which form the foundation of most current strategies, reduce direct emissions but remain constrained by life-cycle impacts and systemic dependencies; while emerging electrochemical pathways are unlikely to reach commercial maturity before 2070. The expectation that technological substitution alone can reconcile expanding demand with climate targets is therefore fundamentally misplaced.

This tension exposes a deeper issue: the assumption that ammonia demand will continue to rise unabated. Growth is often justified by the dual imperatives of increasing food production and positioning ammonia as a future energy carrier. Yet our analysis shows that such expansion dramatically increases both the cost and difficulty of decarbonisation. On a cost-per-tonne CO₂-equivalent basis, we show ammonia is already among the most expensive sectors to abate, despite being considered an easy area for CCS to be applied, raising the risk that it is deprioritised relative to other, more cost-effective mitigation options, such as steel or power^37^. Additionally, if carbon capture or renewable energy capacity targets are missed, ammonia is likely to be further restricted in transitioning to renewable production causing emissions to remain high.

At the system level, ammonia’s transition is tightly coupled to the availability of low-carbon hydrogen and supporting energy infrastructure. Today, the vast majority of hydrogen production occurs from fossil fuel based sources, with only a small fraction from green or blue sources, with the higher cost of production and limited infrastructure being primary barriers to larger scale deployment. Beyond 2050, if electrochemical synthesis can achieve the disruptive performance we explore, the synthesis technology itself may become a primary determinant of both cost and environmental impact, offering massive improvements.

Green ammonia is the most promising alternative to grey initially, as it offers the lowest cost and most mature technology, even though other technologies are able to outcompete it on cost eventually. Blue ammonia provides an earlier emissions reduction where CCS is available, but remains more expensive than grey and typically more costly per tonne of CO₂ abated than green by mid-century. Emerging electrochemical routes become potentially competitive in the longer term,with lavender and purple ammonia requiring the disruptive performance improvements explored in Fig. 5.

If real world capacity of CCS, renewable energy, or green hydrogen falls short of projections in Fig. 4, the required capacity would increase proportionally, further burdening the ammonia demand. If, for example, green hydrogen capacity reaches only ~50 % of the assumed level, then under the same ammonia technology mix either ammonia would consume an even larger fraction of green hydrogen (crowding out other uses), or green ammonia deployment would be constrained, leading to higher reliance on grey/blue routes and correspondingly higher life cycle emissions. Therefore, the decarbonisation of ammonia is not only a matter of plant-level technology choice but also of system-level prioritisation of where scarce green hydrogen is used. The same logic applies to CCS and renewables: shortfalls in these underpinning systems amplify the difficulty of reaching the emissions trajectories shown in Fig. 3. Ultimately, the primary purpose of these scenarios is not to forecast precise year-by-year trajectories, but rather to demonstrate how different aggregate demand levels, such as no growth, moderated growth, or a doubling of demand, interact with technology choices and system constraints.

To contextualise these findings, it is important to clarify how upstream hydrogen and electricity supply relate to the intrinsic ammonia synthesis technologies. For conventional thermochemical routes (grey and blue), steam methane reforming, process-heat generation, and the Haber–Bosch loop are co-located and tightly integrated, so that hydrogen production and ammonia synthesis form a single coupled unit. Our hotspot analysis shows that most GWP arises from on-site fuel combustion and reforming, but these contributions cannot be cleanly separated from the synthesis stage because they are produced within the same process. By contrast, for fully electrified pathways the synthesis loop is physically and functionally decoupled from the primary energy source, i.e., electricity and green hydrogen are supplied from external systems. Their on-site synthesis stage has typically low or no direct emissions, and its main influence is through efficiency, which determines how much upstream electricity and hydrogen are required per tonne of ammonia. Supplementary Table 19 quantifies this distinction for the electrified routes by reporting the share of GWP attributable to upstream electricity/hydrogen supply versus the on-site synthesis stage. These results indicate that the Ammonia Rainbow framework compares technology–utility systems in which synthesis efficiency governs upstream energy demand. Together, these factors determine each route’s sustainability performance and relative rankings.

A further implication of this systems perspective is that the comparative performance of pathways depends strongly on the assumed electricity and heat sources. In principle, each synthesis technology could be coupled to multiple plausible utility configurations. Exploring such combinations would effectively extend the Ammonia Rainbow with additional colours, each representing a distinct technology-utility system. Substituting alternative but plausible electricity or heat sources, or varying their carbon and cost intensity within realistic ranges, can shift technologies significantly in the LCA or LCC rankings. Therefore, the Ammonia Rainbow results should be interpreted as rankings of technology–utility systems rather than synthesis technologies in isolation, and it should be recognised that both the choice of pairing and the intensity of the underlying utilities condition the precise ordering of pathways, especially among low‑carbon options.

The interpretation of our comparative results also depends on how the ranking framework is constructed. The LCC, LCA and SLCA rankings are sensitive to the implicit weighting of indicators. We adopt equal weighting across all indicators as a transparent baseline, but stakeholders who prioritise, for example, climate mitigation over resource depletion, or affordability over social risk, would obtain different composite scores and a different ordering of technologies. It is therefore important to distinguish between absolute performance changes and relative ranking shifts. For instance, the absolute cost and emissions trajectories for grey and blue ammonia evolve only modestly over time, yet their positions in the strand‑specific rankings decline markedly as new technologies enter the comparison set.

A limitation of this study lies in the representation of technological development through stylised learning dynamics. While the sigmoidal learning formulation captures the combined effects of scale-up, learning-by-doing, and technological maturation, it does not explicitly resolve their individual drivers, nor fully capture structural uncertainties in innovation pathways. Similarly, excluding temporal variability and buffering requirements in energy systems likely underestimates system costs. However, these limitations do not alter the central conclusions in this paper.

Looking beyond 2050, the prospect of disruptive innovation introduces both opportunity and uncertainty^38^. Decentralised, infrastructure-light production could transform supply chains, reduce exposure to volatile markets, and support smallholder resilience. However, the technical barriers remain substantial, and the social and environmental implications are not inherently positive. Without proactive governance, including labour protections and community engagement, these technologies risk reproducing existing inequalities even as they reduce emissions.

The implications for policy are therefore twofold. First, supply-side interventions must be coordinated at the system level, linking ammonia production with hydrogen deployment, carbon management, and energy system planning. Second, and more fundamentally, demand-side measures become indispensable. Improving fertiliser efficiency, implementing circular nitrogen management, and carefully prioritising ammonia’s role as an energy carrier can substantially reduce the scale of the decarbonisation challenge. Indeed, only under moderated or flat demand scenarios does alignment with net-zero trajectories become plausible, highlighting that managing demand is as critical as advancing supply technologies.

These findings suggest that the debate should not be framed solely around how to decarbonise ammonia, but also how much ammonia the world can afford to sustain in a net-zero future. Rebalancing expectations by moderating demand growth, improving fertiliser efficiency, and carefully assessing the role of ammonia as an energy vector may be as important as pursuing new technologies. The ammonia rainbow framework highlights that achieving net-zero is not simply a matter of accelerating innovation, but of confronting trade-offs between demand, resource allocation, and equity. In this context, the critical question is not only how ammonia can be decarbonised, but how much ammonia a net-zero world can sustain.

## Methods

### Technology selection and modelling

Technology routes were selected to represent a cross-section of immature and established technologies that parallel developments in the hydrogen sector while also highlighting the unique aspects of ammonia production. Energy assumptions for each technology are summarised in Supplementary Table 1, where low temperature electrochemical routes share a harmonised renewable wind electricity source. Technologies were modelled using Aspen HYSYS, where possible, to establish mass and energy balances, with HYSYS files provided in Supplementary Data 2–HYSYS files. The electrochemical properties were determined using the equations specified in the Supplementary Methods 3. Green hydrogen and renewable energy supplies are modelled as steady state.

### Life cycle sustainability assessment framework

The performance of each technology was assessed using an LCSA, which combines a Technoeconomic Analysis or LCC, an LCA, and an SLCA. An overall ranking for each technology was derived by combining the individual rankings from these three strands.

To preserve comparability while reflecting genuine technological distinctions, all pathways share a common cradle-to-plant-gate system boundary. Within this framework, differences in utilities, process efficiencies, and materials are treated as intrinsic features rather than modelling artefacts, with utility assumptions either harmonised (e.g., across low-temperature green routes) or explicitly tied to the defining technology (e.g., grey/blue fossil, red nuclear).

The economic performance was evaluated using a levelised cost of carbon reduction, calculated from the plant’s cash flow to determine a net present value (NPV) as described in supplementary equation 6^39^. Key economic indicators for ranking included CAPEX, OPEX, and the final NPV.

The environmental LCA was conducted using SimaPro software with the Ecoinvent database and the ReCiPe Endpoint I method, which allows the midpoint indicators to be aggregated for use in the LCSA; however immature technologies carry high uncertainties and these results should be treated as relative screening-level indicators rather than precise impact metrics^40^. Inventory data was compiled from literature and is contained in Supplementary Data 1–LCA Inventories, while energy data was based on plant performance modelling (Supplementary Table 2). GWP was determined using midpoint indicators. The environmental emissions were allocated between the primary ammonia product and secondary oxygen byproduct on an economic basis, i.e., the value of the ammonia product as a fraction of the value of the oxygen determined the fraction of the emissions assigned to each. This is detailed in Supplementary Table 18.

The social analysis was based on the United Nations Environment Programme’s SLCA indicators. A literature search was conducted to establish a baseline using data from the top five ammonia-producing countries (PRC, USA, Russia, India, and Indonesia) for the conventional Haber-Bosch process^41^. Abstracts from 200 relevant papers were processed through a custom Large Language Model pipeline to verify their relevance and summarise the relationship between each indicator and green technology, as described in Supplementary Methods 5, with initial SLCA information available in Supplementary Data 3 – SLCA Spreadsheet.

Based on these summaries, technologies were ranked on a 1–5 scale, where 5 indicated a high likelihood of a given social impact and 1 indicated a low likelihood. A neutral ranking of 3 was assigned where the literature showed no strong relationship or if an entire indicator category had limited applicability. These social rankings were held constant across all time periods.

### Future projections and scenarios

Future improvements in electrochemical performance, a key driver of both economic and environmental outcomes, were projected over time. A sigmoidal learning curve, adapted from the method by Odenweller^42^, was used to forecast future values for current density and faradaic efficiency. A Monte Carlo simulation (*n* = 10,000) generated probabilistic development curves, with the median (50th percentile) curve adopted to represent the likely rate of technological progress. Further details are available in Supplementary Methods 2: Technological Advancement. This projection develops our internally consistent scenario projections for 2030, 2050, and 2070.

System-level constraints for CCS, green hydrogen, and renewable energy availability were sourced from policy targets and literature pathways (summarised in Supplementary Table 8). Probabilistic modelling was used to create low, median, and high development scenarios for these constraints. To establish upper and lower bounds for outcomes, the low-constraint scenario was paired with high ammonia technology development, and vice versa.

The growth rates between 2025 and 2050 shown in Fig. 2 are assumed to increase linearly as a scenario simplification, as our conclusions depend on final demand level rather than fluctuations in between. We recognise that real world trajectories are unlikely to be strictly linear but use this as a neutral interpolation.

To account for potential breakthroughs, the analysis also included disruptive step changes. These represent sudden, hypothetical improvements in economic and/or LCA performance that go beyond the gradual learning curve projections. These scenarios are detailed in Supplementary Tables 9–10.

## Supplementary information

Supplementary Information

Description of Additional Supplementary Files

Transparent Peer Review file

## Data Availability

The data generated in this study have been deposited in the Figshare database under (10.6084/m9.figshare.32172879), comprising: Supplementary Data 1 (LCA Inventories), Supplementary Data 2 (HYSYS files), Supplementary Data 3 (SLCA Spreadsheet), and Supplementary Data 4 (Source Data for Fig. 4).
