# Supplementary material for: Ammonia at the net-zero crossroads
Source: Nat Commun. 2026 Jul 21;17:8902. doi: 10.1038/s41467-026-75880-2 (PMC13503741; doi:10.1038/s41467-026-75880-2)
Supplement: Supplementary file 1 [file 41467_2026_75880_MOESM1_ESM.pdf]

Supplementary Information

Ammonia at the Net-Zero Crossroads

Thorin Daniel[1], Qiong Cai[1], Lei Xing[1], Huizhi Wang[2], Xinli Xu[1], Lirong Liu[1]✉, Jin Xuan[1]✉

[1]Faculty of Engineering and Physical Sciences, University of Surrey, Guildford, United Kingdom

[2]Department of Mechanical Engineering, Imperial College London, London, United Kingdom

✉e-mail: lirong.liu@surrey.ac.uk; j.xuan@surrey.ac.uk

Contents

Supplementary Methods ... 3
Supplementary Methods 1: Process Modelling ... 3
Supplementary Methods 2: Technological Advancement ... 10
Supplementary Methods 3: Technoeconomic Analysis ... 12
Supplementary Methods 4: LCA ... 13
Supplementary Methods 5: Social Life Cycle Assessment ... 14
Supplementary Methods 6: Life Cycle Sustainability Assessment Ranking ... 17
Supplementary Methods 7: Ranking ... 18
Supplementary Methods 8: Uncertainty Analysis ... 19
Supplementary Methods 9: Net Zero Pathways ... 20
Supplementary Methods 10: System Constraints ... 20
Supplementary Methods 11: Disruptive Steps ... 21
Supplementary Discussion ... 22
Technology Routes ... 22
Fundamental LCC results ... 26
Fundamental LCA results ... 27
Life Cycle Assessment Hotspot Analysis ... 39
Allocation data ... 40
Fundamental SLCA results ... 40
Gompertz and sigmoidal learning curves ... 42
Technology production Pathways ... 43
Ranking Uncertainty ... 44
Aggregated System Boundary Contributions ... 44
Supplementary References ... 46

Supplementary Methods

Supplementary Methods 1: Process Modelling

Grey, blue, and green ammonia technologies were simulated in Aspen HYSYS to establish comprehensive mass and energy balances (Supplementary Figures 1–3). While the remaining ammonia pathways were also modelled within the HYSYS environment, the dynamic nature of their electrochemical parameters (Supplementary Table 3) and the lack of native electrolysis modelling in HYSYS necessitated a specialized approach. Consequently, these HYSYS models served primarily for mass balance validation, providing a baseline for the manual calculations used in the final analysis.

Process descriptions are available under each Supplementary Figure where details on process and reaction parameters can be found. All models are set to produce 50 kt of ammonia per year, a rate of 6.25 tonnes per hour for the purpose of mass balances and comparison.

Supplementary Table 1 shows the summary of inputs for the routes, with electrochemical performance parameters taken from the future developments, further described in 0.

Here, for the purposes of life cycle assessment and due to its low cost, green ammonia specifically refers to green hydrogen produced using electricity from wind turbines – however future research could easily substitute this for any renewable electricity source or renewable hydrogen pathway, such as biomass.

Supplementary Table 1: Inventory summary and model inputs with conversion and energy based on 2070 data.

	Reactor Feedstock Assumptions	Energy source	Reactor Conversion	Energy Balance
Grey Ammonia	Feedstock mixture of steam and methane for hydrogen production steps, and nitrogen for Harber Bosch ammonia production step	Grid electricity and fossil fuel-based heat	94% (nitrogen moles to ammonia)	8.89[1] (MWh / tonne)

Blue Ammonia	Same feedstock assumptions as grey ammonia	Grid electricity and fossil fuel-based heat	Same conversion assumptions as grey ammonia	8.89[1]
Green Ammonia	Feedstock of green hydrogen and nitrogen	Harmonised renewable electricity	97% (Nitrogen moles to ammonia)	2.24[2]
Red Ammonia	Nitrogen and steam sourced as waste from a high temperature reactor	high temperature heat and electricity from a nuclear source	89-96% (faradaic efficiency)	14.7-14.2 (MWh / tonne)
Yellow Ammonia	Dilute (10%) nitrate ions in water	Harmonised renewable electricity	97-99% (faradaic efficiency)	24.7-23.9 (MWh / tonne)
Orange Ammonia	Pure nitric oxide fed process mixed with water	Harmonised renewable electricity	97-99% (faradaic efficiency)	14.6-14.2 (MWh / tonne)
Turquoise Ammonia	Water for the green hydrogen step and nitrogen for the ammonia step	Harmonised renewable electricity	98% (faradaic efficiency)	4.8 (MWh / tonne)
Purple Ammonia	Water and nitrogen	Harmonised renewable electricity	98% (faradaic efficiency)	17.2 (MWh / tonne)

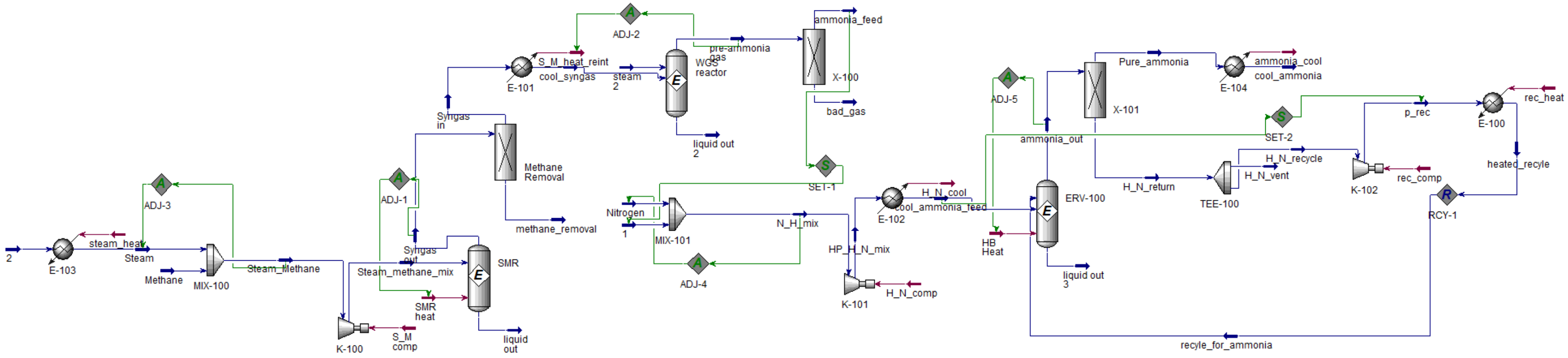

Supplementary Figure 1: HYSYS process diagrams of T1 & T2 (Carbon Capture and Storage (CCS) step not shown) - Grey and Blue ammonia.

The Haber-Bosch production of ammonia shown in Supplementary Figure 1, follows a setup with Steam methane reforming, water gas shift, and ammonia production steps[3]. The reactions here use built-in HYSYS reaction parameters and kinetics.

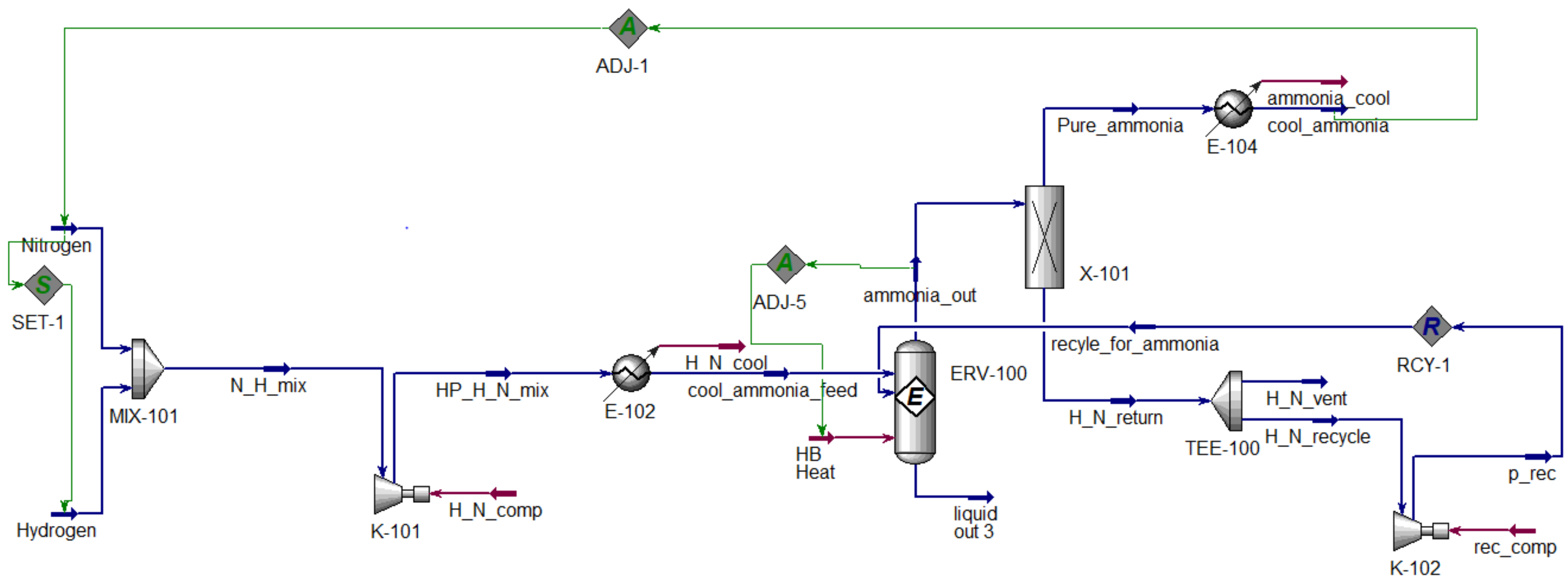

Supplementary Figure 2: HYSYS process diagrams of T3 – Green Ammonia.

The green production of ammonia in Supplementary Figure 2 follows the second half of the ammonia loop presented in Supplementary Figure 1, but with green hydrogen introduced, potentially from an external supplier. In this work we envisage green hydrogen production on industrial clusters nearby to traditional ammonia production plants filling this role.

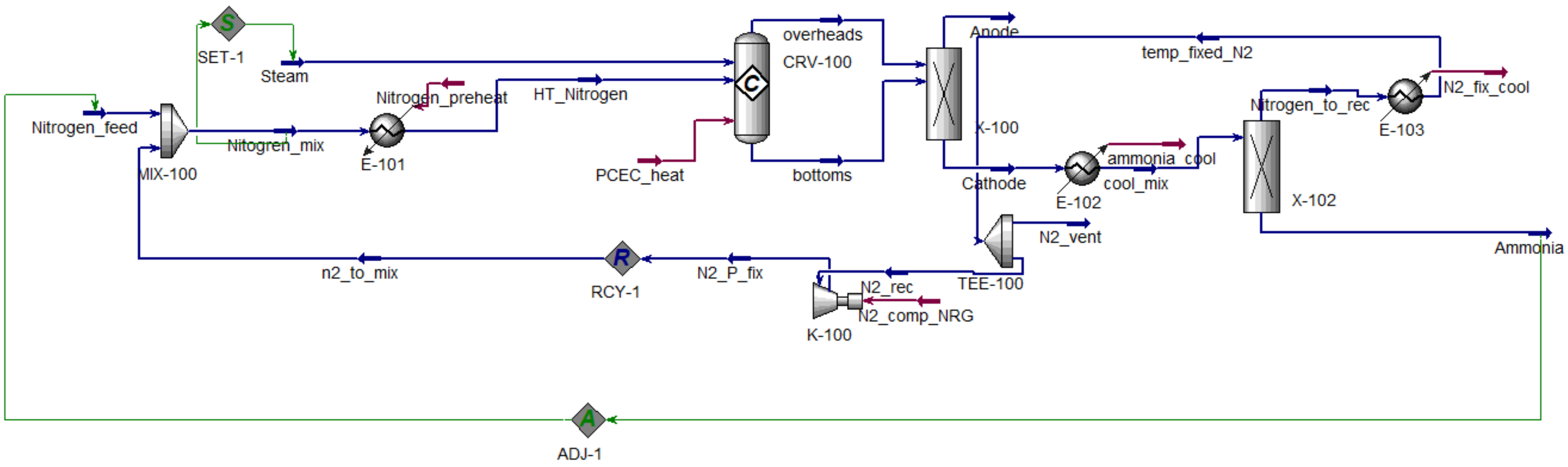

Supplementary Figure 3: HYSYS process diagrams of T4 – Red Ammonia.

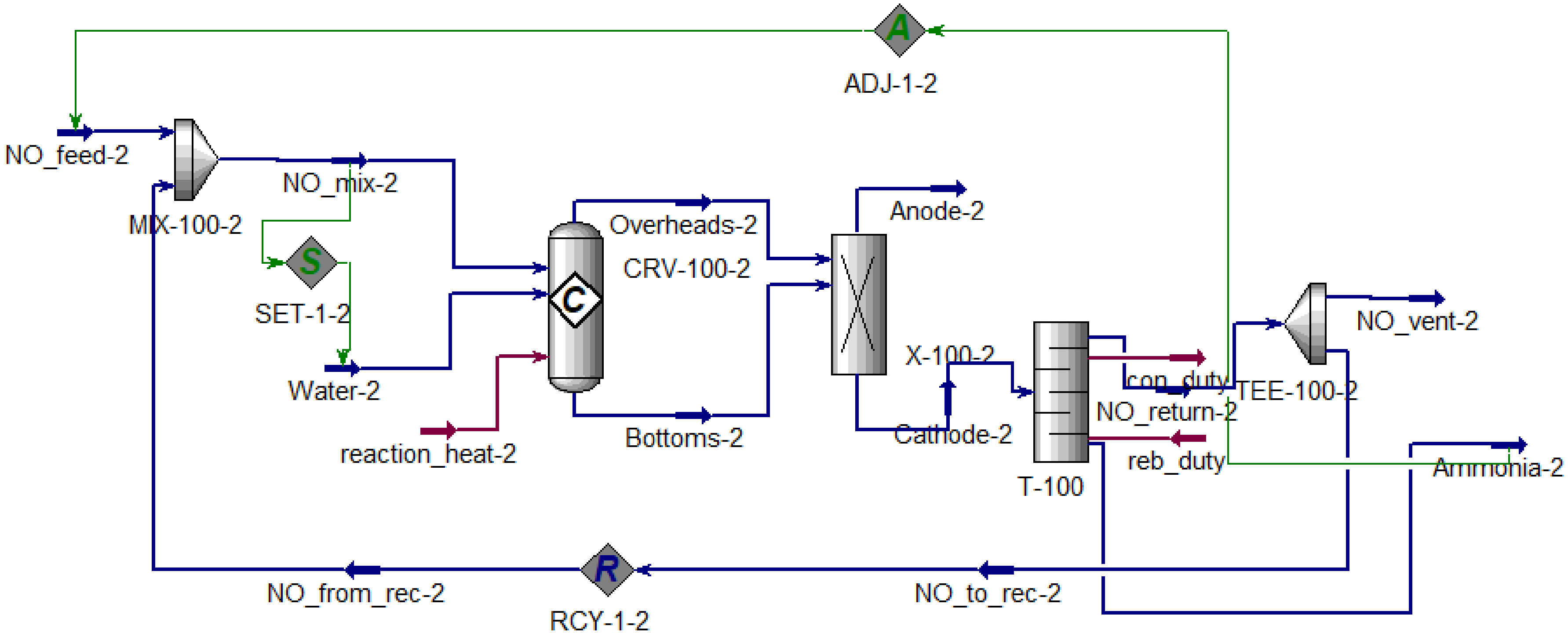

Supplementary Figure 4: HYSYS process diagrams of T5 – Yellow Ammonia.

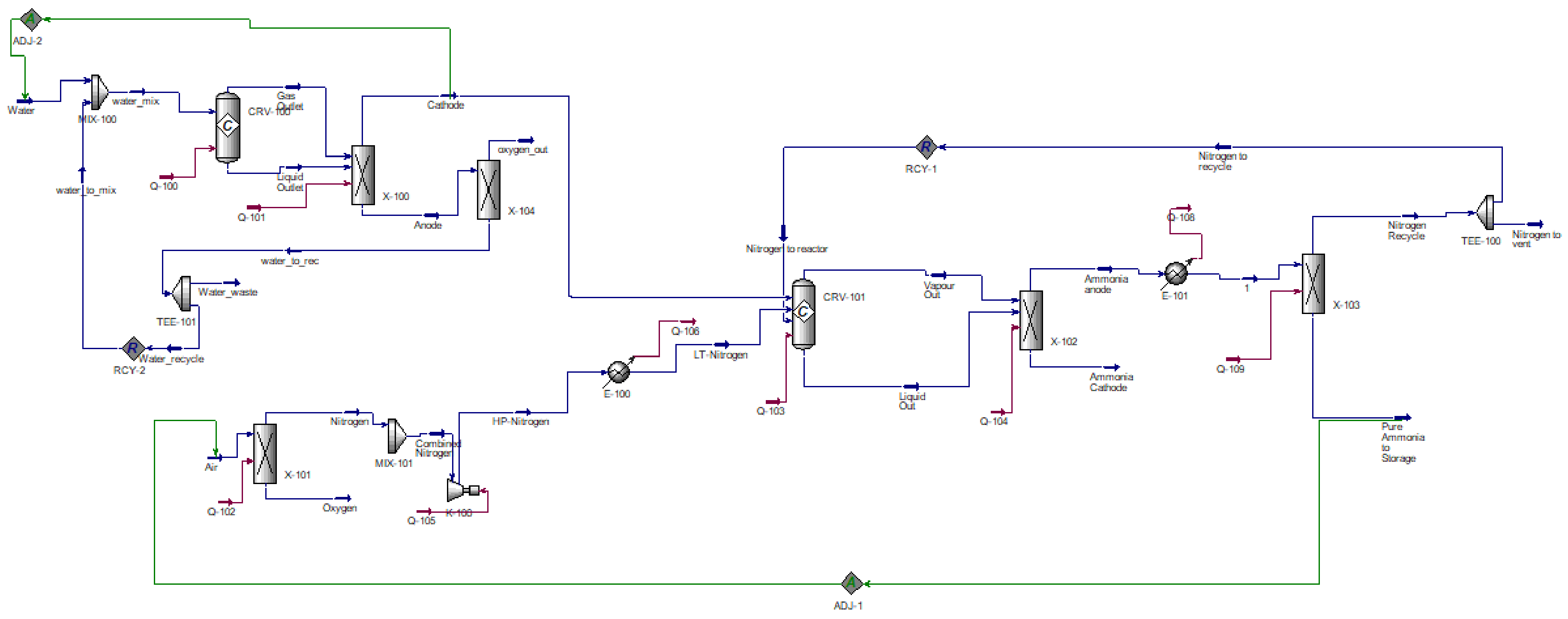

Supplementary Figure 5: HYSYS process diagrams of T7 & T8 (T8 is T7 with hydrogen electrolysis loop section excluded).

Supplementary Methods 2: Technological Advancement

The rate of technological advancement and development is based on starting values, taken from literature, for each technology's current density and faradaic efficiency. The initial values are assumed for 2025 and develop over time, with snapshot values taken for key dates in 2030, 2050, and 2070. The sigmoidal curve which defines the rate of technological development is defined in supplementary equation (1), which is derived from Odenweller et al[4]. This sigmoidal curve is an aggregate of learning by doing and economies of scale but does not explicitly quantify either underlying effect[5,6].

$$X_t(i) = X_t(i-1) + c * X_t(i-1) * \left(1 - \left(\frac{X_t(i-1)}{X_{tmax}(i)}\right)\right) \tag{1}$$

Where X_t is a given parameter X, either current density or faradaic efficiency, at time step, i, and X_{tmax} is the maximum value at time step i. Constant, c, is the rate of development as defined in supplementary equation (2).

$$c = \left(1 + (e^k - 1)\right)^{\frac{1}{resolution}} \tag{2}$$

Where k represents the rate of technological deployment, which is set to vary randomly on a normal distribution with a mean of 20%, truncated to a lower limit of 5%, and a standard deviation of 8%, in line with estimates of electrolyser technology deployment[4,7,8]. Resolution represents the number of calculations per year to create a smooth sigmoidal curve. A resolution of 2 was selected as above this level the curve did not visibly change. The values for faradaic efficiency and current density are given in Supplementary Table 2. Initial faradaic efficiency and current density values for T4-8, found in Supplementary Table 2, are available in literature[9–12]. Targets for faradaic efficiency are assumed to be 100% for all technologies, and current density targets for T4, 7 and 8 are selected to be 1 A cm^{-2} due to their low starting values, and T5 and 6 are targeted 3 A cm^{-2}, mirroring hydrogen electrolysis targets[13].

Due to the rate of technological development, in 2030 only T1-3 are considered mature enough to be viable, by 2050 T1-6 are viable, and all technologies are viable in 2070.

Supplementary Methods 2.1: Learning Rate k Values

Learning rate k values are adapted from literature sources[4,7,8], which estimate traditional renewable technological and unconventional technological learning rates. We bench mark our learning rates against those used for emerging renewable technologies, as there is no empirical data for ammonia technologies, and in particular use the hydrogen electrolyser learning rate of 20%[7,8]. Supplementary Table 2 presents a sensitivity analysis utilizing k values of 15%, 20%, and 25% to demonstrate the resulting effect on the

projected faradaic efficiency for purple ammonia (which assumes a baseline starting efficiency of 10%). It should be noted that the method of calculating faradaic efficiency values is inherently probabilistic and hence will have some natural variations.

Supplementary Table 2: Sensitivity Analysis of k Values of Faradaic Efficiency of Purple Ammonia

	k = 15%	k = 20%	k = 25%
2030	12.7%	13.4%	14.1%
2050	50.9%	57.1%	61.6%
2070	95.1%	97.9%	99.1%

Supplementary Methods 3: Technoeconomic Analysis

The technoeconomic analysis is used to determine the levelised cost of ammonia through a cash flow analysis of the cost of material for an electrolyser, the balance of plant, the cost of energy and material flow through plant, and the sale price of ammonia. This is achieved via the equations below.

Key electrochemical calculations include:

$$E_{mol} = \frac{VFn}{FE \cdot 3.6 \times 10^6} \tag{3}$$

Where E_{mol} is the specific energy (kWh mol^{-1}), V is the voltage, F is Faraday's constant (96485.15 sA mol^{-1}), n is the number of electrons per ion (6 for Nitrogen reduction, 8 for Nitrate reduction, and 5 for NO reduction), and FE is the faradaic efficiency. The calculation includes a units' adjustment from joules to kilowatt hours.

$$E_{mass} = \frac{E_{mol} \times 1000}{Mr_{NH_3}} \tag{4}$$

Where E_{mass} is the specific energy (MWh $tonne^{-1}$) and Mr_{NH3} is the molecular mass of ammonia. The calculation includes a units' adjustment from grams to kilograms.

$$Area_{cell} = \frac{\dot{m}Fn}{JMr_{NH_3}} \tag{5}$$

Where $Area_{cell}$ is the cell area (m^2), $\dot{m}$ is ammonia production rate (g s^{-1}), and J is the current density (A m^{-2}). Once the cell area is calculated the material requirement of the PEM cell can be calculated and hence the capital expenditure (CAPEX) and material operating expenditure (OPEX). This method is fully derived in Daniel et al.[14]. For solid oxide and anion exchange membrane cells a simplified costing of \$2000 and 125 kW^{-1} is used, as these more immature technologies make detailed material, balance of cell, and balance of plant calculations redundant. Currency unit is US dollars (\$). For AEM and SOEC cells we assume that the current density must be within the nominal operating range for the cell before its economics can be considered valid, as the cost per kWh will not hold true until the energy density per material area (and hence cost area) is within a valid range [13].

Once the CAPEX and OPEX have been determined, the cashflow in year t, CF_t (\$), can be calculated and the net present value, NPV, determined according to eq S6.

$$NPV = \sum \frac{CF_t}{(1+i)^t} \tag{6}$$

Where i is the interest rate (%), and t is the year in the lifetime of the plant, assumed to be 0-25. The weighted average cost of capital (WACC) for global wind energy projects is used as the interest rate initially and then reduced as the technologies develop. At an NPV of

exactly 0 the project is at its minimum feasibility, and the levelised cost of ammonia (LCOA) can be calculated – which represents the minimum possible sale price of ammonia for the project to feasible.

The technologies are assessed on the values of their CAPEX, OPEX, and LCOA values, where lower was better. This allows a ranking for each technology in each category.

Supplementary Methods 4: Life Cycle Assessment

The LCA is performed as a cradle to gate assessment of ammonia technologies, using a functional unit of one tonne of ammonia produced.

The life cycle assessment (LCA) is performed using SimaPro and the Ecoinvent database. Inventories for each of the technologies are assembled from literature data and their energy consumption is calculated according to Supplementary Table 2, allowing the impacts of each technology to be assessed. The full inventories for each technology can be found in Inventories.xlsx.

Each technology was assessed using the Recipe 2016 Endpoint (I) set of LCA indicators to provide a complete range of ways to understand the environmental impacts. Endpoint was selected to allow the groupings of indicators to prevent the ranking from being overweighted towards a given set of indicators. Individualist (I) was chosen over the other options as this is more suited for highly immature technologies which will have a huge amount of development over the time frames, as we expect T2-8 to have a large amount of development in the periods 2030-2050, and 2050-2070 [15]. The three grouped categories, Human Health, Ecosystems, and Resources are given a comparative score for each technology, allowing a ranking for each technology in each category.

The complete set of Recipe Endpoint (I) midpoint and endpoint indicators are available in Supplementary Table 3 and 4. The relationship between midpoint and endpoint indicators is available in [15].

Supplementary Table 3: Midpoint Indicators

**Impact category**	**Unit**
Global warming, Human health	DALY
Global warming, Terrestrial ecosystems	species.yr
Global warming, Freshwater ecosystems	species.yr
Stratospheric ozone depletion	DALY
Ionizing radiation	DALY
Ozone formation, Human health	DALY
Fine particulate matter formation	DALY
Ozone formation, Terrestrial ecosystems	species.yr
Terrestrial acidification	species.yr
Freshwater eutrophication	species.yr
Marine eutrophication	species.yr

Terrestrial ecotoxicity	species.yr
Freshwater ecotoxicity	species.yr
Marine ecotoxicity	species.yr
Human carcinogenic toxicity	DALY
Human non-carcinogenic toxicity	DALY
Land use	species.yr
Mineral resource scarcity	USD2013
Fossil resource scarcity	USD2013
Water consumption, Human health	DALY
Water consumption, Terrestrial ecosystem	species.yr
Water consumption, Aquatic ecosystems	species.yr

Supplementary Table 4: Endpoint Indicators

**Damage category**	**Unit**
Human Health	DALY
Ecosystems	species.yr
Resources	USD2013

Supplementary Methods 5: Social Life Cycle Assessment

The social life cycle assessment (SLCA) is performed using the full set of United Nations Environment Programme's (UNEP) SLCA social indicator categories, with one indicator selected per subcategory. The SLCA is split into two portions – firstly, creating a baseline rating for each indicator for technology 1, the widely used and highly mature Haber-Bosch process for ammonia production. This is achieved through assessing the performance of the top five ammonia producing countries (PRC, Russia, USA, India, and Indonesia) against each indicator using widely available data. T1 was scored on a scale of 1-5, 1 being highly unlikely and 5 being highly likely, to create a baseline of a mature real-world technology, as T2-8 do not represent technologies currently widely available for analysis.

The second phase of the SLCA was to use a large language model (LLM) retrieval augmented generation (RAG) pipeline to create an AI assisted literature search for literature associated with both the indicator and green technologies. Search terms were generated and input to both Scopus and web of science where the top 100 results' abstracts were downloaded (maximum 200 total per indicator). Search terms were chosen to return the maximum relevant number of papers, and if less than 100 results were returned all available papers were selected. Abstracts were selected instead of full papers, as the ability to download them automatically is available through both Scopus and web of science, whereas manually downloading 200 papers per indicator and extracting their text from pdfs would take an excessive amount of time. By extracting many abstracts in an easily machine-readable format, we show that a large amount of concise, relevant, and well formatted data can be obtained in a minimal amount of time.

The abstracts were then passed through a custom LLM pipeline which called OpenAI's ChatGPT 4o to assess each abstract for relevance to the desired indicator. Papers which were irrelevant were discarded and the remaining abstracts were passed again into the model pipeline to summarise their contents. According to UNEP, each subcategory can relate positively or negatively to a stakeholder category - i.e., freedom of association relates positively to the worker and should be encouraged, and child labour relates negatively and should be limited[16]. Based on the UNEP methodology and these summaries the authors develop a set of criteria for ranking each technology, T2-8, on a 1-5 scale based on their likely interaction with the given indicator. Supplementary Figure 6 displays a flowchart of the LLM RAG assisted SLCA process. Supplementary Table 5 shows the ranking and criteria for being assigned that ranking. Supplementary Table 6 shows the full list of UNEP SLCA subcategories and indicators used to assess the technologies. Indicators 23, 36, and 40 did not yield enough abstracts for use in the LLM pipeline, due to the unrelated nature of the indicators and the technologies, and the technology rankings were extrapolated from the baseline.

Supplementary Table 5: Criteria for Social Life Cycle Assessment (SLCA) ranking.

Rank	Criterion
1	Strong positive relationship between indicator and technology or technologies mentioned in summary. Indication that the technology is likely to cause effects positively associated with the indicator.
2	Weak positive relationship between indicator and technology or technologies mentioned in summary. Some indication that the technology is likely to cause effects positively associated with the indicator.
3	Neutral relationship between indicator and technology or technologies mentioned in summary. No indication that the technology is likely to be influenced by or influence the indicator.
4	Weak negative relationship between indicator and technology or technologies mentioned in summary. Some indication that the technology is likely to cause effects negatively associated with the indicator.
5	Strong negative relationship between indicator and technology or technologies mentioned in summary. Indication that the technology is likely to cause effects negatively associated with the indicator.

Supplementary Table 6: Social Life Cycle Assessment (SLCA) subcategories and indicators

	Category/subcategory	Indicator
	**Worker**	-
1	Freedom of association and collective bargaining	Likelihood of trade unions being involved with the technology
2	Child labor	Likelihood of working children under the legal age or 15 years old (14 years old for developing economies (%))

3	Fair salary	Likelihood of employees earning wages below poverty line
4	Working hours	Likelihood of number of hours effectively worked by employees being above baseline
5	Forced labor	Likelihood of forced labour
6	Equal opportunities/ discrimination	Likelihood of gender inequality in labour force
7	Health and safety	Likelihood of health and safety standards exceeding base
8	Social benefits/ social security	Likelihood of worker social benefits (SDG 1.3.1 coverage)
9	Employment relationship	Likelihood of well-defined workers' rights in a contract
10	Sexual harassment	Likelihood of sexual harassment in the workplace
11	Smallholders including farmers	Likelihood of a positive effect on smallholders
	**Local community**	
12	Access to material resources	Likelihood of material resource use exceeding baseline
13	Access to immaterial resources	Likelihood of repressing protest against technology
14	Delocalization and migration	Likelihood of use of migrant workers in workforce
15	Cultural heritage	Likelihood of repression of religious freedom
16	Safe and healthy living conditions	Likelihood of low quality of living conditions
17	Respect of indigenous rights	Likelihood of indigenous rights being taken into account when building technology
18	Community engagement	Likelihood of civic engagement
19	Local employment	Likelihood of use of migrant workers in workforce
20	Secure living conditions	Likelihood of human rights abuses by states forces
	**Value chain actors**	
21	Fair competition	Likelihood of no high-quality antitrust laws
22	Promoting social responsibility	Likelihood of no corporate social responsibility
23	Supplier relationships	Likelihood of coercive action in supply chain
24	Respect of intellectual property rights	Likelihood of poor respect of intellectual property rights
25	Wealth distribution	Likelihood of poor wealth distribution within the supply chain
	**Consumer**	
26	Health and safety	Likelihood of consumer health and safety events
27	Feedback mechanism	Likelihood of poor-quality response in utilising feedback mechanisms
28	Consumer privacy	Likelihood of poor consumer privacy
29	Transparency	Likelihood of poor transparency
30	End-of-life responsibility	Likelihood of extended producer responsibility
	**Society**	
31	Public commitment to sustainability issues	Likelihood of poor commitment to sustainability
32	Contribution to economic development	Likelihood of poor contribution to GDP
33	Prevention and mitigation of armed conflicts	Likelihood of contribution to an armed conflict

34	Technology development	Likelihood of low technological development
35	Corruption	Likelihood of organisational corruption
36	Ethical treatment of animals	Likelihood of poor treatment of animals
37	Poverty alleviation	Likelihood of poor contribution to reduction of poverty
	**Children**	
38	Education provided in the local community	Likelihood of low-quality local education
39	Health issues for children as consumers	Likelihood of poor impact on children's health
40	Children concerns regarding marketing practices	Likelihood of marketing to children.

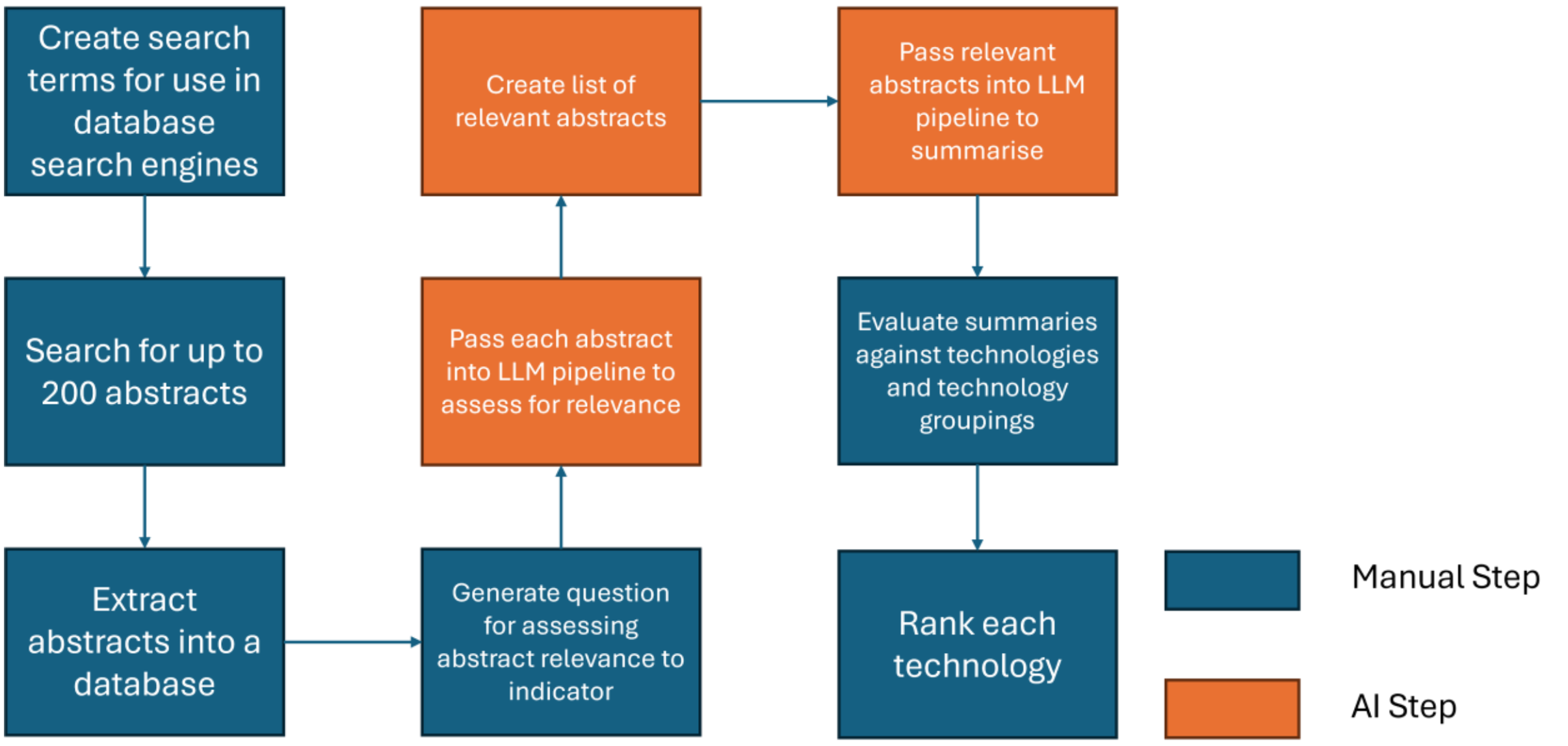

Supplementary Figure 6: Large Language Model (LLM) Retrieval Augmented Generation (RAG) assisted Social Life Cycle Assessment (SLCA) flow diagram

Supplementary Methods 6: Life Cycle Sustainability Assessment Ranking

In order to create a master Life Cycle Sustainability Assessment (LCSA) ranking, category rankings for each of the three categories were created using the indicators for each technology. Across each of the three strands of the LSCA, indicators are setup in such a way that a lower numeric value always indicates a better performance.

A TOPSIS ranking is used for the category rankings and the result of each category ranking is then ranked to create a master LCSA ranking. TOPSIS is a widely used multi-criteria decision making (MCDM) method which ranks based on a mathematical distance from an ideal solution[17]. Each indicator within a category and each category is equally weighted so that no preference is given to one over any other[18]. The full indicator rankings can be seen in Supplementary Figure 18.

Supplementary Methods 7: Ranking

A Technique for Order of Preference by Similarity to Ideal Solution (TOPSIS) ranking with min/max normalisation is applied to form the ranking shown in Figure 1[19].

The TOPSIS evaluates m alternatives across n criteria by identifying the option with the shortest geometric distance to the positive ideal solution (PIS) whilst maintaining the longest distance from the negative ideal solution (NIS).

Prior to calculating these distances, the raw decision matrix $X = \left[x_{ij}\right]_{m\times n}$ must be standardised. Because criteria frequently operate on disparate scales, min-max normalisation is applied to linearly map all values to a dimensionless scale strictly between 0 and 1. For a beneficial criterion (where higher values are preferred), the normalised score r_{ij} is calculated as supplementary equation7:

$$r_{ij} = \frac{x_{ij} - \min_i x_{ij}}{\max_i x_{ij} - \min_i x_{ij}} \tag{7}$$

For non-beneficial criteria, the numerator is adjusted to $\max_i x_{ij} - x_{ij}$ to ensure that lower raw values yield higher normalised scores.

These normalised values are subsequently multiplied by predetermined criteria weights to form a weighted decision matrix, from which the PIS and NIS are defined. The n-dimensional Euclidean distances (D_i^+, D_i^-) from each alternative to both the PIS (v_j^+) and NIS (v_i^-) are computed. Following normalisation, predetermined criteria weighting is set to be equal for all criteria. Finally, a relative closeness coefficient (C_i) is derived to aggregate these distances into a comprehensive performance score:

$$C_i = \frac{D_i^-}{D_i^+ + D_i^-} \tag{8}$$

$$D_i^+ = \sqrt{\sum_{j=1}^{n}\left(v_{ij} - v_j^+\right)^2} \tag{9}$$

$$D_i^- = \sqrt{\sum_{j=1}^{n}\left(v_{ij} - v_j^-\right)^2} \tag{10}$$

The resultant values of C_i lie within the interval [0, 1], where v_{ij} are the weight normalised score. Alternatives are then ranked in descending order of their closeness coefficients, establishing the final order of preference.

A recognised limitation of TOPSIS is rank inversion (or rank reversal), where adding or removing an alternative alters the relative ordering of the existing options. Under min-max

normalisation, this phenomenon occurs because the scaling relies strictly on the dataset's empirical boundary values. Introducing an alternative that expands a criterion's range changes the maximum ($max_i\, x_{ij}$) or minimum ($min_i\, x_{ij}$) values, altering the normalisation denominators for the entire matrix. Consequently, this shifts the coordinates of the positive and negative ideal solutions, recalibrating the Euclidean distances and potentially reordering the final closeness coefficients (C_i). For example in the main paper Fig 2c, initially, blue ammonia is ranked above grey for LCA; however, it performs significantly worse than grey regarding resource use. In 2050, the addition of new, higher-performing technologies fundamentally shifts the theoretical ideal. This shifts the weighting of the distance-to-target metric, causing a rank inversion between blue and grey within the ranking methodology.

Supplementary Methods 8: Uncertainty Analysis

The uncertainty of the final rankings is due to the uncertainty of the data in each of the strands of the assessment.

In the techno-economic analysis of life cycle costing there is an inherent uncertainty in the electrochemical factors which ultimately partially govern the LCOA. However, in developing a chemical plant there are also myriad other factors affecting cash flow and to capture this uncertainty we apply an up to 50% change in OPEX, CAPEX, and consequent LCOA[20], as shown in Supplementary Figure 21a. We then perform a Montecarlo simulation (n=10,000) with the economic factors randomly affected by this 50% change and determine the variance in the LCC ranking.

The Ecoinvent database has inbuilt uncertainty and a Montecarlo simulation (n=10,000) allowed us to determine the uncertainty level for each midpoint indicator, as shown in Supplementary Figure 21b, which then affects endpoint in the LCA ranking. Hence, the variance in ranking could be determined. The 95% confidence limits show that the endpoint indicators can vary by up to 50% or more around the mean values.

The SLCA's uncertainty is due to the inherent interpretability of results of the LLM assisted literature, wherein an assessment of the correlation between a technology or grouping of technologies and an indicator could result in a discrepancy of scoring of up to one point. To address this score uncertainty, we create a Montecarlo simulation (n=10,000) where the scoring of each technology in each indicator is able to vary by 1 point and then rank each simulation run to create the ranking distribution and calculate its variance.

Once the variance for each strand of the triple helix assessment is determined, the overall LCSA ranking can be assessed through a Montecarlo simulation (n=10,000) by allowing the individual variance to inform the overall ranking. This results in Supplementary Figure 21c, where technologies show regions of likely ranking.

Supplementary Methods 9: Net Zero Pathways

The technology development pathways used to create Figure 2 involve assuming different technology ratios based on stated policies for each location, and learning rates based on the policy commitment to a given technology. The mathematical equation governing this is given in Supplementary Methods 2: Technological Advancement, supplementary equations (1 & 2), but the specific inputs for k - the growth constant, technologies and their starting ratio, are summarised in Supplementary Table 7.

Supplementary Table 7: Location technology ratios and k values.

	Technology ratio	$\boldsymbol{k}$
USA	100% Blue	0.25
EU	3:1 Green:Blue	0.5
PRC	2:3 Green:Blue	0.25

The reasons for the technology ratios are discussed in the main paper, but the values of k reflect the strength of policy commitment to the discussed technologies. USA and PRC are assigned 0.25 as their policies are more equivocal favouring traditional technologies and carbon capture, whereas the EU has a strong environmental and economic commitment to green hydrogen and is assigned a 0.5[21–23]. The fast, slow, and medium roll-out scenarios represent the 5th and 95th percentiles and median values for deployment when k is allowed to vary normally.

Supplementary Methods 10: System Constraints

Current values and targeted values for 2050/60 are required to inform the system constraint calculations. These are summarised in Supplementary Table 8.

Supplementary Table 8: Starting capacity (left box) and targeted capacity (right box) for USA, EU, and PRC Carbon Capture and Storage (CCS), Green Hydrogen, and Renewable Electricity.

	CCS (Mt yr^{-1})		Green Hydrogen (Mt yr^{-1})		Renewable Electricity (GW)	
USA	22.1[24]	1000[25]	-	-	-	-
EU	3.345[24]	550[26]	0.075[27]	33.52[22]	266.4[28]	926[22]
PRC	4.06[24]	1500[29]	0.47[30]	56.47[31]	1026[28]	7000[32]

USA has no targets for green hydrogen and renewable electricity as, due to the 2025 change in administration, it has little to no federal policy to support development in these areas, and even before 2025 had a much greater focus on CCS. Values in Supplementary Table 6 are convert to Mt yr^{-1} and GW as required from electrolyser capacity by assuming 8000 working hours per year and an energy density of 42.5 MWh $tonne^{-1}$ for green hydrogen or from total energy produced in TWh.

Supplementary Methods 11: Disruptive Steps

The disruptive steps of Figure 4 are based on groups of sudden changes in technology of conditions, summarised in Supplementary Table 9 & 10.

Supplementary Table 9: Economic step changes for T8

Step	Faradaic Efficiency (%)	Electricity cost ($ MWh^{-1}) and Voltage (V)	Interest rate (%)	Current density (A cm^{-2})
Value change	67 -> 99	20 -> 10<br>1.5 -> 1.3[33]	5 -> 2	0.14 ->1[13]

Supplementary Table 10: Life Cycle Assessment (LCA) step changes for T8

Step	Material Usage	Faradaic Efficiency (%)	Voltage (V)	Lifespan (1000 hours)	Water Waste %
Value change	Various changes to PEM electrolyser[34]	67-99	1.5 -> 1.3	40 -> 80	20 -> 0

Supplementary Discussion

Technology Routes

In the main body we refer to each ammonia technology route by its colour, here we refer to them as technology (T) 1-8 for brevity.

In this work we define the eight mainstream ammonia technologies, ranging from mature established technologies in the Haber-Bosch process to highly immature, for this purpose, technologies in low temperature membrane electrolysers[33]. The Haber-Bosch process was first developed over 100 years ago as a source of ammonia, which currently supports the food production of approximately one third of the global population and has remained largely the same since its inception[35]. This highly energy intensive process is responsible for a significant proportion of global carbon emissions and, if it is to support the growing global population, is in need of decarbonising and electrification[36,37]. Here we use the Haber Bosch process as a baseline technology to compare alternative ammonia production technologies to, which are summarised in Supplementary Table 1.

Technology 2 is an effective way to reduce carbon emissions from ammonia production, retrofitting carbon capture and storage (CCS) technology to existing ammonia infrastructure to largely reduce direct carbon emissions, both from the ammonia plant itself and from any electricity required by the plant[1]. CCS technology is limited in its carbon reduction potential and here we assume an 90% reduction of direct CO_2 emissions based on a post combustion capture technology[38]. Blue hydrogen technology is expected to fall roughly 10% by 2050, due to its high level of maturity and we assume a similar trend for the CAPEX of blue ammonia through its use of blue hydrogen[39].

These two technologies are defined to represent real world production routes where both heat and electricity are carbon intensive. Grey and blue therefore use fossil-based steam and globally representative grid electricity, consistent with existing industrial plants.

Technology 3 combines green energy electrolyser-based hydrogen production, an increasingly mature but still over expensive hydrogen production technology, with the thermochemical Haber-Bosch ammonia production step[14,40]. This technology addresses the carbon intensive steam-methane reforming step in ammonia production but does not address the high temperature, energy intensive ammonia production step. Here we model the hydrogen production step as being represented by a levelised cost of hydrogen, which encapsulates the combined cost and energy usage of a hydrogen production plant without needing to predict the individual technologies' development, simply the single cost of hydrogen as it changes[14]. This ammonia route assumes an electrified Haber–Bosch process with hydrogen from renewable electrolysis; process

electricity is renewably supplied and heat demand is met from this electrified configuration.

Technology 4 represents the possibility of a high temperature electrochemical ammonia production route, whereby high temperatures are provided by a symbiotic high temperature process, which we here assume to be a nuclear reactor providing both the heat and electricity[41]. Both solid oxide and proton ceramic electrolysers have been considered for this process. In this study, we use solid oxide electrolysis cell (SOEC) as an example for this type of technology and believe that the proton ceramic electrolysis cells (PCEC) and the SOEC analysis would be similar[9].

Technology 5 represents a potential solution to a major water pollution issue through the removal of nitrate ions into ammonia, or through conversion of nitrate ions from other sources[42]. Ammonia run-off from farmland into waterways can result in eutrophication and seriously affect the ecosystem therein, and removal of nitrate ions could help to prevent this and reform ammonia fertiliser in the process[43]. Technology 6 also focuses on the conversion of nitrogen waste, in this case gaseous nitric oxide, to ammonia, through a low temperature anion exchange membrane (AEM) electrolyser[44]. Both nitrogen waste reduction routes are highly active compared to nitrogen reduction, demonstrating promising initial pathways for ammonia production[11,45].

Technology 8 represents a pathway for direct electrochemical reduction of nitrogen with water for ammonia synthesis[33]. This pathway is desirable but faces challenges including ammonia selectivity over hydrogen production and the sluggish kinetics of the 6-electron reaction[46]. An alternative involving first evolving hydrogen to be reaction with gaseous nitrogen is shown in technology 7, which can be considered a two-step direct ammonia electrolysis route[12]. A PEM cell is considered for both technology 7 and 8 due to the wide availability of data on PEM water electrolysis and the assumption of parity between mature electrochemical ammonia and hydrogen synthesis routes[12].

Technologies routes are only categorised into Supplementary Tables 12 – 15 once they reach a cost level competitive with grey ammonia, through their technological advancements.

The cost for T1 is treated as approximately constant over time as Supplementary Table 12 – 15 show, due it being a mature technology with limited scope for further cost reduction. T2 shows only small cost reductions over time as this is also a relatively mature technology. T3 follows cost reductions over time due to the predicted fall in price of green hydrogen and electricity[14]. Other routes also benefit from decreases in electricity price, however their costs are also governed by a combination of by current density, faradaic efficiency, cell area, and balance of plant factors. Electrolyser lifetime is specified at 40,000 hours from 2050 for the immature electrochemical routes, however if this lifetime cannot be achieved these routes would be disadvantaged even further with a consequent

increase in capital and material replacement costs, compared to T1-T3, potentially pushing their availability even further and costs higher.

Utility comparisons are essential for ensuring fair comparison between technologies. Grey and Blue ammonia technologies are defined per real world usage, i.e. heat and energy are carbon intensive, whereas green ammonia assumes an electrified process with renewably generated hydrogen. Heat for red ammonia is assumed from a high temperature industrial source i.e., nuclear, which also supplies the electricity. The remaining low temperature processes are all assumed to be renewably powered using wind. Grid electricity varies by region, but Ecoinvent processes have a weighted average used and renewable power is assumed from renewable wind across all low temperature processes. These differences, in addition to the process efficiency and material differences, form part of the basis of the comparison between technologies. Biomass electricity is not considered, for renewable purposes but could be in future a distinct pathway – allowing the ammonia rainbow to evolve and expand in the same way the hydrogen rainbow has. For green hydrogen and renewable energy production we assume steady state supplies and do not explicitly simulate intraday variability or storage.

Supplementary Table 11: Description of technologies T1-8.

T	Colour	Description	Nitrogen Source	Hydrogen Source	Heat	Electricity	Reactor Type	Ref
1	Grey	HB Process	Air	Grey SMR	High Temp	Grey	Chemical	[37]
2	Blue	HB Process with CCS	Air	Blue SMR	High Temp	Blue	Chemical	[1]
3	Green	Electrified HB	Air	Green	High Temp	Green	Chemical	[40]
4	Pink	High Temperature electrolysis Ammonia	Air	Steam	High Temp (Nuclear Symbiosis)	Green (Nuclear)	SOEC	[41]
5	Yellow	NO fed electrolysis	NO	Water	Low Temp	Green	AEM Electrolyser	[42]
6	Orange	Nitrate fed electrolysis	NO_3^-	Water	Low Temp	Green	AEM Electrolyser	[45]
7	Turquoise	Two Step electrolysis Ammonia	Air	Green	Low Temp	Green	PEM Electrolyser	[12]
8	Purple	Single Step electrolysis Ammonia	Air	Water	Low Temp	Green	PEM Electrolyser	[46]

Supplementary Table 12: 2025 starting electrochemical parameters

2025								
	T1	T2	T3	T4	T5	T6	T7	T8
CD (A/cm2)	\	\	\	0.001	0.109	0.017	0.001	0.001
FE (%)	\	\	\	75%	93%	93%	10%	25%

Supplementary Table 13: 2030 Technology Route Operating Parameters

2030			
	T1	T2	T3
LCOA ($/tonne)	444	722.7	623.4
CAPEX ($M)	48.2	57.8	72.7
OPEX ($M / year)	16.6	29.4	25.2
H2 price ($/kg)	\	\	2.5
O2 sell price ($/tonne)	\	\	50
WACC (%)	7.5%	7.5%	5.0%
CAPEX reduction (%)	\	5%	\
Energy (MWh / tonne)	\	\	11.9
Technology Readiness Level (TRL)	9	9	8

Supplementary Table 14: 2050 Technology Route Operating Parameters

2050						
	T1	T2	T3	T4	T5	T6
LCOA ($/tonne)	444	684.7	451.5	549.6	490.5	441.9
CAPEX ($M)	48.2	54.8	72.7	15.9	23.1	13.7
OPEX ($M / year)	16.6	27.9	16.6	26.2	28.5	26.4
CD (A/cm2) [90% confidence interval]	\	\	\	0.118 [0.008-0.542]	1.509 [0.542-1.892]	0.973 [0.123-1.807]
V (V)	\	\	\	0.8	1.9	1.8
FE (%) [90% confidence interval]	\	\	\	89.3% [85.2-90.8]	97.0% [96.0-97.4]	97.0% [96.0-97.4]
H2 price ($/kg)	\	\	1.5	\	\	\
O2 sell price ($/tonne)	\	\	50	50	\	\
Electricity cost ($/kWh)	\	\	\	0.02	0.02	0.02
WACC (%)	7.5%	7.5%	5.0%	5.0%	5.0%	5.0%
CAPEX reduction (%)	\	10%	\	0.9	0.2	0.2
Capacity (MW)	\	\	\	53	154.1	91.3
Energy (MWh / tonne)	\	\	11.9	14.7	24.7	14.6
Lifetime (thousand hours)	\	\	\	40	40	40
TRL	9	9	9	5/6	6/7	6/7

Supplementary Table 15: 2070 Technology Route Operating Parameters

2070								
	T1	T2	T3	T4	T5	T6	T7	T8
LCOA ($/tonne)	444	646.6	365.5	481.4	472.9	231.7	574.1	410.8
CAPEX ($M)	48.2	51.7	72.7	14.2	22.4	13.3	18.0	18.0
OPEX ($M / year)	16.6	26.3	12.3	22.9	27.7	16.4	30.8	22.6
CD (A/cm2) [90% confidence interval]	\	\	\	0.886 [0.043-0.997]	2.925 [1.652-2.993]	2.875 [0.569-2.992]	0.886 [0.043-0.997]	0.886 [0.043-0.997]
V (V)	\	\	\	0.8	1.9	1.8	0.5	1.5
FE (%) [90% confidence interval]	\	\	\	99.6% [96.4-99.9]	99.9% [99.1-100.0]	99.9% [99.1-100.0]	97.8% [93.2-99.8]	98.4%
H2 price ($/kg)	\	\	1	\	\	\	1	\
O2 sell price ($/tonne)	\	\	50	50	\	\	50	\
Electricity cost ($/kWh)	\	\	\	0.02	0.02	0.02	0.02	0.02
WACC (%)	7.5%	7.5%	5.0%	5.0%	5.0%	5.0%	5.0%	5.0%
CAPEX reduction fraction (-)	\	15%	\	0.9	0.2	0.2	0.75	0.75
Capacity (MW)	\	\	\	47.3	149.6	88.5	30.11	90
Energy (MWh / tonne)	\	\	11.9	14.2	23.9	14.2	4.82	17.2
Lifetime (thousand hours)	\	\	\	40	40	40	40	40
TRL	9	9	9	7-9	7-9	7-9	7-9	7-9

Fundamental Life Cycle Costing Results

Fundamental CAPEX, OPEX, and LCOA values can be found along with their relevant input values in Supplementary Table 12-15. This results in the raw data and ranking shown in Supplementary Figure 7.

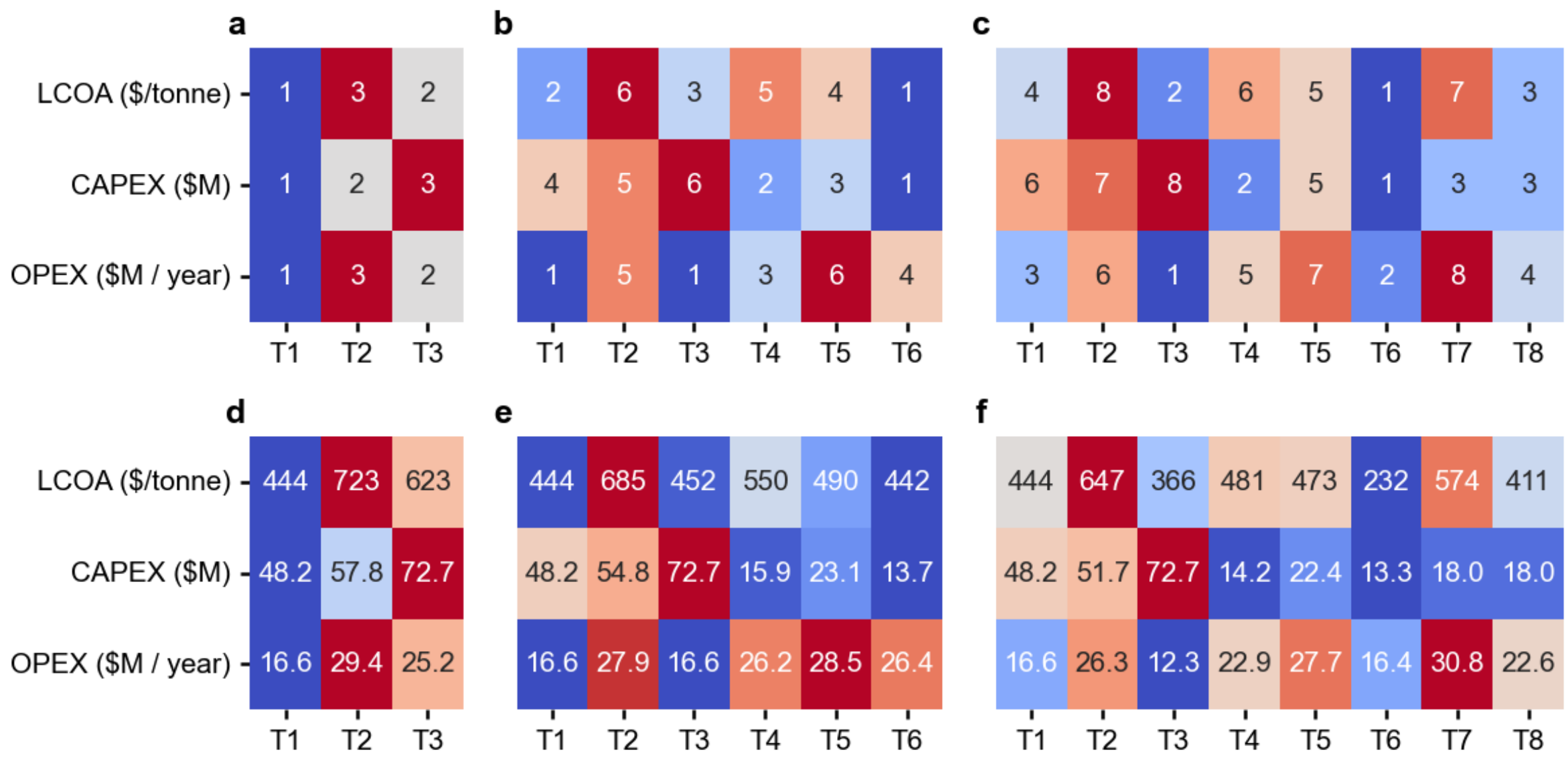

Supplementary Figure 7: Life Cycle Costing (LCC) indicator rankings for a) 2030 b) 2050 c) 2070, and raw data (d-f).

Fundamental Life Cycle Assessment Results

Initial LCA effects can be assessed via the indicators assembled in Supplementary Table 3, as shown in Supplementary Figure 8&9 for 2030. The endpoint indicator assessment can be seen in Supplementary Figure 10. This results in the ranking seen in Supplementary Figure 17a. The same pattern for 2050 and 2070 can be seen in Supplementary Figures 11-13, 14-16, and 17b&c. Emissions are allocated solely to the ammonia generated in cases without oxygen generation (T1, T2), those with oxygen generation have emissions allocated on an economic value basis.

Both midpoint characterisation and endpoint indicators are presented in this analysis. The ReCiPe endpoint LCA method translates environmental interventions into specific damage pathways. For example, rather than simply reporting the Global Warming Potential (GWP) of emissions in CO_2 equivalents, as a standard midpoint method does. Instead, the endpoint approach quantifies the resulting damage to human health, measured in Disability-Adjusted Life Years (DALYs). The conversion from midpoint to endpoint indicators is achieved through conversion factors available in literature[47]. We rank using endpoint indicators to align with the paper's overarching theme of technology impacts. While calculating the absolute release of emissions is useful, it does not fully reflect the tangible consequences a technology will have on a person or community.

To achieve this, the endpoint method aggregates the various midpoint indicators into three distinct damage categories: human health, ecosystems, and resource availability. Some midpoint categories feed into a single endpoint, while others contribute to multiple damage pathways. Furthermore, this paper employs the Individualist (I) cultural perspective for endpoint weighting. This perspective focuses on short-term, proven

effects, making it highly suitable for assessing technologies with a rapid rate of future development.

We also present the midpoint indicators for 2070 in Supplementary Table 16.

Supplementary Table 16: Ammonia midpoint indicators for 2070, with emissions allocated

Impact category	Unit	T1	T2	T3 - 2070	T4 - 2070	T5 - 2070	T6 - 2070	T7 - 2070 RN	T8 - 2070 RN
Global warming	kg CO2 eq	3937.48	2593.66	630.35	2135.47	1095.71	644.43	554.16	864.22
Stratospheric ozone depletion	kg CFC11 eq	1.80E-04	2.81E-04	1.31E-04	2.64E-04	2.19E-04	1.28E-04	1.18E-04	2.02E-04
Ionizing radiation	kBq Co-60 eq	13.23	14.98	24.73	11919.43	35.35	20.85	18.03	29.90
Ozone formation, Human health	kg NOx eq	2.71	3.66	2.46	3.69	3.06	1.81	1.59	2.45
Fine particulate matter formation	kg PM2.5 eq	0.19	0.25	0.46	1.11	0.78	0.46	0.39	0.62
Ozone formation, Terrestrial ecosystems	kg NOx eq	3.07	4.15	2.52	3.86	3.17	1.87	1.64	2.53
Terrestrial acidification	kg SO2 eq	2.51	3.15	5.02	5.64	7.47	4.42	3.93	5.96
Freshwater eutrophication	kg P eq	0.13	0.15	0.62	0.57	1.01	0.56	0.55	1.19
Marine eutrophication	kg N eq	0.06	0.07	0.08	0.26	0.07	0.04	0.04	0.11
Terrestrial ecotoxicity	kg 1,4-DCB	4377.58	5374.51	20146.18	8162.12	37313.18	22139.36	18533.19	26970.43
Freshwater ecotoxicity	kg 1,4-DCB	42.06	45.68	849.25	63.21	1641.81	975.31	818.96	1180.05
Marine ecotoxicity	kg 1,4-DCB	18.06	20.82	233.84	27.81	449.17	266.77	223.97	323.39
Human carcinogenic toxicity	kg 1,4-DCB	0.21	0.28	0.73	1.36	1.18	0.70	0.59	0.88
Human non-carcinogenic toxicity	kg 1,4-DCB	21.97	25.07	119.20	101.72	210.91	125.18	107.26	156.94
Land use	m2a crop eq	15.72	18.97	38.22	37.59	59.55	35.29	31.88	48.21
Mineral resource scarcity	kg Cu eq	4.57	4.93	19.62	20.12	32.09	19.05	16.57	24.39
Fossil resource scarcity	kg oil eq	850.42	1138.59	130.19	504.89	226.08	133.01	114.76	179.04
Water consumption	m3	56.81	60.09	62.28	524.96	11.97	8.21	6.84	18.88

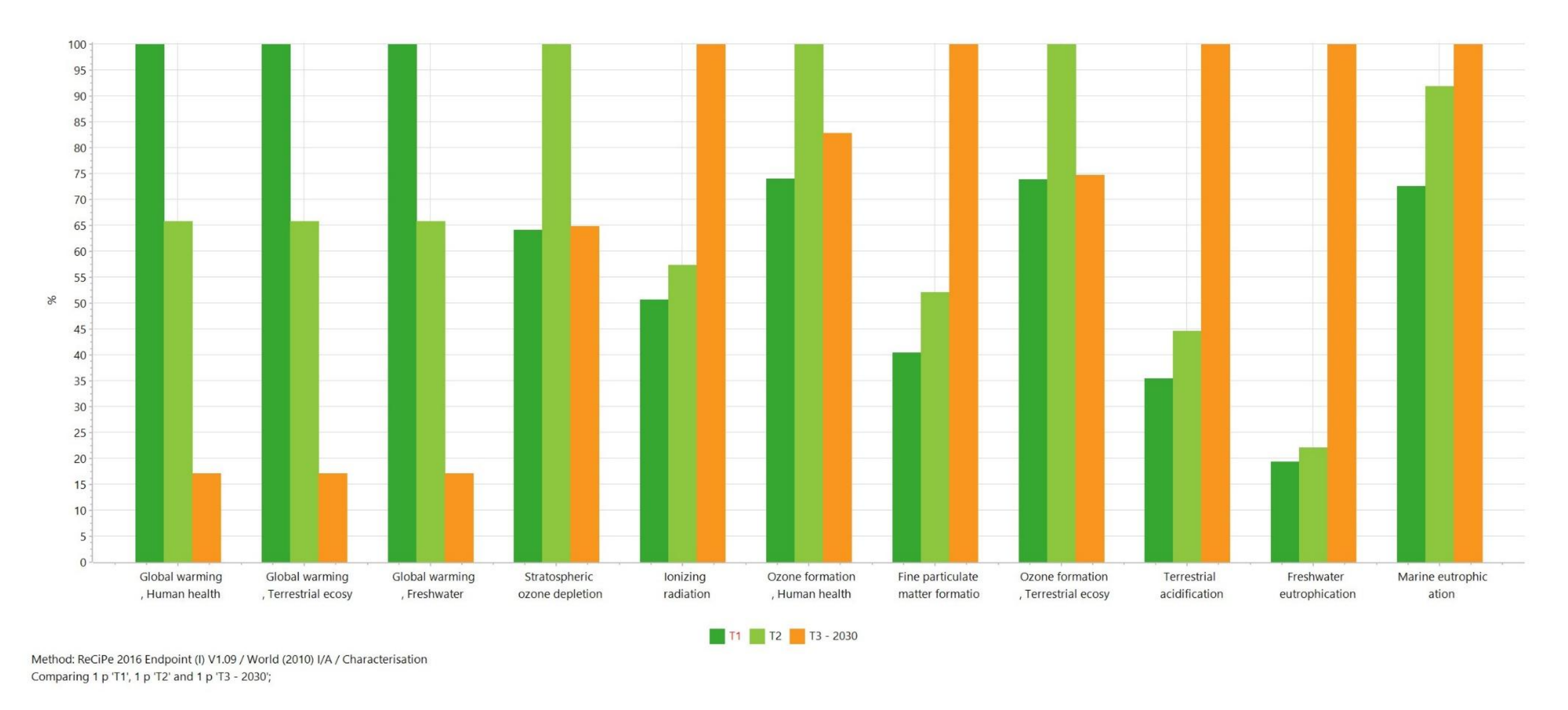

Supplementary Figure 8: 2030 midpoint characterisation, indicators names match left to right those in Supplementary Table 3 up to marine eutrophication.

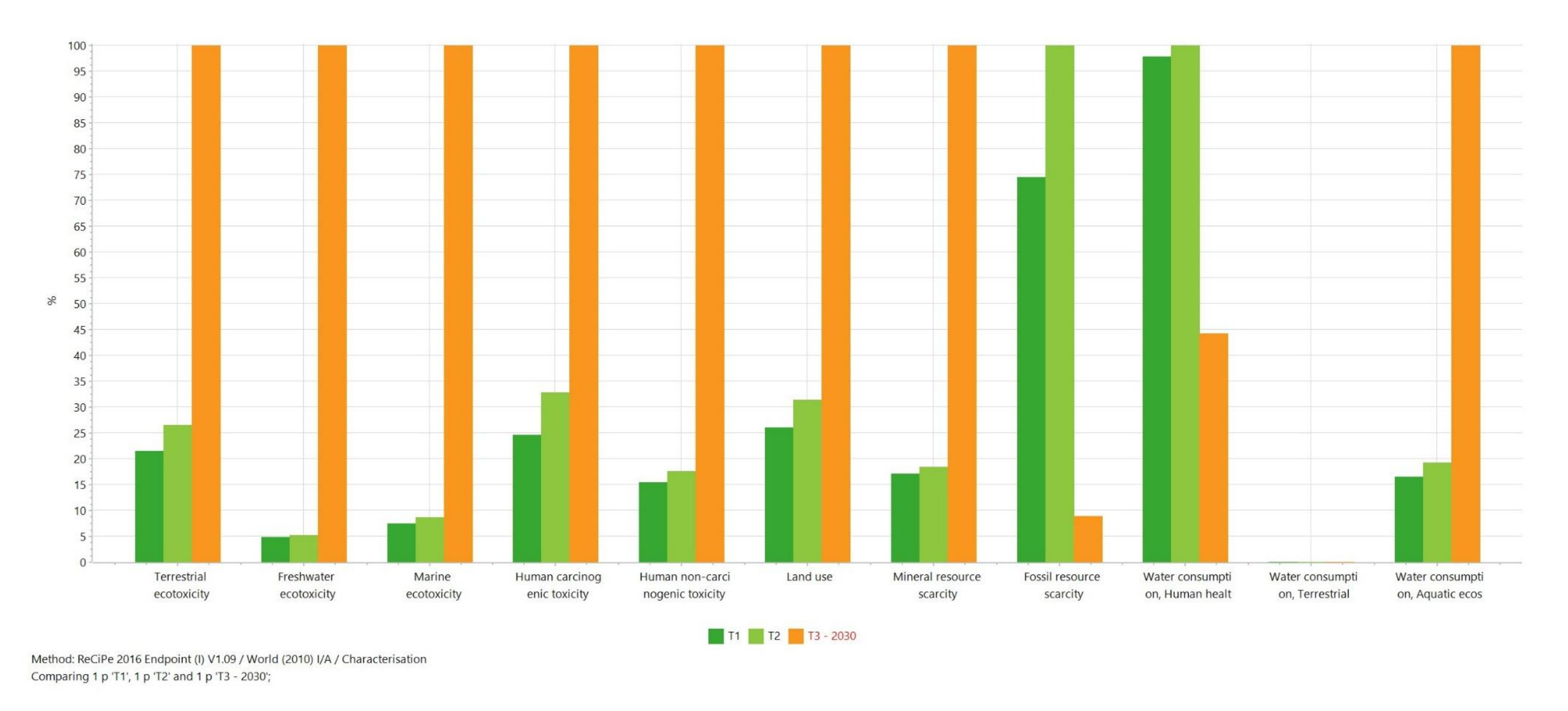

Supplementary Figure 9: 2030 midpoint characterisation, indicators names match left to right those in Supplementary Table 3 from terrestrial ecotoxicity.

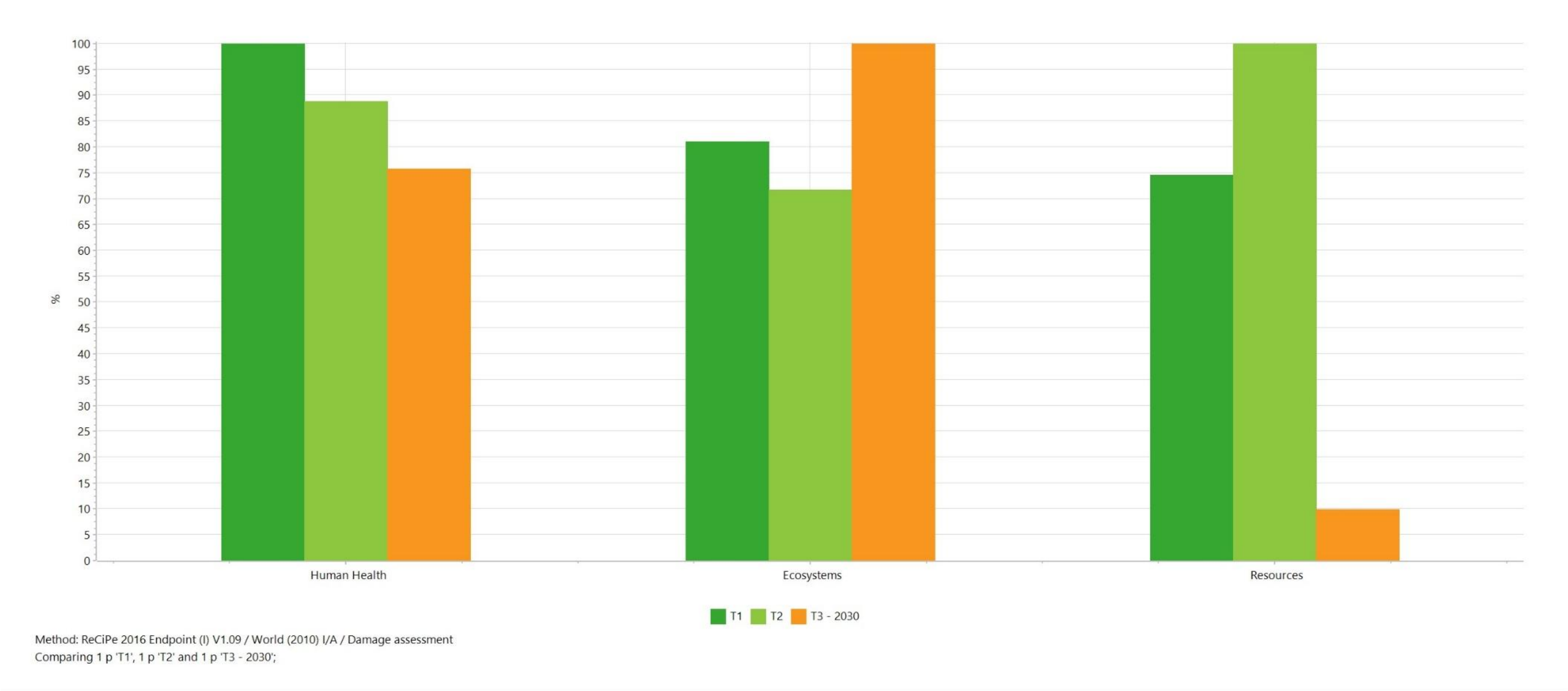

Supplementary Figure 10: 2030 endpoint damage assessment, 100% here represents 0.000619 Disability Adjusted Life Years (DALY), $4.11x10^{-6}$, 405 USD2013, respectively.

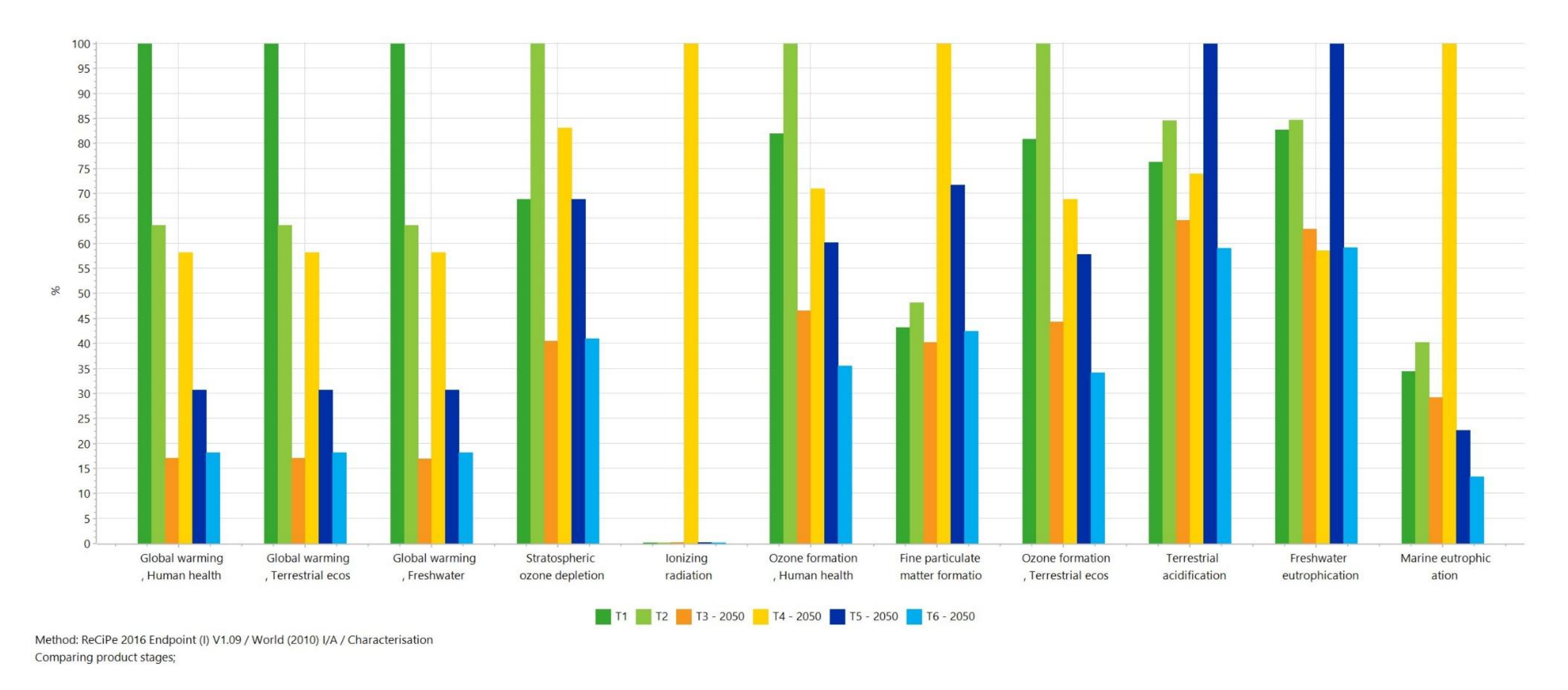

Supplementary Figure 11: 2050 midpoint characterisation, indicators names match left to right those in Supplementary Table 3 up to marine eutrophication.

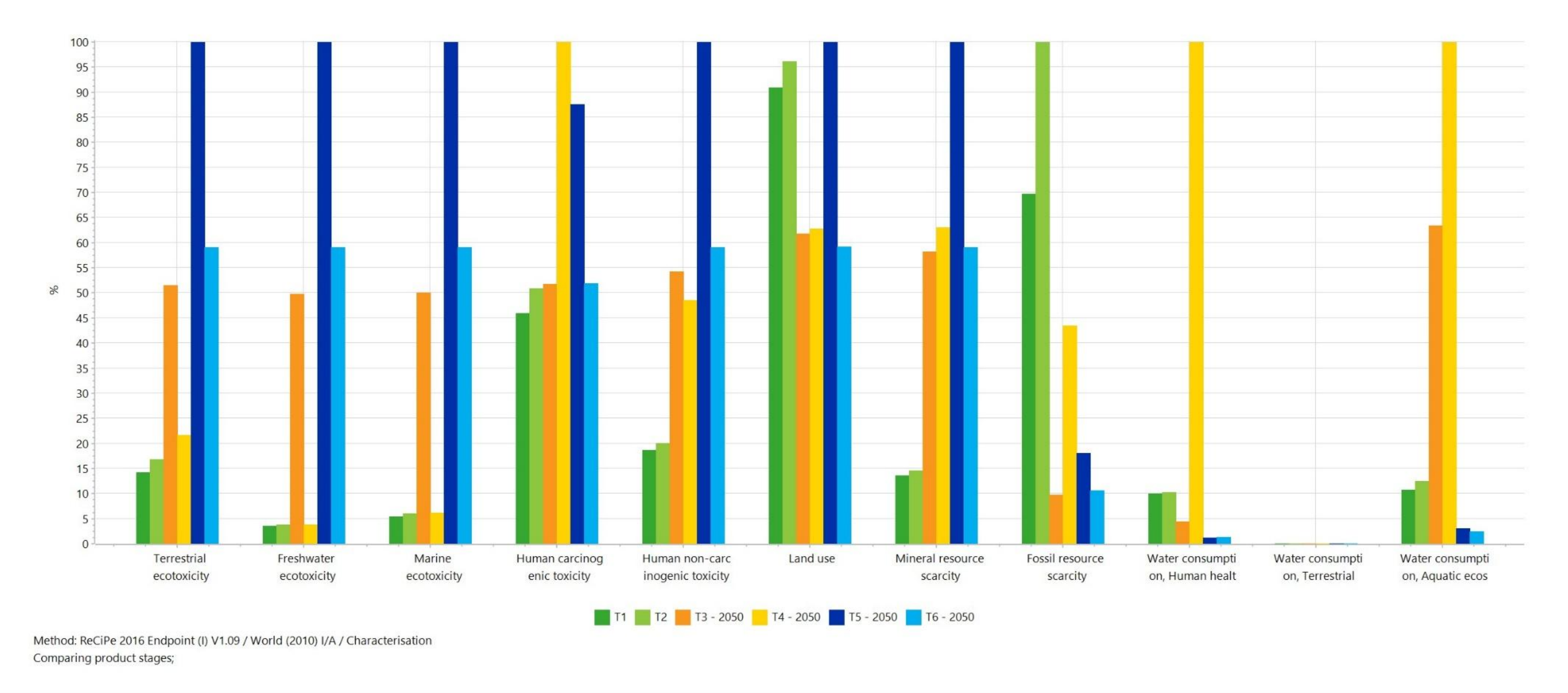

Supplementary Figure 12: 2050 midpoint characterisation, indicators names match left to right those in Supplementary Table 3 from terrestrial ecotoxicity.

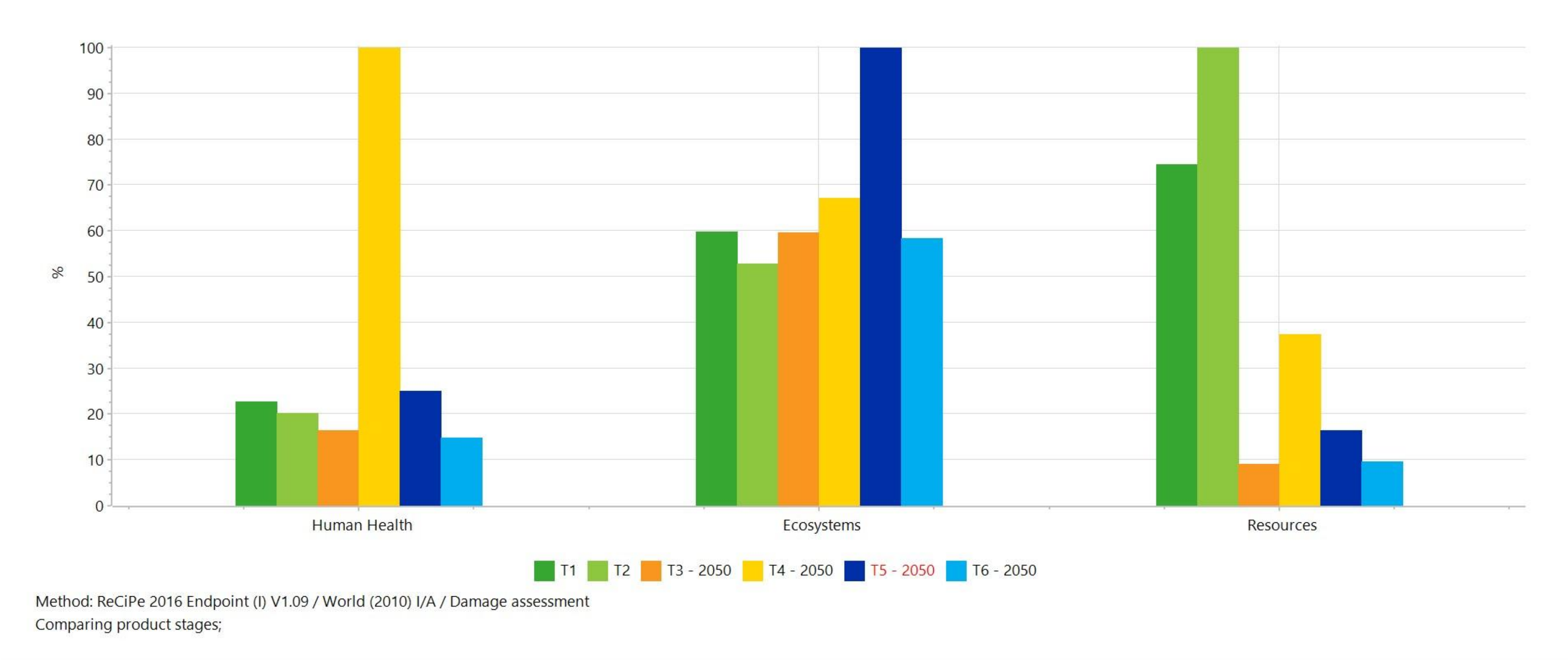

Supplementary Figure 13: 2050 endpoint damage assessment, 100% here represents 0.00271 Disability Adjusted Life Years (DALY), 5.57×10^{-6} species.yr, and 405 USD2013, respectively.

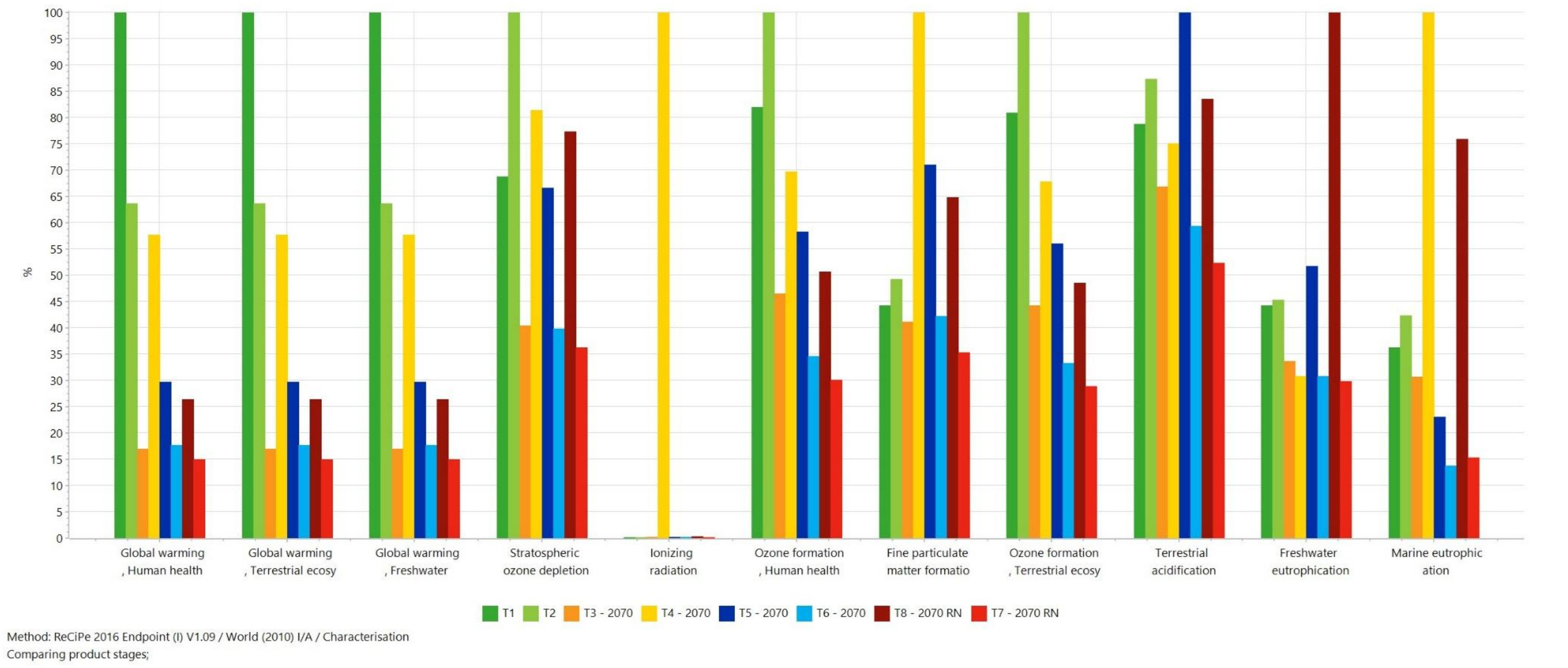

Supplementary Figure 14: 2070 midpoint characterisation, indicators names match left to right those in Supplementary Table 3 up to marine eutrophication.

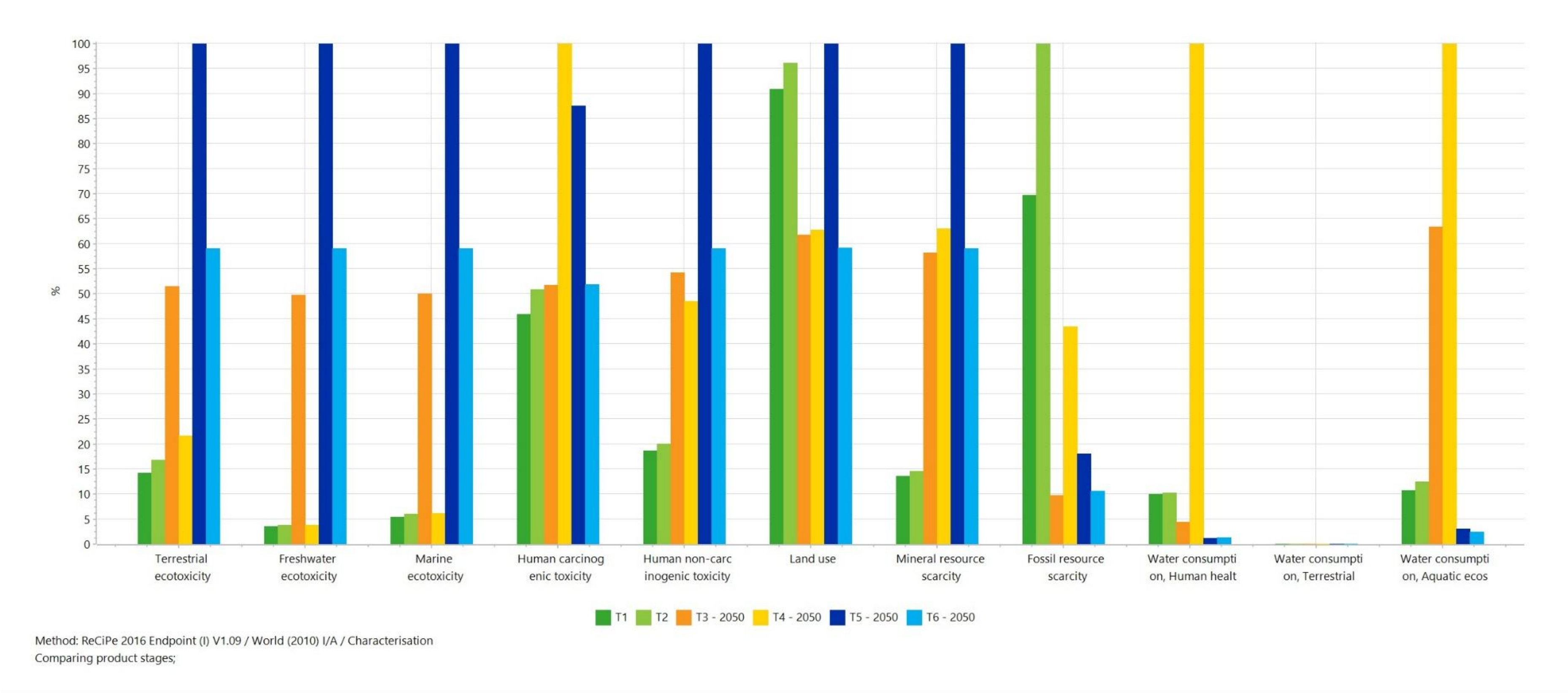

Supplementary Figure 15: 2070 midpoint characterisation, indicators names match left to right those in Supplementary Table 3 from terrestrial ecotoxicity.

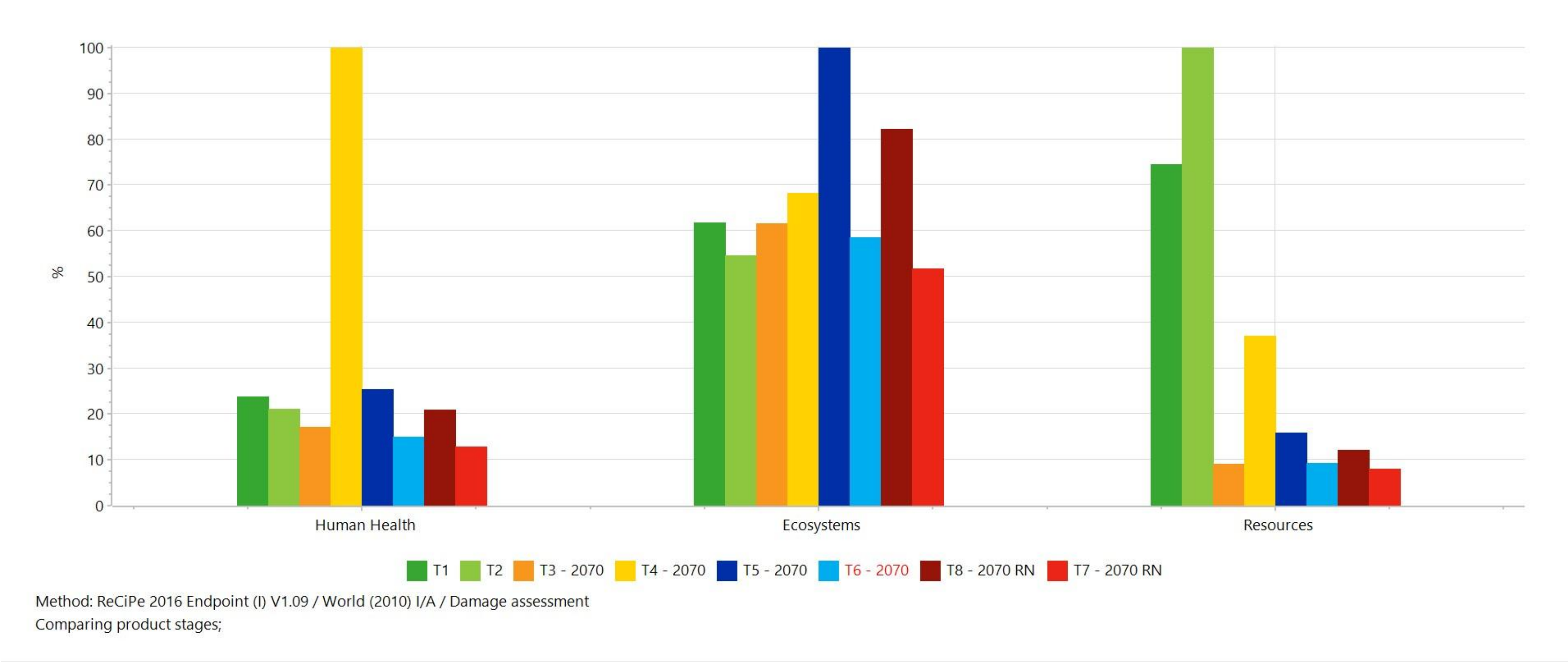

Supplementary Figure 16: 2070 endpoint damage assessment, 100% here represents 0.0026 Disability Adjusted Life Years (DALY), $5.4x10^{-6}$ species.yr, and 405 USD2013, respectively.

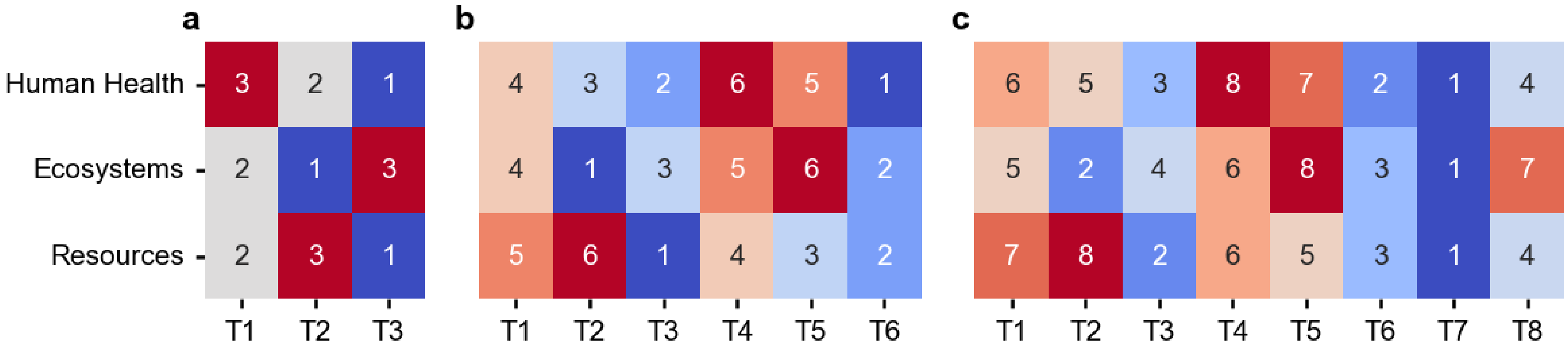

Supplementary Figure 17: Life Cycle Assessment (LCA) indicator rankings for a) 2030 b) 2050 c) 2070

Life Cycle Assessment Hotspot Analysis

We present the hotspot analysis for all eight production routes for the midpoint Global warming potential indicators, in 2070.

Supplementary Table 17: Global Warming Potential (GWP) Hotspot analysis inventory

Inventory Item	T1 (kgCO2eq)	Inventory Item	T2 (kgCO2eq)
Ammonia production	3.94	Ammonia production	3.94
		Carbon Capture	-1.34
Inventory Item	T3 (kgCO2eq)	Inventory Item	T4 (kgCO2eq)
Electricity (wind)	0.30	Nitrogen (separated)	0.530
Green Hydrogen	0.33	SOEC Electrolyser	0.012
		Balance of Plant	0.007
		Electricity (Nuclear)	0.280
		Steam	1.310
Inventory Item	T5 (kgCO2eq)	Inventory Item	T6 (kgCO2eq)
AEM Electrolyser	0.007	Water	0.001
Balance of Plant	0.021	AEM Electrolyser	0.004
Electricity (Wind)	1.070	Balance of Plant	0.012
		Electricity (Wind)	0.637
Inventory Item	T7 (kgCO2eq)	Inventory Item	T8 (kgCO2eq)
PEM Electrolyser	0.004	PEM Electrolyser	0.013
Green Hydrogen	0.334	Water (Ultrapure)	0.081
Electricity (Wind)	0.216	Electricity (Wind)	0.767
		Balance of Plant	0.003

The units of each technology's LCA inventory and their GWP are shown in Supplementary Table 17. For technologies 4-8, the energy utility driving the reaction is the highest emitter of carbon, other than T7 where green hydrogen (and the energy required therein) is highest. T3 – T8 offer substantial emissions savings over the traditionally produced T1 & T2, whose carbon emissions are due to the energy driving the high temperature ammonia production reaction and the steam methane reforming step. Notably for T2, carbon capture, despite offsetting 90% of direct emissions (assumed at 2.25 tonnes of CO_2 per tonne of ammonia) bring life cycle emissions of its own so only reduces total life cycle emissions by 1.34 tonnes (CO_2 equivalent).

Most routes are dominated by one or two stages that account for the majority of emissions, and while this will vary through uncertainty analysis they will continue to account for the majority for each route.

Allocation Data

For electrochemical routes where oxygen is produced as a co-product, T3-T8, we apply economic allocation between ammonia and oxygen, with none given to CO_2 captured in T2 as this is not an economic boon to the process[48,49]. The costs of each, and subsequent allocation of GWP (kgCO2eq) are provided in Supplementary Table 18.

Supplementary Table 18: GWP economic allocation

	T3		T4		T5	
	Cost Fraction	Emissions	Cost Fraction	Emissions	Cost Fraction	Emissions
2030	0.926	625.8	/	/	/	/
2050	0.900	567.5	0.917	1973.8	0.907	1030.8
2060	0.880	554.5	0.906	1934.5	0.904	990.9
	T6		T7		T8	
	Cost Fraction	Emissions	Cost Fraction	Emissions	Cost Fraction	Emissions
2030	/	/	/	/	/	/
2050	0.898	604.1	/	/	/	/
2060	0.823	530.0	0.920	509.8	0.891	770.4

Fundamental Social Life Cycle Assessment results

The fundamental SLCA assessment data is available in Supplementary Data 3 - SLCA spreadsheet**.** The ranking of this data results in Supplementary Figure 18.

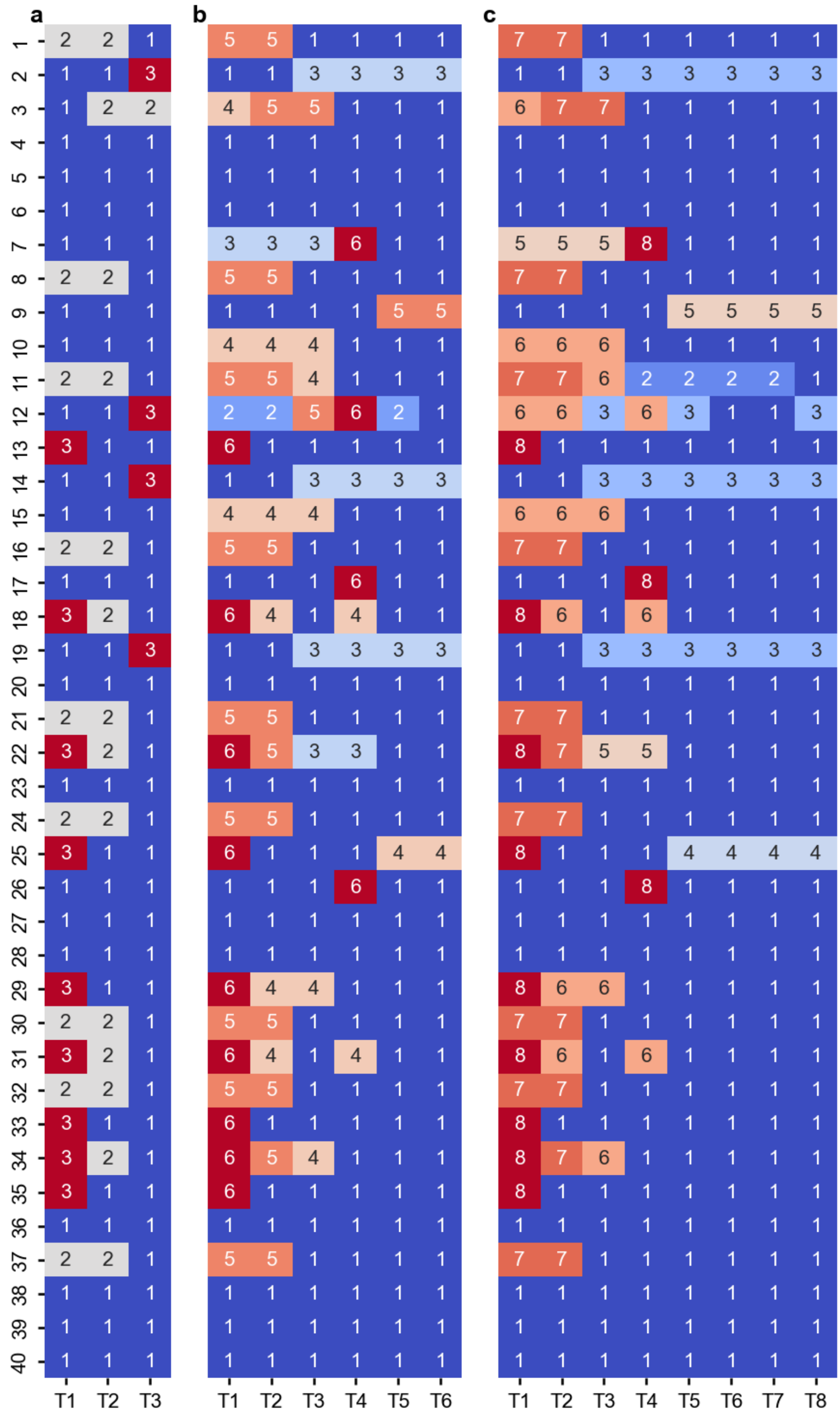

Supplementary Figure 18: Social Life Cycle Assessment (SLCA) indicator rankings for a) 2030 b) 2050 c) 2070

Gompertz and Sigmoidal Learning Curves

The learning rates described in Section 1.2 can be used to generate technological curves to illustrate the development of technological parameters, such as current density. We compare these sigmoidal curves to Gompertz curves for the sake of comparing uncertainty. Supplementary Figure 19 shows the results. T1-T3 do no show current density development curves, as there is no electrochemical effect modelled, instead cost parameters are reflected directly or as performance indexes. T2 & T3 show performance indexes of the blue and green hydrogen, respectively, which form an important part of the ammonia production process, but are not proportionally reflected in the ammonia cost reduction process.

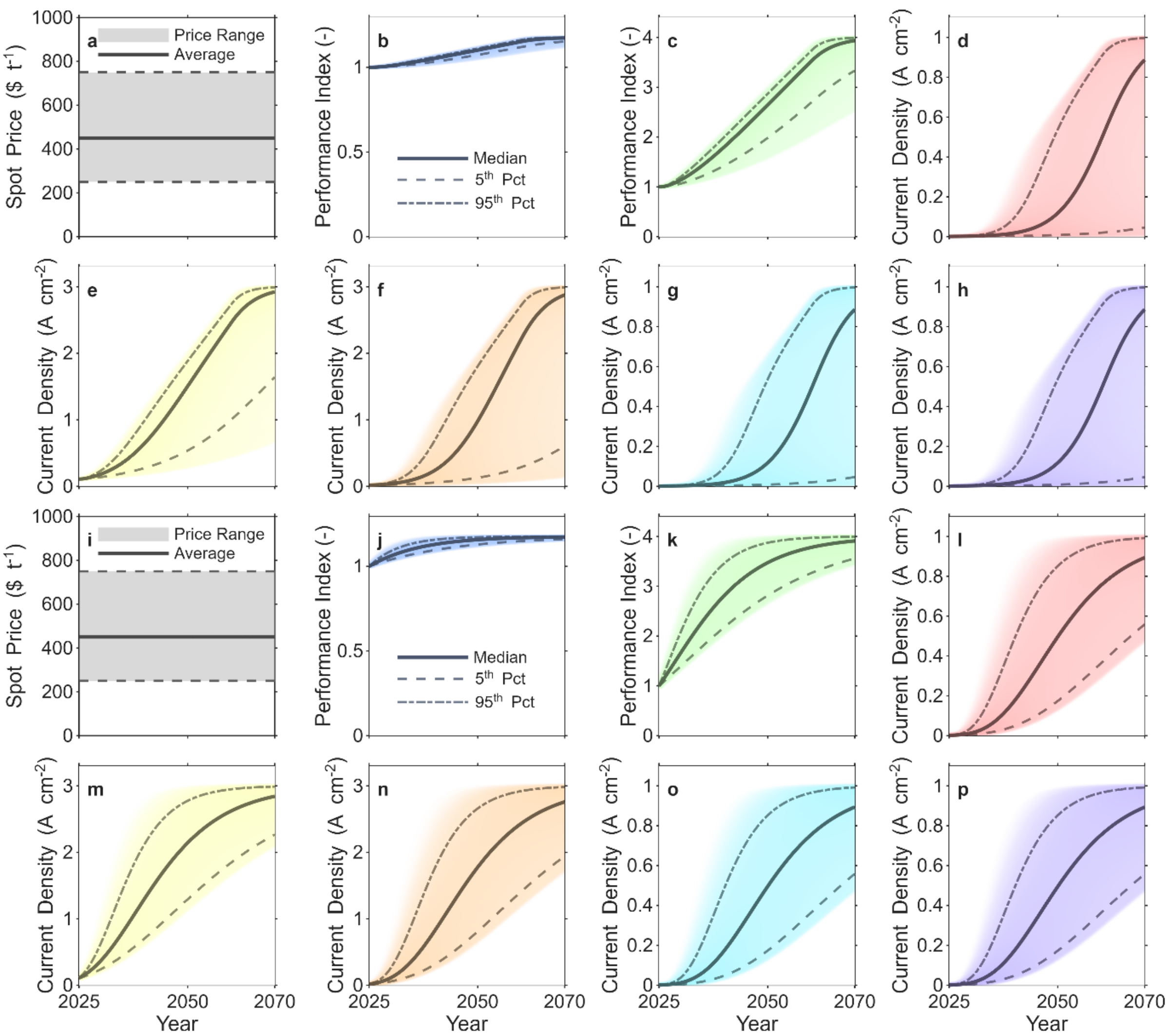

Supplementary Figure 19: Panels a & i show the region of grey ammonia costs. Panels b-h Sigmoidal development curves, Panels j-p Gompertz development curves, for blue to purple technologies, following the colour order described in Supplementary Table 1.

Technology Production Pathways

Supplementary Figure 20 acts to extend the data shown in Figure 3 panels a, d, g, by providing the 0% and 100% growth scenarios for USA, EU, and PRC. The different growth scenarios illustrate clearly the differing final proportions and the degree of plateau each technology achieves by their end date target. This is discussed further in the main text.

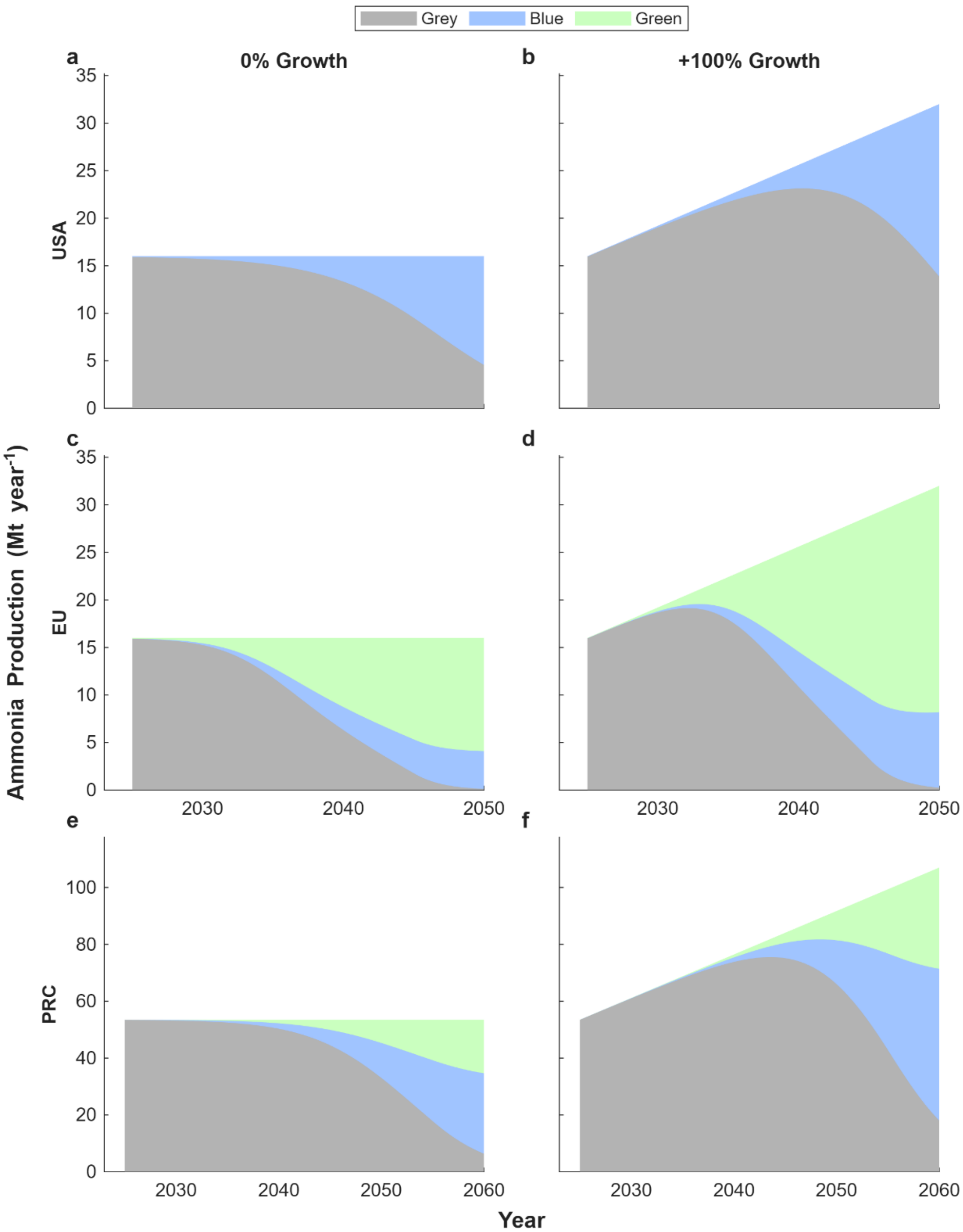

Supplementary Figure 20: Ammonia production scenarios 0% growth (left column) and +100% growth (right column) for USA (a, b), EU (c, d), and PRC, (e, f).

Ranking Uncertainty

Supplementary Figure 21 provides a heatmap of probabilities for technology ranking regions, created by varying the underlying parameters of the LCC, LCA, and SLCA using a Montecarlo simulation (n=10,000). The resultant rankings are converted into a probabilities map for each strand of the LCSA.

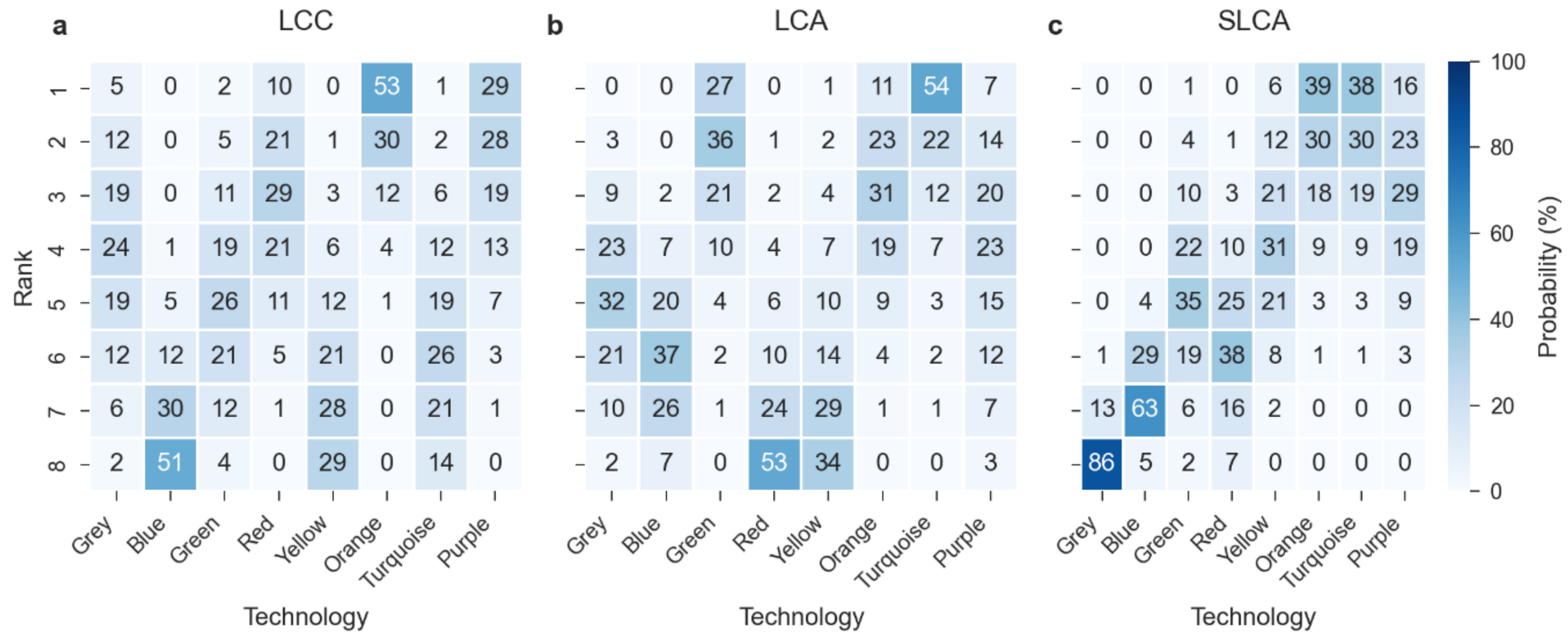

Supplementary Figure 21: Ranking region probabilities for a) life cycle costing (LCC) of allocated emissions b) life cycle assessment (LCA) and c) social LCA (SLCA). Regions of higher probability for a rank are darker and the probability percent that the technology will be ranked there is shown numerically.

Aggregated System Boundary Contributions

To explicitly delineate the environmental burdens attributable to upstream utilities versus the intrinsic ammonia synthesis process, Supplementary Table 19 provides an aggregated summary of the detailed hotspot analysis presented earlier in Supplementary Table 17. This breakdown groups the inventory items into two distinct system boundaries, noting our framework compares technology–utility systems in which the synthesis step's efficiency governs upstream energy requirements, and they together determine each route's sustainability performance and ranking.

Supplementary Table 19: Percentage contribution of upstream utilities versus the intrinsic synthesis loop to Global Warming Potential (GWP).

**Production Pathway**	**Upstream Energy Contributions (Hydrogen & Electricity)**	**Intrinsic synthesis contributions including reaction heat**

**Grey Ammonia (T1)**	Not separatable	Not separatable
**Blue Ammonia (T2)**	Not separatable	Not separatable
**Green Ammonia (T3)**	>99%	<1%
**Red Ammonia (T4)**	39%	61%
**Yellow Ammonia (T5)**	97.4%	2.6%
**Orange Ammonia (T6)**	97.6%	2.4%
**Turquoise Ammonia (T7)**	>99%	<1%
**Purple Ammonia (T8)**	98.2%	1.8%

The distribution of impacts in Table 19 reflects the fundamentally different thermodynamic architectures of these pathways. For the conventional fossil routes (T1 and T2), the process is highly thermally integrated within the LCA databases and physical plant design; the heat of reaction and fossil fuel combustion drive both the steam methane reforming (hydrogen production) and the Haber-Bosch synthesis steps simultaneously. Consequently, the emissions cannot be mathematically decoupled by unit operation, and the gross GWP is entirely allocated to the intrinsic synthesis/heat stage.

Conversely, the electrified routes (T3, T5–T8) successfully decouple hydrogen supply from the synthesis loop. Because the heat of reaction and compression steps are provided electrically, and hydrogen is sourced via electrolysis, 100% of their GWP footprint is inherited from these upstream electrical and hydrogen supplies, leaving the intrinsic synthesis loop with low relative contribution to the gross carbon footprint. Nevertheless, the synthesis technology still plays a critical role: its efficiency determines how much upstream electricity and hydrogen are required per tonne of ammonia, and therefore strongly conditions the overall footprint. T4 (Red Ammonia) represents a hybrid architecture, where impacts are cleanly split between upstream electrical demand and direct high-temperature heat.

Supplementary References

1. Mersch, M. *et al*. A comparative techno-economic assessment of blue, green, and hybrid ammonia production in the United States. *Sustain. Energy Fuels* https://doi.org/10.1039/d3se01421e (2024) doi:10.1039/d3se01421e.

2. Mayer, P. *et al*. Blue and green ammonia production: A techno-economic and life cycle assessment perspective. *iScience* **26**, (2023).

3. Erfani, N., Baharudin, L. & Watson, M. Recent advances and intensifications in Haber-Bosch ammonia synthesis process. *Chemical Engineering and Processing - Process Intensification* vol. 204 Preprint at https://doi.org/10.1016/j.cep.2024.109962 (2024).

4. Odenweller, A., Ueckerdt, F., Nemet, G. F., Jensterle, M. & Luderer, G. Probabilistic feasibility space of scaling up green hydrogen supply. *Nat. Energy* **7**, 854–865 (2022).

5. Gans, J. S., Kearney, M., Scott, E. L. & Stern, S. *Choosing Technology: An Entrepreneurial Strategy Approach*. http://www.nber.org/papers/w27489 (2020).

6. Geroski, P. A. *Models of Technology Diffusion*. *Research Policy* vol. 29 www.elsevier.nlrlocatereconbase (2000).

7. Haas, R., Sayer, M., Ajanovic, A. & Auer, H. Technological learning: Lessons learned on energy technologies. *Wiley Interdisciplinary Reviews: Energy and Environment* vol. 12 Preprint at https://doi.org/10.1002/wene.463 (2023).

8. Reksten, A. H., Thomassen, M. S., Møller-Holst, S. & Sundseth, K. Projecting the future cost of PEM and alkaline water electrolysers; a CAPEX model including electrolyser plant size and technology development. *Int. J. Hydrogen Energy* **47**, 38106–38113 (2022).

9. Wang, B., Li, T., Gong, F., Othman, M. H. D. & Xiao, R. Ammonia as a green energy carrier: Electrochemical synthesis and direct ammonia fuel cell - a comprehensive review. *Fuel Processing Technology* **235**, (2022).

10. Bunea, S., Coppens, M. & Urakawa, A. Electrochemical Conversion of NO to NH3 in a PEM Cell. *ACS Catal.* 11345–11351 (2023) doi:10.1021/acscatal.3c01600.

11. Wang, J. *et al*. Performance and mechanism of ammonia production by electrocatalytic nitrate reduction based on dodecahydro-closo-dodecaborate hybrid. *J. Colloid Interface Sci.* **652**, 945–951 (2023).

12. Allen, J., Panquet, S. & Bastiani, A. Electrochemical Ammonia: Power to Ammonia Ratio and Balance of Plant Requirements for Two Different Electrolysis Approaches. *Frontiers in Chemical Engineering* **3**, (2021).

13. International Renewable Energy Agency. *Green Hydrogen Cost Reduction Scaling up Electrolysers to Meet the 1.5°C Climate Goal*. www.irena.org/publications (2020).

14. Daniel, T., Xing, L., Cai, Q., Liu, L. & Xuan, J. Potential of Progressive and Disruptive Innovation-Driven Cost Reductions of Green Hydrogen Production. *Energy & Fuels* https://doi.org/10.1021/ACS.ENERGYFUELS.4C01247 (2024) doi:10.1021/ACS.ENERGYFUELS.4C01247.

15. National Institute for Public Health and the Environment. *ReCiPe 2016 v1.1 A Harmonized Life Cycle Impact Assessment Method at Midpoint and Endpoint Level Report I: Characterization*. www.rivm.nl/en (2017).

16. Traverso, M. *et al. Methodological Sheets for Subcategories in Social Life Cycle Assessment (S-LCA) 2021*. (2021).

17. Behzadian, M., Khanmohammadi Otaghsara, S., Yazdani, M. & Ignatius, J. A state-of the-art survey of TOPSIS applications. *Expert Systems with Applications* vol. 39 13051–13069 Preprint at https://doi.org/10.1016/j.eswa.2012.05.056 (2012).

18. BSI. *ISO 14040: Environmental Management-Life Cycle Assessment-Principles and Framework*. (2006).

19. Madanchian, M. & Taherdoost, H. A comprehensive guide to the TOPSIS method for multi-criteria decision making. *Sustainable Social Development* **1**, (2023).

20. Coulson, J. M., Richardson, J. F., Backhurst, J. R. & Harker, J. H. *Coulson and Richardson's Chemical Engineering Volume 6: Chemical Engineering Design*. (2005).

21. European Commission. REPowerEU Plan. (2022).

22. European Commission. *A Strategic Long-Term Vision for a Prosperous, Modern, Competitive and Climate-Neutral EU Economy*. (2018).

23. JRC135099_01.

24. IEA. CCUS Projects Database. Preprint at https://www.iea.org/data-and-statistics/data-product/ccus-projects-database (2025).

25. United States Department of State. *The Long-Term Strategy of the United States, Pathways to Net-Zero Greenhouse Gas Emissions by 2050*. (2021).

26. Official Journal of the European Union. *REGULATION (EU) 2024/1735 OF THE EUROPEAN PARLIAMENT AND OF THE COUNCIL*. (2024).

27. Espitalier-Noël, M. *et al. CLEAN HYDROGEN MONITOR*. (2024).

28. IEA. *Renewables 2024*. www.iea.org (2024).

29. *The Value and Opportunities for Deployment A REPORT FROM THE OIL AND GAS CLIMATE INITIATIVE*. (2021).

30. Zhao, T. & van Dorest, B. *The Competitive Edge of China's Electrolysers*. https://www.woodmac.com/news/opinion/the-competitive-edge-of-chinas-electrolysers/ (2024).

31. Ting Li Wei Liu, A. *et al. China's Green Hydrogen New Era: A 2030 Renewable Hydrogen 100 GW Roadmap Report*. https://rmi.org/insight/chinas-green- (2022).

32. Zhao, F., Bai, F., Liu, X. & Liu, Z. A Review on Renewable Energy Transition under China's Carbon Neutrality Target. *Sustainability (Switzerland)* vol. 14 Preprint at https://doi.org/10.3390/su142215006 (2022).

33. Lan, R., Irvine, J. T. S. & Tao, S. Synthesis of ammonia directly from air and water at ambient temperature and pressure. *Sci. Rep.* **3**, (2013).

34. Bareiß, K., de la Rua, C., Möckl, M. & Hamacher, T. Life cycle assessment of hydrogen from proton exchange membrane water electrolysis in future energy systems. *Appl. Energy* **237**, 862–872 (2019).

35. Flavell-While, C. Fritz Haber and Carl Bosch – Feed the World. *The Chemical Engineer* (2010).

36. Demirhan, C. D., Tso, W. W., Powell, J. B. & Pistikopoulos, E. N. Sustainable ammonia production through process synthesis and global optimization. *AIChE Journal* **65**, (2019).

37. Lee, K. *et al.* Techno-economic performances and life cycle greenhouse gas emissions of various ammonia production pathways including conventional, carbon-capturing, nuclear-powered, and renewable production. *Green Chemistry* **24**, 4830–4844 (2022).

38. Singh, B., Strømman, A. H. & Hertwich, E. G. Comparative life cycle environmental assessment of CCS technologies. *International Journal of Greenhouse Gas Control* **5**, 911–921 (2011).

39. Energy Transitions Commission. *Making the Hydrogen Economy Possible: Accelerating Clean Hydrogen in an Electrified Economy The Making Mission Possible Series*. www.linkedin.com/company/energy-transitionscommission (2021).

40. Park, S., Shin, Y., Jeong, E. & Han, M. Techno-economic analysis of green and blue hybrid processes for ammonia production. *Korean Journal of Chemical Engineering* **40**, 2657–2670 (2023).

41. Giddey, S., Badwal, S. P. S. & Kulkarni, A. Review of electrochemical ammonia production technologies and materials. *Int. J. Hydrogen Energy* **38**, 14576–14594 (2013).

42. Chen, W. *et al.* Emerging Applications, Developments, Prospects, and Challenges of Electrochemical Nitrate-to-Ammonia Conversion. *Adv. Funct. Mater.* **33**, (2023).

43. Glibert, P. M., Maranger, R., Sobota, D. J. & Bouwman, L. The Haber Bosch-harmful algal bloom (HB-HAB) link. *Environmental Research Letters* **9**, (2014).

44. Shao, J. *et al.* Electrochemical synthesis of ammonia from nitric oxide using a copper–tin alloy catalyst. *Nat. Energy* **8**, 1273–1283 (2023).

45. Long, J. *et al.* Direct Electrochemical Ammonia Synthesis from Nitric Oxide. *Angew. Chem. Int. Ed.* **59**, 9711–9718 (2020).

46. Santhosh, C. R. & Sankannavar, R. A comprehensive review on electrochemical green ammonia synthesis: From conventional to distinctive strategies for efficient nitrogen fixation. *Appl. Energy* **352**, (2023).

47. Huijbregts, M. A. J. *et al. ReCiPe 2016 v1.1 A Harmonized Life Cycle Impact Assessment Method at Midpoint and Endpoint Level Report I: Characterization*. www.rivm.nl/en (2017).

48. Ardente, F. & Cellura, M. Economic Allocation in Life Cycle Assessment: The State of the Art and Discussion of Examples. *J. Ind. Ecol.* **16**, 387–398 (2012).

49. Bauer, C. *et al.* On the climate impacts of blue hydrogen production. *Sustain. Energy Fuels* **6**, 66–75 (2022).
