# Supplementary material for: Ammonia at the net-zero crossroads
Source: Nat Commun. 2026 Jul 21;17:8902. doi: 10.1038/s41467-026-75880-2 (PMC13503741; doi:10.1038/s41467-026-75880-2)
Supplement: Supplementary file 2 [file 41467_2026_75880_MOESM2_ESM.pdf]

Description of Additional Supplementary Files

[Note - Supplementary Data 1–4 have been uploaded to Figshare and can be accessed via the DOI or Figshare link]

File Name - Supplementary Data 1

Description - An excel spreadsheet containing the LCA inventories.

File Name - Supplementary Data 2

Description - A zipped folder containing the raw HYSYS files.

File Name - Supplementary Data 3

Description - An excel spreadsheet containing the raw SLCA scoring.

File Name - Supplementary Data 4

Description - An excel spreadsheet containing the source data for Figure 4.

* DOI LINK -

https://doi.org/10.6084/m9.figshare.32172879

(The DOI link will be active and accessible upon publication of the paper.)

* Figshare Link -

https://figshare.com/s/8356124ef4d95e423335
