# Supplementary material for: Ammonia at the net-zero crossroads
Source: Nat Commun. 2026 Jul 21;17:8902. doi: 10.1038/s41467-026-75880-2 (PMC13503741; doi:10.1038/s41467-026-75880-2)
Supplement: Supplementary file 3 [file 41467_2026_75880_MOESM3_ESM.pdf]

nature portfolio

Peer Review File

Ammonia at the Net-Zero Crossroads

Corresponding Author: Professor Jin Xuan

This file contains all reviewer reports in order by version, followed by all author rebuttals in order by version.

Version 0:

Reviewer comments:

Reviewer #1

(Remarks to the Author)
This is an important, timely, and refreshingly realistic contribution to the ammonia decarbonisation debate. At a moment when much of the literature and policy discourse is driven by optimistic projections and technological enthusiasm, the authors provide something far more valuable: a systems-level, physics-grounded, and evidence-based assessment of what is actually feasible within mid-century timelines.

The central conclusion, that net-zero ammonia by 2050 is not achievable under a doubling of global demand, is both sobering and necessary. Rather than reinforcing prevailing narratives, the authors confront the uncomfortable reality that infrastructure constraints, lifecycle emissions, cost burdens, and technology maturity timelines fundamentally limit what can be delivered by 2050.

The introduction of the “Ammonia Rainbow” framework represents a meaningful methodological advance. By integrating process-level modelling, learning curve projections, full life-cycle assessment (LCA), techno-economic analysis (TEA), and social LCA, the authors move decisively beyond static taxonomies and fragmented comparisons . The predictive, probabilistic approach to technology maturation is particularly commendable, as it replaces speculation with structured forecasting.

Importantly, the article resists the temptation to overstate the promise of electrochemical routes. The treatment of “purple ammonia” is especially balanced: while acknowledging its long-term disruptive potential, the authors clearly demonstrate that without step-change breakthroughs in current density, voltage, and electricity cost, commercial relevance before 2070 is unlikely.

Equally valuable is the systems perspective. The quantification of green hydrogen, CCS, and renewable electricity requirements exposes hidden infrastructure competition across sectors. Demonstrating that ammonia could consume up to 45% of green hydrogen supply in certain scenarios forces a broader policy conversation that extends beyond sectoral silos. This is precisely the type of analysis that responsible energy planning requires.

The paper’s most courageous contribution, however, lies in challenging the assumption of unchecked ammonia demand growth. By showing that demand moderation materially narrows the net-zero gap, the authors shift the debate from purely technological supply-side solutions to societal choices about fertiliser efficiency, circular nitrogen management, and prioritised energy applications. This demand-side framing aligns with a growing recognition in the sustainability literature that decarbonisation is not merely an engineering optimisation problem.

In summary, this article stands out because it refuses hype. It does not promise silver bullets. It does not assume perfect policy alignment or infinite renewable resources. Instead, it provides a rigorous, transparent, and sober assessment of technological readiness, infrastructure constraints, and economic trade-offs. In doing so, it makes a crucial contribution to evidence-based policymaking and responsible innovation in the ammonia sector.

Realism in climate transition analysis is not pessimism.

Reviewer #2

(Remarks to the Author)

Manuscript states that all eight technologies were modelled in Aspen HYSYS but the models are not described in detail especially reaction kinetics to ensure reproducibility. Also, simplified stoichiometric models are used emerging electrochemical routes (T5–T8). A traceable inventory table for all routes (feedstock assumptions, reactor conversions, energy balances) and justification for the selection of electrochemical performance parameters is required.
The technological maturity predictions for 2030, 2050, 2070 rely heavily on sigmoidal learning curve and assumed k values for all routes, however, no empirical data exist for most electrochemical ammonia routes to justify these learning rates. Monte Carlo simulation (n=10,000) does not completely address structural uncertainty in technological evolution.
In LCA methodology, the endpoint scoring is compressing impacts into a single metric resulting into misleading rankings and endpoint methods are not recommended for emerging technologies with high uncertainty. LCA inventories are incomplete (e.g., balance of plant materials, catalyst requirements, electrolyzer stack lifetimes). Blue ammonia and electrochemical pathways implicitly involve co-products and manuscript does not clearly state the allocation method used. Hotspot analysis will also be required.

Reviewer #3

(Remarks to the Author)
1 The concept of the ammonia rainbow proposed in this manuscript is highly interesting. However, several methodological aspects require further clarification. Ammonia production can generally be classified into thermochemical and electrochemical pathways. For both emissions and cost assessments, the results are strongly influenced by the definition of system boundaries and the choice of utilities, including: the use of fossil-based versus renewable feedstocks (e.g., biomass), the source of electricity (100% renewable, grid electricity, or nuclear), and the source of steam (internal waste heat recovery versus external natural gas supply). These assumptions can substantially affect the reported emissions and costs. It is therefore unclear how a fair comparison among different ammonia production pathways is ensured.
2. In particular, it would be helpful to clarify whether all pathways should be evaluated under harmonized assumptions for electricity and steam supply. For example, SOEC-based routes appear to rely on nuclear electricity, whereas other pathways are based on green electricity, which may introduce inconsistencies in the comparison. Furthermore, within the “green” scenario, it would be worth discussing whether biomass-based hydrogen production could be considered, as this option may better align with certain definitions of “green” ammonia. Overall, different ammonia production pathways involve distinct technology choices and system configurations. Additional discussion is needed to explain how these differences are treated and how comparability across pathways is maintained to ensure a fair and transparent assessment.
3 In Figure 1b, the life-cycle assessment (LCA) results show that, in 2030, the blue scenario outperforms the grey scenario due to the inclusion of CCUS, which is reasonable. However, emissions continue to decline substantially in 2050 and 2070, eventually falling below those of the grey scenario. The underlying reasons for this trend are unclear and would benefit from further explanation.
4 Similarly, in Figure 1c, the blue scenario is shown to be more expensive than the green scenario in 2030, and its relative ranking continues to decline over time, eventually becoming the most expensive option. According to several reports and studies, blue hydrogen is generally considered more cost-competitive than green hydrogen at present and is often viewed as a short- to medium-term solution when cost is taken into account. Additional clarification on the assumptions driving these cost projections would therefore be helpful.
5 Appendix Section 3 is poorly structured and difficult to interpret. The appendix primarily presents rankings rather than absolute numerical results, particularly for the cost analysis. Under the defined system boundaries and KPIs, it would be more informative to report the absolute cost of producing 1 kg of ammonia, together with a detailed cost breakdown (e.g., CAPEX, OPEX, electricity, feedstock, CCUS-related costs) and a clear identification of the dominant cost drivers. In addition, given the inherent uncertainties, reporting a cost range rather than a single point estimate would strengthen the analysis.
6 For the LCA results, the current figures are not sufficiently clear. It is recommended to focus on the endpoint indicators, clearly explain how different impact categories are weighted and aggregated, and improve the readability of the figures. Moreover, it is unclear whether global warming potential (GWP) has been included in the final results; if not, this indicator should be explicitly reported, given its central relevance.
7 Regarding the results shown in Figure 2, it is unclear whether the assumed demand trends for the three scenarios are supported by existing literature. If demand growth is set to 0%, 50%, and 100%, the resulting differences mainly reflect changes in slope, which limits the attractiveness and interpretability of the results. Please clarify the basis for assuming linear demand growth and whether this assumption is grounded in published projections or reports.
8 The direct and indirect emissions trends appear nearly identical. The authors are encouraged to better summarize and interpret the key insights from Figure 2 and to consider focusing on more meaningful or representative results rather than presenting multiple curves with similar trends. Additionally, it is not clearly stated how emissions are distributed among the gray, blue, and green pathways in this analysis. The relative shares of these pathways are expected to differ significantly across regions such as Europe, the United States, and China, yet this regional heterogeneity is not reflected or distinguished in the presented results. Further clarification or differentiation would improve the robustness of the conclusions.
9 The title and caption of Figure 3 lack essential explanations, making the figure difficult to interpret. For example, it is unclear why the United States is excluded from panels (a) and (c), and which country or region is represented in panels (d) and (e). These points should be clearly stated to avoid ambiguity.
10 Panels (a–c) show the required shares of green hydrogen, CCS, and renewable energy, yet the assumptions underlying the projected capacities in 2030 and 2050 are not clearly explained. Please clarify: whether these capacity growth trajectories are based on literature, policy targets, or scenario assumptions; and how deviations from these assumed growth rates would affect the results. In particular, if the deployment of green hydrogen, CCS, or renewables falls short of expectations, emissions are likely to remain high. A discussion of this sensitivity, especially for emissions, would strengthen the analysis.
11 Several cost-related assumptions require clarification. It appears that the cost of grey hydrogen is assumed to remain constant, while the cost of green hydrogen is assumed to decline primarily due to falling electricity prices and electrolyzer

costs. If so, it is unclear why the costs of other pathways that also rely on electrolysis do not largely decrease and are cheaper than grey scenario. Figure 3 would benefit from additional analysis and explanation to clarify these assumptions and their implications. Based on the results shown in Figure 3, it would also be helpful to explicitly summarize which pathway(s) are suggested to be the most promising options for the future.

12 In the Discussion section, the manuscript emphasizes that achieving net-zero emissions in the ammonia sector is strongly linked to the availability of green hydrogen. However, because the environmental impacts of the ammonia synthesis process itself appear to be much smaller than those associated with hydrogen production and electricity/heat supply, it remains unclear how the future role of ammonia synthesis should be interpreted. Does this imply that decarbonizing ammonia production mainly depends on selecting appropriate green hydrogen pathways and ensuring sufficient cost reductions in hydrogen supply?

13 At present, the largest barrier to green hydrogen deployment is cost. Approximately 99% of hydrogen on the market is still produced from fossil fuels, with only a small fraction coming from green or blue hydrogen. Consequently, downstream ammonia production is inevitably constrained by the availability and cost of low-carbon hydrogen. The manuscript would benefit from a clearer discussion of how the ammonia industry could be incentivized or enabled to adopt green hydrogen, given these upstream limitations.

14 If hydrogen sourcing is treated as an external constraint, it is unclear what mitigation potential remains within the ammonia synthesis stage itself. Please clarify what technological or operational improvements at the ammonia synthesis level (e.g., process efficiency, heat integration, electrification, or alternative synthesis routes) could meaningfully contribute to emissions reduction, independent of changes in hydrogen supply.

15 In SI, several parameter assumptions and their temporal evolution require clearer justification. For example, parameters such as CD and FE are assumed to change over time, whereas lifetime is kept constant. While it is reasonable to assume that performance metrics improve with technology maturation, the manuscript does not provide sufficient evidence or references to support the assumed time-dependent trajectories. For electrolysis in particular, technology learning would also be expected to increase stack/system lifetime. In this context, the assumption of 40,000 hours in 2030 appears potentially aggressive and should be justified with credible sources and/or sensitivity ranges.

16 It does not clearly account for technology learning-by-doing and economies of scale. If demand increases and deployment scales up, key cost parameters (e.g., T1, T2, T3, and other cost coefficients) would likely evolve over time rather than remain fixed. Please clarify whether such learning effects are considered; if not, discuss how excluding them may bias the cost trajectories and scenario comparisons.

17 From an LCA perspective, the SI currently lacks several essential elements. Most importantly, the system boundary and functional unit are not clearly stated. In addition, the LCIA framework (impact categories/method and characterization approach) is not sufficiently described, and inventory-level details are missing: the inputs/outputs of each unit process should be transparent to enable interpretation and reproducibility. The assumptions in Table S6 appear insufficiently supported; the authors should provide references, rationale, or uncertainty ranges for these values.

Reviewer #4

(Remarks to the Author)

1) The final sentence of the first paragraph in the introduction is: “This puts us at a crucial crossroads where decisions on which ammonia route to follow are being taken.” It would be beneficial to the reader to provide more clarification on ammonia route. Is this to mean “production route”? Perhaps this could be re-written as “This puts us at a crucial crossroads where decisions on the way forward for ammonia production and utilization are being taken.”

2) The language for the “green ammonia” classification in Box 1 should be more precise. In this production scenario, Haber-Bosch is not powered by green hydrogen but rather fed with green hydrogen. Additionally, have any constraints been placed on the carbon intensity of the energy source used to power water electrolysis in the green hydrogen scenario? Perhaps the authors could consider including some additional detail about the energy source for green hydrogen production in the green ammonia scenario.

3) The TEA and LCA for green ammonia production assume constant hydrogen availability (per my understanding of the Supporting Information). How is this reconciled with the variable nature of renewable energy likely to be used to power water electrolysis for green hydrogen production? Specifically, how is this variability buffered and what effect does this have on TEA and LCA results?

4) It would be more informative to the reader if the vertical axes titles in Figure 1b-d could be written as “LCA rank”, “LCC rank”, and “SCLA rank” respectively rather than simply “rank” for each subfigure. Also, please be consistent with the use of the terms “levelized cost” and “life cycle” cost as it pertains to the meaning of the abbreviation LCC.

5) Why is a longer time horizon (2060 vs. 2050) considered for the PRC’s ammonia transition relative the U.S. and the EU?

6) Perhaps the authors could comment on what causes the emissions intensity of grey, blue, and green ammonia to be higher than the direct emissions reported in literature. What are the biggest contributors to the indirect emissions for each ammonia production pathway?

7) Is the 3:1 ratio of green/blue ammonia deployment assumed for both the U.S. and the EU? If so, please reconcile this with the previous statements that U.S. policy favors blue ammonia production whereas EU policy favors green ammonia. If not, please better clarify these ratios for both the U.S. and EU as has been done for the PRC in the original manuscript.

Version 1:

Reviewer comments:

Reviewer #2

(Remarks to the Author)
The authors have meaningfully and efficiently addressed my comments/suggestion and the quality of the manuscript has been significantly improved. I appreciate the efforts of the authors in revising the manuscript and clarifying the raised concerns during the review process. In its current form, the manuscript is much clearer, more rigorous, and suitable for publication.

Reviewer #3

(Remarks to the Author)
Thank the authors for their detailed responses to the reviewer comments and for the substantial revisions made to the manuscript. The revised version has improved significantly. In particular, the additional explanations regarding the LCA/LCC framework, uncertainty treatment, and system-level constraints are appreciated. However, several important methodological and interpretational issues still require further clarification and discussion in the main text before the manuscript can be considered fully resolved.

1. The revised manuscript now explicitly states that the feasibility and cost of low-carbon ammonia are controlled primarily by hydrogen and energy supply systems before 2050. This raises an important question regarding what is fundamentally being compared in the present work: (i) intrinsic ammonia synthesis technologies, or (ii) broader hydrogen production system configurations coupled to ammonia production. At present, it remains difficult to distinguish the contribution of the ammonia synthesis stage itself from the contribution of upstream processes.
The manuscript would therefore benefit from a clearer separation between: (i) impacts attributable to hydrogen/electricity supply systems, and (ii) impacts attributable to the ammonia synthesis technologies themselves.
At minimum, the authors should discuss this limitation more explicitly and clarify the extent to which the current framework should be interpreted as a “hydrogen-energy system comparison” rather than an intrinsic ammonia synthesis technology comparison.

2. Although the revised manuscript now clarifies the rationale behind differentiated utility assumptions, comparability across pathways remains somewhat difficult to interpret because the environmental and economic outcomes are still strongly coupled to the assumed electricity and heat sources. Even if a full harmonized re-analysis is outside the scope of the current study, the authors should discuss more explicitly how strongly the current rankings depend on upstream utility choices and how this may affect interpretation of the comparative results. This is particularly important for interpreting the relative performance of blue, green, and red ammonia pathways.

3. The revised manuscript provides a clearer explanation of the observed rank inversions and the role of the multi-criteria framework. However, the updated discussion also suggests that several conclusions are highly sensitive to the relative ranking methodology. This raises an important methodological concern regarding the robustness and interpretability of the ranking-based conclusions, particularly for emerging technologies with highly uncertain inventories and performance assumptions.
For several pathway comparisons, the absolute differences in environmental or economic performance appear relatively modest, whereas the ranking changes are comparatively large. The manuscript would therefore benefit from a more explicit discussion of: (i) the dependence of conclusions on the chosen ranking framework; (ii) the potential instability of rankings under different weighting assumptions; and (iii) the distinction between absolute performance changes and relative ranking shifts.
This clarification would help readers better interpret whether the reported technology transitions reflect fundamental performance differences or whether they are primarily driven by the behaviour of the multi-criteria ranking methodology.

Reviewer #4

(Remarks to the Author)
The authors have addressed the reviewers' comments in a comprehensive way. In my view, this paper is suitable for publication.

Version 2:

Reviewer comments:

Reviewer #3

(Remarks to the Author)
I would like to sincerely thank the authors for their detailed responses and the substantial effort invested in addressing the reviewer comments. The explanations provided are clear, comprehensive, and very helpful in clarifying the concerns raised

during the review process. I highly appreciate the authors' careful work and constructive revisions.

Open Access This Peer Review File is licensed under a Creative Commons Attribution 4.0 International License, which permits use, sharing, adaptation, distribution and reproduction in any medium or format, as long as you give appropriate credit to the original author(s) and the source, provide a link to the Creative Commons license, and indicate if changes were made.
In cases where reviewers are anonymous, credit should be given to 'Anonymous Referee' and the source.
The images or other third party material in this Peer Review File are included in the article's Creative Commons license, unless indicated otherwise in a credit line to the material. If material is not included in the article's Creative Commons license and your intended use is not permitted by statutory regulation or exceeds the permitted use, you will need to obtain permission directly from the copyright holder.
To view a copy of this license, visit https://creativecommons.org/licenses/by/4.0/

Reviewer #1 (Remarks to the Author):

This is an important, timely, and refreshingly realistic contribution to the ammonia decarbonisation debate. At a moment when much of the literature and policy discourse is driven by optimistic projections and technological enthusiasm, the authors provide something far more valuable: a systems-level, physics-grounded, and evidence-based assessment of what is actually feasible within mid-century timelines.

The central conclusion, that net-zero ammonia by 2050 is not achievable under a doubling of global demand, is both sobering and necessary. Rather than reinforcing prevailing narratives, the authors confront the uncomfortable reality that infrastructure constraints, lifecycle emissions, cost burdens, and technology maturity timelines fundamentally limit what can be delivered by 2050.

The introduction of the “Ammonia Rainbow” framework represents a meaningful methodological advance. By integrating process-level modelling, learning curve projections, full life-cycle assessment (LCA), techno-economic analysis (TEA), and social LCA, the authors move decisively beyond static taxonomies and fragmented comparisons ￼. The predictive, probabilistic approach to technology maturation is particularly commendable, as it replaces speculation with structured forecasting.

Importantly, the article resists the temptation to overstate the promise of electrochemical routes. The treatment of “purple ammonia” is especially balanced: while acknowledging its long-term disruptive potential, the authors clearly demonstrate that without step-change breakthroughs in current density, voltage, and electricity cost, commercial relevance before 2070 is unlikely.

Equally valuable is the systems perspective. The quantification of green hydrogen, CCS, and renewable electricity requirements exposes hidden infrastructure competition across sectors. Demonstrating that ammonia could consume up to 45% of green hydrogen supply in certain scenarios forces a broader policy conversation that extends beyond sectoral silos. This is precisely the type of analysis that responsible energy planning requires.

The paper’s most courageous contribution, however, lies in challenging the assumption of unchecked ammonia demand growth. By showing that demand moderation materially narrows the net-zero gap, the authors shift the debate from purely technological supply-side solutions to societal choices about fertiliser efficiency, circular nitrogen management, and prioritised energy applications. This demand-side framing aligns with a growing recognition in the sustainability literature that decarbonisation is not merely an engineering optimisation problem.

In summary, this article stands out because it refuses hype. It does not promise silver bullets. It does not assume perfect policy alignment or infinite renewable resources. Instead, it provides a rigorous, transparent, and sober assessment of technological readiness, infrastructure constraints, and economic trade-offs. In doing so, it makes a crucial contribution to evidence-based policymaking and responsible innovation in the ammonia sector.

Realism in climate transition analysis is not pessimism.

Author Response: We sincerely thank the reviewer for their careful and thorough evaluation of our manuscript, and for their highly encouraging feedback. We are thrilled that the reviewer found our systems-level approach, the "Ammonia Rainbow" framework, and our realistic assessment of technological and infrastructure constraints to be valuable contributions to the field. We deeply appreciate the recognition of our efforts to provide a sober, evidence-based perspective on the ammonia decarbonization debate.

Reviewer #2 (Remarks to the Author):

Manuscript states that all eight technologies were modelled in Aspen HYSYS but the models are not described in detail especially reaction kinetics to ensure reproducibility. Also, simplified stoichiometric models are used emerging electrochemical routes (T5–T8). A traceable inventory table for all routes (feedstock assumptions, reactor conversions, energy balances) and justification for the selection of electrochemical performance parameters is required.

Author Response: An inventory table has been added to section S1.1 in the supplementary information. Electrochemical performance parameters are presented in section S1.2 with literature sources provided in text.

The technological maturity predictions for 2030, 2050, 2070 rely heavily on sigmoidal learning curve and assumed k values for all routes, however, no empirical data exist for most electrochemical ammonia routes to justify these learning rates.

Monte Carlo simulation (n=10,000) does not completely address structural uncertainty in technological evolution.

Author Response:

We are grateful for this comment, and we recognise that it connects directly to the editor's concern regarding **uncertainty quantification** and the **clarity of our methods**. We fully agree that empirical learning data for most electrochemical ammonia routes are very limited, and that simply applying Monte Carlo sampling around a fixed functional form does not, on its own, resolve structural uncertainty. In revision we have therefore made three sets of changes:

(1) Clarifying and justifying the learning-curve methodology.
We now describe our technological learning framework more explicitly in both the main Methods and SI Section 1.2 ("Technological Advancement"). We explain that the sigmoidal formulation is adopted as a stylised representation of technology maturation, consistent with the approach of Odenweller et al. (Nature Energy 2022) and a large literature on energy technology diffusion, and that it is applied to performance metrics (current density and faradaic efficiency) rather than directly to costs. Because empirical learning data for electrochemical ammonia do not yet exist, we calibrate the **range** of *k* and the asymptotic performance limits by analogy with better-documented electrochemical systems (PEM water electrolysers and related devices; refs 8, 18, 20, 36). We now report the numerical ranges of *k* used in the Monte Carlo (truncated normal distribution with lower bound 5% and standard deviation 8%) and show that the resulting 2050 performance levels for PEM-like systems are in line with independent projections for electrolysers.

(2) Making uncertainty quantification more explicit and comprehensive.
We have added a new Fig 2 with discussions and the corresponding Methods text to

provide a clearer, step-by-step description of how uncertainty is treated in each strand of our Life Cycle Sustainability Assessment:

- **Techno-economic analysis:** in addition to uncertainty in electrochemical parameters and learning rates, we now describe explicitly how CAPEX, OPEX, and LCOA are perturbed (up to ±50%) and propagated through a Monte Carlo simulation with n=10,000 to obtain distributions of levelised costs and rankings.
- **LCA:** we clarify that we use the uncertainty information embedded in the Ecoinvent database and perform Monte Carlo with n=10,000 for midpoint and endpoint indicators, deriving not only point estimates but also ranking distributions.
- **SLCA:** we reiterate that each technology–indicator score is allowed to vary by ±1 on the 1–5 scale in the Monte Carlo, and we now explicitly show how this propagates into distributions of SLCA rankings. For each strand we now report summary statistics (median and interquartile range of rank) in the revised figures and in a new supplementary table, making the **extent of uncertainty visible**, rather than only the central ranks.

(3) Adding dedicated analyses of *structural* uncertainty in technological evolution.
To address the reviewer's specific point that our original Monte Carlo did not fully capture structural uncertainty in learning, we have added a new subsection in the SI S1.2.1 & S2.5 . In these sections we go beyond parameter variation and test:

- **Wider ranges of learning parameters:** for immature electrochemical routes we alter the mean *k* values by up to 5 percentage points to reflect potential fundamental constraints. For each of these structurally different learning assumptions we recompute the key outcomes: (i) the time windows in which each route reaches techno-economic viability, (ii) the LCOA distributions for 2050 and 2070, and (iii) the possibility of achieving net-zero alignment by 2050 given system-level CCS, hydrogen, and renewable electricity constraints. Across all scenarios we find that:
    - the **relative hierarchy** of grey, blue, and green ammonia is preserved;
    - waste-to-ammonia routes (T5–T6) only become competitive after mid-century; and
    - direct nitrogen-reduction routes (T7–T8) do not become cost-competitive before 2070 unless the disruptive step changes of Tables S8–S9 are invoked.

      These results demonstrate that our main **systems-level conclusions are robust** to both parameter and structural uncertainty in technological learning: they are controlled primarily by infrastructure

bottlenecks and physical limits, rather than by any single assumed value of *k*.

- **Alternative functional forms** for technology evolution:
  – a comparison between the sigmoidal logistic and Gompertz alternative with the new sentence added:
  - "A similar observation applies to the comparison between the sigmoidal and Gompertz logistic curves; whilst they vary at specific timepoints (particularly around 2050), they generally show a similar upwards development trend, with the Gompertz curves being less constrained than the sigmoidal ones."

Finally, to avoid overstating the precision of our projections, we have revised the main text (Results, "The Ammonia Rainbow" and Methods, "Future Projections and Scenarios") to state explicitly that the 2030, 2050, and 2070 technology values should be interpreted as **internally consistent scenario projections**, not deterministic forecasts. We believe these additions directly address the referees' and editor's concerns about uncertainty quantification and methodological clarity.

In LCA methodology, the endpoint scoring is compressing impacts into a single metric resulting into misleading rankings and endpoint methods are not recommended for emerging technologies with high uncertainty.

Author Response:

We agree that endpoint indicators add an extra layer of modelling and that compressing impacts can obscure trade-offs, which is especially relevant for emerging technologies. In line with the reviewer's request to improve **uncertainty quantification** and **methods clarity**, we have made three focused changes:

(1) Clarified the role and limitations of endpoints.
In the main Methods and revised SI Section 2.3 we now state explicitly that ReCiPe 2016 Endpoint (Individualist) is used primarily to aggregate multiple midpoint indicators into the three damage categories (Human Health, Ecosystems, Resources) for integration within the LCSA framework. We add a clear caveat that endpoint results for immature technologies carry higher uncertainty and should be interpreted as **relative, screening-level indicators**, not precise absolute impacts.

(2) Elevated midpoint information and checked for ranking bias.
We now improve normalised midpoint profiles for key technologies in SI (Figs S8&9, S11&12, S14&15), and discussed these results in the results section for figure 1. The midpoint-based rankings are consistent with the endpoint-based rankings within the Monte Carlo rank uncertainty, and the relative positions of grey, blue, green, and electrochemical routes are unchanged. This reduces the risk that endpoint compression is driving misleading overall rankings.

(3) Made endpoint uncertainty explicit.

SI Section 1.8 now clarifies that Monte Carlo simulations (n=10,000) are run at the **midpoint level** using the Ecoinvent uncertainty data, and that endpoint scores inherit this variability. We provide summary statistics (median and interquartile range) for both midpoint- and endpoint-based ranks in a new supplementary table, so the extent of LCA uncertainty is transparent.

These changes retain the integrative value of endpoint indicators for the LCSA, while making trade-offs visible at the midpoint level and demonstrating that our key environmental conclusions do not depend on endpoint aggregation alone.

LCA inventories are incomplete (e.g., balance of plant materials, catalyst requirements, electrolyzer stack lifetimes).

Author Response: We have updated the inventories Excel spreadsheet to improve completeness and transparency. This spreadsheet is available in the Figshare folder.

Blue ammonia and electrochemical pathways implicitly involve co-products and manuscript does not clearly state the allocation method used.

Author Response:

We thank the reviewer for pointing this out and agree that our co-product treatment should be clearly specified. Allocation method is references 48 & 49 in the SI.

(1) Allocation for captured CO_2 **(blue ammonia).**
For blue ammonia routes, we **do not allocate any environmental burdens away from ammonia to the captured** CO_2 **stream**. Captured CO_2 is modelled as a stored waste stream with no credit for potential utilisation or substitution of other products. All upstream burdens of capture, compression, and storage are therefore fully assigned to ammonia. We have now stated this explicitly in the LCA Methods (main text) and in SI Section 2.3.2.

(2) Allocation for oxygen from electrochemical pathways.
For electrochemical routes where oxygen is produced as a co-product (e.g. PEM water electrolysis supplying green hydrogen), we apply **economic allocation** between ammonia and oxygen:

- the total burdens of the electrolyser stage are partitioned according to the **economic value shares of ammonia and oxygen**;
- the prices used for this allocation are taken from market data reported in the techno-economic analysis (oxygen sell price in Table S2) and the assumed ammonia selling price at minimum-feasibility LCOA.
  This approach is now described explicitly in the Methods and detailed in Table

S18 in the SI, so that the allocation ratios and underlying prices are transparent.

(3) Sensitivity and impact on conclusions.
We have additionally checked a sensitivity case where **no allocation is applied to oxygen** (i.e. all burdens assigned to ammonia). The resulting changes in LCA scores and rankings are minor and lie within the Monte Carlo uncertainty ranges already reported, and they do not alter our qualitative conclusions on the relative performance of blue, green, and electrochemical routes.

We believe these clarifications address the reviewer's concern by making our co-product assumptions explicit and demonstrating that our key findings are not dependent on a particular allocation choice.

Hotspot analysis will also be required.

Author Response:

We agree that hotspot analysis is important to identify which life-cycle stages and processes dominate the impacts, and this also strengthens the transparency of our methods.

(1) Addition of a dedicated hotspot analysis.
We have now carried out a **contribution (hotspot) analysis** for the main technologies at 2070 using the ReCiPe midpoint indicators in SimaPro. For each route, where possible, we decompose total impacts into:

- upstream utility supply (electricity, hydrogen production),
- core process unit operations (electrolysers, reactors, CCS), and
- downstream balance of plant.

The results are presented in a new subsection in the SI (Section S2.3.1 "Hotspot analysis").

(2) Use of hotspots to interpret rankings.
In the main text (Results / Discussion) we now briefly reference these hotspots to explain *why* specific routes rank as they do. For example:

- "As shown in Table S17, green ammonia's emissions are almost evenly split into the green hydrogen production step and the ammonia production step."
- "Red ammonia has good cost performance but is poor in other categories mainly due to its association with nuclear energy."

This links the LCA rankings to concrete process drivers and indicates where mitigation or technological improvement would have the largest effect.

(3) Alignment with uncertainty analysis.
We also note in SI Section 2.3.1 that the hotspot patterns are robust across the Monte Carlo simulations:

"Most routes are dominated by one or two stages that account for the majority of emissions, and while this will vary through uncertainty analysis they will continue to account for the majority for each route"

This supports the robustness of the hotspot insights under the quantified uncertainty.

These additions provide the requested hotspot analysis and further clarify the mechanisms behind the environmental performance of each pathway.

Reviewer #3 (Remarks to the Author):

1 The concept of the ammonia rainbow proposed in this manuscript is highly interesting. However, several methodological aspects require further clarification. Ammonia production can generally be classified into thermochemical and electrochemical pathways. For both emissions and cost assessments, the results are strongly influenced by the definition of system boundaries and the choice of utilities, including: the use of fossil-based versus renewable feedstocks (e.g., biomass), the source of electricity (100% renewable, grid electricity, or nuclear), and the source of steam (internal waste heat recovery versus external natural gas supply). These assumptions can substantially affect the reported emissions and costs. It is therefore unclear how a fair comparison among different ammonia production pathways is ensured.

Author Response:

We agree that utility assumptions are critical for a fair comparison, and we apologise that this was not sufficiently explicit. Our intention is to compare technologies under **internally consistent and deliberately differentiated utility choices** that reflect their real-world roles:

(1) Utility choices and fairness of comparison.
We now clarify in the Methods and SI 2.1 that:

- **Grey and blue ammonia** are defined to represent **current real-world practice**, where both heat and electricity are **carbon intensive**. Grey and blue therefore use fossil-based steam and globally representative grid electricity, consistent with existing industrial plants.
- **Green ammonia** assumes an **electrified Haber–Bosch process** with hydrogen from **renewable electrolysis**; process electricity is renewably supplied, and heat demand is met from this electrified configuration.

- **Red ammonia (high-temperature electrochemical)** assumes heat and electricity from a **high-temperature industrial source**, here represented by **nuclear**, which simultaneously supplies process heat and power.
- The remaining **low-temperature electrochemical technologies** (yellow, orange, turquoise, purple) are modelled with **renewable wind electricity** as their power source.

In all cases, grid electricity mixes are taken from Ecoinvent; where “grid” is used, we rely on the database’s **weighted global average**, while “renewable” is consistently modelled as a weight global wind power average across all low-temperature processes. This ensures that technologies intended to be “low-carbon” are compared under the same renewable supply conditions, while grey/blue reflect the carbon-intensive utilities they currently rely on.

(2) Role of utility differences in the comparison.
These utility differences are **not an artefact** but an intentional part of the comparison: they, together with process efficiencies and material requirements, are what distinguish grey, blue, green, red and the electrochemical routes. We make this explicit in Table S1 that lists, for each technology, the electricity source, heat/steam source, and boundary notes.

(3) Scope regarding biomass.
We have also clarified that **biomass-based electricity or biomass feedstocks are not considered** in the present study. Including biomass would constitute an additional, distinct pathway which could be incorporated in future as the “ammonia rainbow” evolves, in SI 1.1 and 2.1, in analogy with the expanding “hydrogen rainbow”. Excluding biomass here keeps the comparison focused on the thermochemical and electrochemical routes currently most discussed in policy and industry.

With these clarifications and the added SI table, we aim to make the system boundaries and utility assumptions fully transparent, and to show how they are applied consistently so that the comparison between ammonia production pathways is fair and interpretable.

2. In particular, it would be helpful to clarify whether all pathways should be evaluated under harmonized assumptions for electricity and steam supply. For example, SOEC-based routes appear to rely on nuclear electricity, whereas other pathways are based on green electricity, which may introduce inconsistencies in the comparison. Furthermore, within the “green” scenario, it would be worth discussing whether biomass-based hydrogen production could be considered, as this option may better align with certain definitions of “green” ammonia. Overall, different ammonia production pathways involve distinct technology choices and system configurations. Additional discussion is needed to explain how these differences are

treated and how comparability across pathways is maintained to ensure a fair and transparent assessment.

Author Response:.

We agree that the assumptions on electricity and steam supply, and on what constitutes "green", must be clearly articulated to ensure a fair and transparent comparison.

(1) Harmonisation versus deliberate differentiation of utilities.
We now clarify in the Methods and SI that we use **two layers** of assumptions:

- A **harmonised baseline** for *low-temperature* "green / electrochemical" routes: all such pathways (green Haber–Bosch and yellow, orange, turquoise, purple) use **renewable wind electricity** as their power source, and steam/low-grade heat is provided by this low-carbon electricity (via electrified utilities) where needed.
- **Deliberate differentiation** where the route definition intrinsically implies a specific utility:
  - **Grey/blue**: grid electricity and fossil-based steam, representing current practice.
  - **Red (SOEC-based)**: high-temperature heat and electricity from **nuclear**, reflecting the concept of a symbiotic nuclear–electrolysis system (Table S1).

Thus, all "green" low-temperature pathways are treated under harmonised renewable assumptions, while "grey/blue" and "red" are intentionally modelled with the utility context that defines them technologically. We now summarise this explicitly in Table S1 and reference it from the Methods. This makes clear where assumptions are harmonised and where differences are intrinsic to the route's definition.

(2) Rationale for nuclear electricity in SOEC routes.
The nuclear assumption for SOEC (red) is not arbitrary but reflects published concepts where SOECs are thermally integrated with high-temperature nuclear or similar industrial heat sources. We now make this rationale explicit in SI Section 2.1 and in Table S1.

(3) Scope of "green" ammonia and biomass-based hydrogen.
We appreciate the suggestion to consider **biomass-based hydrogen** under a broader "green" definition. In this work we intentionally restrict "green" to **renewable-electricity-based hydrogen** to keep the comparison focused and because biomass-based routes introduce additional uncertainties (land use, biogenic carbon accounting, region-specific sustainability) that would require a dedicated framework. We now state this explicitly in the Table 1 and in SI Section 1.1, and we

note that biomass-based ammonia would be a valuable **future extension of the ammonia rainbow**, analogous to the way the hydrogen rainbow has expanded.

(4) How comparability is maintained despite different configurations.
Finally, we have added a short paragraph in the Methods explaining that:

- all pathways share a **common system boundary (cradle-to-plant-gate)**;
- within that boundary, utility assumptions are either harmonised (all low-temperature “green” routes) or explicitly tied to the defining technology concept (grey/blue fossil, red nuclear); and
- differences in utilities, process efficiencies, and materials are treated as **intrinsic features** of each option, not as modelling artefacts.
  This, together with the new SI table and a brief sensitivity note for SOEC electricity sourcing, clarifies how we preserve **comparability** while still reflecting genuine technological distinctions.

We believe these clarifications directly address the concern about harmonisation, make our “green” definitions explicit, and show how comparability across pathways is maintained in a fair and transparent way.

3 In Figure 1b, the life-cycle assessment (LCA) results show that, in 2030, the blue scenario outperforms the grey scenario due to the inclusion of CCUS, which is reasonable. However, emissions continue to decline substantially in 2050 and 2070, eventually falling below those of the grey scenario. The underlying reasons for this trend are unclear and would benefit from further explanation.

Author Response:

We thank the reviewer for highlighting this trend, as it indicates a need for clearer distinction in the text between absolute environmental metrics and our multi-criteria ranking results. The shift observed in 2050 and 2070 is not due to the absolute emissions of blue ammonia becoming worse than grey but is instead an artefact of the ranking methodology itself, known as a 'rank inversion'.

To address your comment and clarify the underlying reasons for this change, we have expanded our explanation of both the absolute values and the ranking mechanics:

- **Absolute Values vs. Ranking**: In absolute terms, the underlying technologies for grey and blue ammonia do not fundamentally change their relationship; blue consistently maintains lower emissions due to CCUS, whilst grey maintains lower resource use. However, our methodology ranks these technologies by measuring their distance from a theoretical 'ideal' performance across all metrics.

- **The 2030 Baseline**: In 2030, blue ammonia ranks higher than grey. Against the 2030 'ideal', the significant emissions savings provided by CCUS outweigh the penalty of its higher resource consumption, bringing it closer to the optimal benchmark.
- **The 2050/2070 Rank Inversion**: In 2050, the introduction of novel, highly sustainable technologies (such as green ammonia) radically shifts the parameters of the theoretical 'ideal'. When assessed against this new, much more stringent benchmark, the heavy resource penalty associated with blue ammonia causes it to be penalised more harshly than grey. This results in blue dropping below grey in the relative rankings, despite their absolute technical parameters remaining stable.

Furthermore, we have added Section 1.7 to the Supplementary Information to provide a detailed, mathematical explanation of the ranking mechanics and the potential for rank inversion as new technologies enter the model. To ensure this is clear to readers, we have added the below statement there:

- "Initially, blue ammonia is ranked above grey for LCA; however, it performs significantly worse than grey regarding resource use. In 2050, the addition of new, higher-performing technologies fundamentally shifts the theoretical ideal. This shifts the weighting of the distance-to-target metric, causing a rank inversion between blue and grey within the ranking methodology [25]."

4 Similarly, in Figure 1c, the blue scenario is shown to be more expensive than the green scenario in 2030, and its relative ranking continues to decline over time, eventually becoming the most expensive option. According to several reports and studies, blue hydrogen is generally considered more cost-competitive than green hydrogen at present and is often viewed as a short- to medium-term solution when cost is taken into account. Additional clarification on the assumptions driving these cost projections would therefore be helpful.

Author Response: Thank you for highlighting this. We have updated our economic modelling, and blue ammonia is now correctly ranked above green ammonia in the initial timeframes.

Upon review, we identified and corrected a less-appropriate cost assumptions that was artificially boosting the economic viability of green ammonia. This has now been rectified, placing the life cycle cost (LCC) of green ammonia appropriately below that of blue ammonia initially.

As now clarified in SI 2.1, while the cost of blue ammonia does fall slightly over time, the cost of green ammonia declines at a much faster rate. This accelerated cost reduction, combined with the introduction of new technologies that also become cheaper over time, ultimately pushes blue ammonia down the ranking. Tables S13 to S15 detail the underlying economics at each time point.

5 Appendix Section 3 is poorly structured and difficult to interpret. The appendix primarily presents rankings rather than absolute numerical results, particularly for the cost analysis. Under the defined system boundaries and KPIs, it would be more informative to report the absolute cost of producing 1 kg of ammonia, together with a detailed cost breakdown (e.g., CAPEX, OPEX, electricity, feedstock, CCUS-related costs) and a clear identification of the dominant cost drivers. In addition, given the inherent uncertainties, reporting a cost range rather than a single point estimate would strengthen the analysis.

Author Response: We have moved tables S12 – S15 into the Technology routes section of the supplementary information to clarify the CAPEX, OPEX, etc. and provided the 5th and 95th percentile (90% confidence interval) range of values for the two values with an uncertainty range, faradaic efficiency and current density.

6 For the LCA results, the current figures are not sufficiently clear. It is recommended to focus on the endpoint indicators, clearly explain how different impact categories are weighted and aggregated, and improve the readability of the figures. Moreover, it is unclear whether global warming potential (GWP) has been included in the final results; if not, this indicator should be explicitly reported, given its central relevance.

Author Response:

We agree that the presentation of the LCA results and the role of endpoints versus midpoints needed clearer explanation. At the same time, a different reviewer explicitly requested that we **do not rely solely on endpoints** for emerging technologies, but also present **midpoint information**. In revision we therefore adopt a **balanced approach**, making both midpoint characterisation (including GWP) and endpoint indicators explicit.

(1) Use of midpoints and endpoints, and role of GWP

We now clarify in the Methods and SI Section 2.3 that **both midpoint characterisation and endpoint indicators are used**:

- Global Warming Potential (GWP) is included as a **ReCiPe midpoint indicator** and is explicitly reported in the midpoint figures and discussed in the text when comparing grey, blue, and green routes.
- Midpoints (GWP and the other categories listed in Table S3) show the **physical emissions and resource burdens** of each technology.

In response to the other reviewer, we emphasise that these midpoint profiles are used to check that endpoint-based rankings are not misleading and to expose trade-offs between, for example, climate, toxicity, and resource use.

(2) Rationale for endpoint indicators and how aggregation works

We then explain more clearly, in SI 2.3, how and why we use endpoints:

- The **ReCiPe endpoint method** translates environmental interventions into specific **damage pathways**. For example, rather than stopping at the GWP of emissions in CO2-equivalents (a midpoint), the endpoint approach quantifies the resulting **damage to human health in Disability-Adjusted Life Years (DALYs)**, and analogous damage to ecosystems and resources.
- The conversion from midpoint to endpoint indicators is made using established **conversion factors from the ReCiPe documentation**, as implemented in SimaPro; we now cite the ReCiPe report explicitly for this step.
- Midpoint indicators are aggregated into **three endpoint damage categories**:
    - Human Health,
    - Ecosystems,
    - Resource availability.

Some midpoint categories feed into a single endpoint, while others contribute to multiple damage pathways, following the ReCiPe characterisation scheme.

- For endpoints we adopt the **Individualist (I) cultural perspective**, which focuses on **short-term, empirically supported effects**. This is particularly suitable for rapidly developing technologies, where long-term speculative effects are less certain. We now state this more explicitly in SI Section 2.4.

We rank technologies using these three endpoint damage categories because this aligns with the paper's overarching theme of **technology impacts on people, ecosystems, and resources**. While absolute emissions (e.g. GWP) are crucial, they do not fully convey the tangible consequences a technology may have on human health or ecosystem quality; endpoint indicators provide that additional perspective.

(3) Weighting and LCSA integration

We clarify that:

- Within the LCA strand, the three endpoint damage categories are each treated as **separate indicators**;
- In the Life Cycle Sustainability Assessment, these three environmental indicators are then combined with the economic and social indicators using TOPSIS, with **equal weighting across all indicators and categories** (no extra subjective weighting is added beyond the ReCiPe characterisation).

(4) Figure clarity

Finally, to address readability, we have:

- simplified and re-labelled the LCA figures so that GWP and other midpoints are clearly visible, splitting figures into two halves so all indicators are more easily visible;
- ensured consistent colour schemes and technology ordering across midpoint and endpoint plots; and
- made explicit in the captions which panels show midpoints and which show endpoints.

In summary, the revised text and figures clarify that **GWP is explicitly included**, explain how midpoint indicators are converted into endpoint damage categories using the ReCiPe method, and justify our use of endpoints for ranking, while still presenting midpoint results to satisfy the other reviewer's request and to ensure a transparent, balanced interpretation.

7 Regarding the results shown in Figure 2, it is unclear whether the assumed demand trends for the three scenarios are supported by existing literature. If demand growth is set to 0%, 50%, and 100%, the resulting differences mainly reflect changes in slope, which limits the attractiveness and interpretability of the results. Please clarify the basis for assuming linear demand growth and whether this assumption is grounded in published projections or reports.

Author Response: We thank the reviewer for this insightful comment. We agree that the basis for our demand scenarios required clearer justification and that the rationale behind the linear assumption needed to be explicitly stated. We have substantially revised the manuscript and figures to address these points.

First, regarding the basis for the demand trajectories: the 0%, 50%, and 100% growth scenarios are directly grounded in published literature reflecting ammonia's dual role as a vital fertiliser and an emerging energy carrier. These trajectories represent a spectrum of future demand, ranging from strong demand-side interventions (0%) to moderate adoption (50%) to a doubling of demand (100%) which aligns with broad industry and academic projections as discussed by Rouwenhorst and Castellanos, the IEA, Lim et al., and Saygin et al. [4–7].

Second, regarding the linear assumption and the resulting "slopes": we have clarified in the Methods that the linear trajectory is a necessary scenario simplification. Most published literature reports 2050 end-points rather than detailed intra-period time series. More importantly, we have revised the text to emphasise that the critical factor in our analysis is *not* the slope of the growth curve, but rather the absolute value of final demand. It is this absolute volume that ultimately determines whether or not net zero is achievable, as it dictates the required scale-up of alternative ammonia infrastructure and the magnitude of residual supply chain emissions.

Finally, to better interpret these results, we have clearly visualised these absolute demand trajectories and their impact on technology deployment in the revised manuscript. Figure 3 (panels a, d, g) now explicitly illustrates the 50% growth rate scenario and the resulting capacity requirements for the USA, EU, and PRC. The 0% and 100% growth scenarios, which further demonstrate how varying absolute demand impacts net-zero feasibility, are visualised in Figure S20.

We have added the following text to the main paper to illustrate these points.

"These dual roles have led to widespread projections of rapidly rising ammonia demand doubling or more by 2050[4–7]."

And

"Recognising the uncertainty in ammonia's dual role as a vital fertiliser and an emerging energy carrier, our analysis explores three demand trajectories: a doubling of global ammonia demand (100% growth) by 2050 to meet projected growth in food and energy applications; a moderate 50% growth reflecting partial energy adoption and efficiency improvements; and a flat demand (0% growth) scenario representing strong demand-side interventions and circular nitrogen management. Combining the above growth rates[4,6] (0%, 50%, 100%) with the policy scenarios (US, EU, China) result in various patterns of grey/blue/green mix evolving over time. They are visualised in in Fig 3 (Panels a, d, g) for the 50% growth, and SI 2.6 for 0% and 100% growth."

8 The direct and indirect emissions trends appear nearly identical. The authors are encouraged to better summarize and interpret the key insights from Figure 2 and to consider focusing on more meaningful or representative results rather than presenting multiple curves with similar trends. Additionally, it is not clearly stated how emissions are distributed among the gray, blue, and green pathways in this analysis. The relative shares of these pathways are expected to differ significantly across regions such as Europe, the United States, and China, yet this regional heterogeneity is not reflected or distinguished in the presented results. Further clarification or differentiation would improve the robustness of the conclusions.

Author Response: We have revised the text within this section and substantially updated the figure (now Figure 3) to display a more comprehensive range of data. The original nine curves have been consolidated into three charts to represent the three distinct locations. Furthermore, we have introduced a 50% growth scenario (with the 0% and 100% scenarios detailed in Figure S20) to better illustrate the rollout of the different ammonia production technologies—grey, green, and blue—across each location. Finally, the updated figure now includes a detailed breakdown of the residual emissions projected for 2050/2060 under each scenario, explicitly categorising them as either direct or indirect (life cycle) emissions.

9 The title and caption of Figure 3 lack essential explanations, making the figure difficult to interpret. For example, it is unclear why the United States is excluded from panels (a) and (c), and which country or region is represented in panels (d) and (e). These points should be clearly stated to avoid ambiguity.

Author Response: The Figure (now 4) caption now reads "Figure 4: Regional capacity requirements and economic performance of ammonia production pathways. Panels show 2030 and 2050/60 requirements of assumed available capacity for green hydrogen (a), CCS (b), and renewable electricity (c) in the EU, USA, and PRC. The box-and-whisker structure represents the spread of slow, medium, and fast deployment cases, linked to the uncertainty of technology deployment rates. USA has no values in panels a and c as there is no green hydrogen for the USA scenario. Panel d shows the levelised cost of ammonia (LCOA) relative to grey ammonia; panel e presents the levelised cost of GWP (CO2eq) abatement in 2030, 2050, and 2070 relative to grey ammonia. Both d & e represent location insensitive trends."

10 Panels (a–c) show the required shares of green hydrogen, CCS, and renewable energy, yet the assumptions underlying the projected capacities in 2030 and 2050 are not clearly explained. Please clarify: whether these capacity growth trajectories are based on literature, policy targets, or scenario assumptions; and how deviations from these assumed growth rates would affect the results. In particular, if the deployment of green hydrogen, CCS, or renewables falls short of expectations, emissions are likely to remain high. A discussion of this sensitivity, especially for emissions, would strengthen the analysis.

Author Response:

We agree that the basis for the capacity assumptions and their implications should be clearer, and we have expanded the Methods and Discussion accordingly. Figure 3 is now Figure 4.

(1) Basis for capacity trajectories used in Fig. 4

In the revised Methods and SI Section 1.10 we now state explicitly that:

- The **2030 and 2050/2060 capacities for CCS, green hydrogen, and renewables** are taken primarily from **published policy targets and roadmaps** for each region (EU, USA, PRC).
- Where official targets are not available or are incomplete (especially for long-term green hydrogen deployment), we supplement with **scenario ranges from international reports and recent studies** and choose central estimates that are consistent with those documents.

Thus, the capacities in Fig. 4 are not arbitrary; they represent **policy-aligned, literature-based trajectories** for "moderate" deployment in each region.

(2) Interpretation of the Fig. 4 box plots

We also clarify in the caption that in panels (a–c):

- the **horizontal axis** shows the **share** of the *assumed available* capacity that would be taken up by ammonia under our technology scenarios in 2030 and 2050/60;
- the **box-and-whisker structure** reflects the spread from the fast, medium, and slow technology-deployment cases (linked to the *k* distributions and policy ratios in Table S7), not uncertainty in the absolute regional capacity itself.

We now state this explicitly in the figure caption.

(3) Sensitivity to shortfalls in CCS / green H₂ / renewable deployment

Rather than adding new quantitative runs, we strengthen the **discussion of sensitivity**:

- We highlight in the Discussion that our capacity assumptions are **optimistic but policy-consistent**, and explicitly note that **if real-world deployment of green hydrogen, CCS, or renewables falls short of these trajectories, the shares in Fig. 4 would increase proportionally**, implying even stronger competition between ammonia and other sectors and higher residual emissions.
- We add one paragraph stating that:
    - If, for example, green hydrogen capacity reaches only ~50 % of the assumed level, then under the same ammonia technology mix:
– either ammonia would consume an even larger fraction of green hydrogen (crowding out other uses), or
– green ammonia deployment would be constrained, leading to **higher reliance on grey/blue routes and correspondingly higher life-cycle emissions**.
    - The same logic applies to CCS and renewables: shortfalls in these underpinning systems **amplify** the difficulty of reaching the emissions trajectories shown in Fig. 4.

We now make this point explicitly to underline that our results should be read as **upper-bound decarbonisation potentials conditional on achieving the assumed capacities**. Any deviation below those capacities would move actual outcomes further from net-zero.

(4) Summary of additions

Concretely, we have:

- clarified, in the Methods and SI, that the 2030/2050/2060 capacities come from **policy targets and literature roadmaps**, not purely from our own assumptions;
- improved the caption and text for Fig. 4 to explain how the box plots relate to these capacities; and
- strengthened the Discussion to emphasise that **under-deployment of CCS, green hydrogen, or renewables would raise ammonia's share of these resources and keep emissions higher than in our illustrated scenarios**.

These clarifications should make the basis and implications of Fig. 4 more transparent and highlight the sensitivity of emissions to the pace of underlying infrastructure deployment.

11 Several cost-related assumptions require clarification. It appears that the cost of grey hydrogen is assumed to remain constant, while the cost of green hydrogen is assumed to decline primarily due to falling electricity prices and electrolyzer costs. If so, it is unclear why the costs of other pathways that also rely on electrolysis do not largely decrease and are cheaper than grey scenario. Figure 3 would benefit from additional analysis and explanation to clarify these assumptions and their implications. Based on the results shown in Figure 3, it would also be helpful to explicitly summarize which pathway(s) are suggested to be the most promising options for the future.

Author Response:

We agree that the cost assumptions and their implications for Figure (now 4) should be more clearly explained, and we have clarified this in the techno-economic Methods, the SI, and the Discussion.

(1) Cost assumptions for grey, green, and electrochemical routes

In SI Section 2.1 we now state explicitly that:

- **Grey ammonia (T1)**

  – Uses **grey hydrogen from SMR** with fossil heat and grid electricity.
  – The cost of grey hydrogen is treated as **approximately constant over time**, representing a mature technology without strong cost-reduction drivers and with no explicit carbon pricing.

- – As a result, the **LCOA for grey remains essentially flat** in 2030, 2050, and 2070 (Table S13-15).

- **Green ammonia (T3)**

  – Uses **green hydrogen from water electrolysis**.
  – The green hydrogen cost declines over time due to **falling renewable electricity prices and electrolyser CAPEX**, following literature trajectories.
  – Because the Haber–Bosch section is similar to grey/blue, the main driver of LCOA reduction is the **declining cost of hydrogen and electricity**. This is why green becomes markedly cheaper relative to grey by 2050–2070 in Table S13-15 and Fig. 4d–e.

- **Other electrochemical routes (T4–T8)**

  – Also benefit from **lower electricity prices** and, where relevant, from **electrolyser cost reductions**.
  – However, their total costs are additionally governed by **current density, faradaic efficiency, cell area, and balance-of-plant complexity**.

To make this clearer, we have added Tables S12–S15, which track how the LCOA of all electrolysis-based technologies evolves over time. We explicitly note in the SI that:

"Technology routes are only categorised into Tables S12–S15 once they reach a cost level competitive with grey ammonia, through their technological advancements."

This highlights that all electrolysis technologies improve over time, but only some approach grey's cost level within the timeframe considered.

(2) Interpretation of Fig. 4 and cost-related text

In the caption and Discussion of Fig. 4 we now clarify that:

- Panels **(d–e)** show, respectively, the **relative LCOA compared with grey** and the **levelised cost of CO_2-equivalent abatement**, based on the techno-economic framework in SI Section 2.3 and the time-varying parameters in Tables S2 and S12–S16.

- The stronger cost decline seen for green ammonia directly reflects the **assumed green hydrogen cost trajectory**, whereas the more modest declines for T4–T8 reflect their more challenging performance starting points and conservative learning assumptions in the baseline scenario.

We have also added the following sentence to the **System constraints** section to make our overall conclusion explicit:

"From a cost perspective green ammonia offers the most realistic route for ammonia decarbonisation, particularly in the near term, however this route is highly dependent on the price of green hydrogen and is subject to renewable energy and green hydrogen availability restrictions."

This links the cost trends in Fig. 4d–e to the capacity requirements in Fig. 4a–c.

(3) Explicit summary of the most promising pathways

In response to your request for a clearer synthesis, the Discussion now explicitly states that:

- **Green ammonia (T3)** is the most promising decarbonisation route from a **cost perspective** initially, provided sufficient renewable electricity and green hydrogen capacity are available.
- **Blue ammonia (T2)** provides earlier emissions reduction where CCS is available but remains more expensive than grey and typically more costly per tonne of CO_2 abated than green by mid-century.
- The **emerging electrochemical routes (T4–T8)** improve over time (as shown in Tables S12–S15) but, under baseline learning, only T6 overtakes green or grey economically before 2050 by a small margin; **direct nitrogen-reduction routes (T7–T8)** only become potentially competitive in the longer term and require the disruptive performance improvements explored in Fig. 5.

These clarifications show how the cost assumptions are applied consistently across all pathways, why electrolysis-based routes do not automatically become cheaper than grey, and which options appear most promising under our scenario set.

12 In the Discussion section, the manuscript emphasizes that achieving net-zero emissions in the ammonia sector is strongly linked to the availability of green hydrogen. However, because the environmental impacts of the ammonia synthesis process itself appear to be much smaller than those associated with hydrogen production and electricity/heat supply, it remains unclear how the future role of ammonia synthesis should be interpreted. Does this imply that decarbonizing ammonia production mainly depends on selecting appropriate green hydrogen pathways and ensuring sufficient cost reductions in hydrogen supply?

Author Response: We agree that our Discussion should be clearer about the relative importance of ammonia synthesis versus hydrogen and energy supply.

(1) Relative contribution of synthesis vs hydrogen/energy supply

Our LCA and TEA results indeed show that, for **Haber–Bosch-based routes**, the **dominant contributions** to both cost and environmental impact come from:

- hydrogen production (SMR or electrolysis), and
- the electricity and heat used to produce that hydrogen and run the plant,

while the incremental burdens of the synthesis loop itself (reactor, compression, refrigeration) are comparatively smaller once a given configuration is chosen. We now state this explicitly in the Discussion, making clear that **for grey, blue, and green Haber–Bosch the hydrogen pathway and its energy source are the primary levers**.

(2) Interpretation: what "depends on green hydrogen" means

However, we do **not** intend to suggest that ammonia synthesis is irrelevant, only that:

- **For HB-based routes**, decarbonising the hydrogen and electricity/heat supply is the **first-order requirement**;
  – improvements within the synthesis loop (efficiency, heat integration, pressure optimisation) provide **second-order gains** that help reduce residual emissions and costs once low-carbon hydrogen is available.
- **For emerging electrochemical routes**, the **synthesis technology itself** (current density, faradaic efficiency, materials) is again a **first-order driver** of both cost and environmental performance, even though these routes also rely on low-carbon electricity. In these cases, better "green hydrogen" alone is not enough; the ammonia-forming electrochemical step must improve dramatically.

We now clarify in the Discussion that the statement about the centrality of green hydrogen refers specifically to **Haber–Bosch-based decarbonisation pathways**, which remain the only realistic large-scale options before 2050 in our scenarios.

(3) How we now frame the future role of ammonia synthesis

To make this interpretation explicit, we have added a short paragraph to the Discussion summarising:

- In the **near to mid-term (to 2050)**, decarbonising ammonia production is **indeed dominated by the choice and cost of low-carbon hydrogen pathways** (and their underpinning renewables/CCS), because all viable routes are variations on the Haber–Bosch theme. Selecting appropriate green or blue hydrogen options and driving hydrogen cost down is therefore the main practical lever.
- In the **longer term**, the **nature of the ammonia synthesis process itself** becomes more important if electrochemical routes (waste-to-ammonia and direct nitrogen reduction) can reach high performance. Here, advances in ammonia synthesis technology—not just hydrogen supply—could reshape the sector by enabling decentralised or less hydrogen-intensive pathways.

We now explicitly state that our results should be read as:

"In the time frame to 2050, the feasibility and cost of low-carbon ammonia are controlled primarily by the availability and cost of green (or blue) hydrogen and the supporting energy systems. Improvements in the Haber–Bosch loop are helpful but secondary. Beyond 2050, if electrochemical synthesis can achieve the disruptive performance we explore, the synthesis technology itself may become a primary determinant of both cost and environmental impact."

This clarifies that decarbonising ammonia production **mainly depends on appropriate green/blue hydrogen pathways in the near term**, but does not reduce the importance of longer-term innovation in ammonia synthesis, especially for the more radical electrochemical routes.

(4) Inclusion of hotspot analysis

To provide a clearer and more accurate picture of where the environmental impacts truly lie across the different production stages, we have included a hotspot analysis for the Life Cycle Assessment (LCA) in SI 2.3.1, Table S17. This analysis breaks down the LCA objects into their component inventory items, illustrating the exact distribution of CO_{2eq} emissions between each technology's components and clarifying the relative impact of the synthesis process itself.

13 At present, the largest barrier to green hydrogen deployment is cost. Approximately 99% of hydrogen on the market is still produced from fossil fuels, with only a small fraction coming from green or blue hydrogen. Consequently, downstream ammonia production is inevitably constrained by the availability and cost of low-carbon hydrogen. The manuscript would benefit from a clearer discussion of how the ammonia industry could be incentivized or enabled to adopt green hydrogen, given these upstream limitations.

Author Response: We agree that our Discussion should more clearly connect the upstream reality of hydrogen markets to the downstream prospects for low-carbon ammonia, and we have expanded the text accordingly.

(1) Acknowledging the current green hydrogen barrier

We now explicitly note in the Discussion that:

- Around 99 % of current hydrogen supply remains fossil-based, with only a small share from green or blue sources, and
- high production cost and limited infrastructure are the primary barriers to large-scale green hydrogen deployment.

We link this directly to our results by stating that the feasibility of green ammonia in our scenarios is conditional on overcoming these upstream constraints.

(2) How ammonia is constrained by low-carbon hydrogen availability and cost

We emphasise more clearly that:

- In our framework, ammonia is a major potential consumer of green hydrogen (Fig. 4a,c), and if green hydrogen deployment lags, ammonia production must either:
    - rely more heavily on grey/blue hydrogen, keeping emissions high, or

  - compete with other sectors (steel, power, transport) for limited green hydrogen, potentially making green ammonia uncompetitive.

- Therefore, the decarbonisation of ammonia is not only a matter of plant-level technology choice but also of system-level prioritisation of where scarce green hydrogen is used.

(3) How the ammonia industry could be incentivised / enabled

We have added a short paragraph in the Discussion outlining, at a high level, how ammonia could be encouraged to adopt green hydrogen under these constraints, consistent with our system-level perspective:

- Targeted hydrogen support for fertiliser use:
  – Long-term contracts, investment support, or carbon contracts for difference that specifically link green hydrogen supply to ammonia plants could lower effective hydrogen costs and de-risk investment in green ammonia capacity.

- Carbon pricing and standards:
  – Robust carbon pricing or performance standards for fertiliser-related emissions would narrow the cost gap between grey and green routes, making low-carbon hydrogen economically attractive for ammonia producers.

- Co-planning of hydrogen and ammonia infrastructure:
  – Integrated planning of green hydrogen hubs near major ammonia production sites can reduce transport costs and ensure that a portion of early green hydrogen capacity is earmarked for ammonia, rather than being fully absorbed by other sectors.

- Demand-side and food-system measures:
  – As our demand scenarios show, moderating ammonia demand growth through improved fertiliser efficiency and circular nitrogen management reduces the absolute volume of green hydrogen that must be secured for the sector, making uptake more realistic under constrained supply.

We frame these not as detailed policy prescriptions, but as enabling conditions that follow from our quantitative findings: because green hydrogen is the binding constraint for near-term decarbonisation of ammonia, incentives and planning must ensure that ammonia receives a viable share of early green hydrogen supply, alongside efforts to control demand.

In summary, the revised Discussion now makes explicit that:
(i) decarbonising ammonia is tightly constrained by the current cost and scarcity of green hydrogen;
(ii) our green-ammonia results are conditional on substantial green hydrogen scale-up; and
(iii) a combination of targeted support, carbon pricing/standards, infrastructure

co-planning, and demand moderation would be required to enable the ammonia industry to adopt green hydrogen at the scales implied by our scenarios.

14 If hydrogen sourcing is treated as an external constraint, it is unclear what mitigation potential remains within the ammonia synthesis stage itself. Please clarify what technological or operational improvements at the ammonia synthesis level (e.g., process efficiency, heat integration, electrification, or alternative synthesis routes) could meaningfully contribute to emissions reduction, independent of changes in hydrogen supply.

Author Response:

We agree that this distinction should be clearer. To avoid repetition, we refer to points already clarified in our previous responses and then focus on synthesising the implications.

(1) Electrochemical and alternative synthesis routes

As discussed in our responses on learning curves and disruptive steps (Fig. 5; SI 1.2, 1.11):

- For **electrochemical routes** (T4–T8), the **synthesis technology itself** (current density, faradaic efficiency, cell area, lifetime, materials) is once again a **first-order mitigation lever**, even assuming low-carbon electricity.
- Improvements in these parameters directly reduce **specific electricity use, CAPEX, and material impacts**, and for waste-to-ammonia routes they also change how polluted nitrogen streams are treated.

We now highlight in the Discussion that, beyond the 2050 timeframe where HB-based routes dominate, **innovation in the synthesis stage, particularly in electrochemical and waste-to-ammonia pathways, could provide substantial additional mitigation**, on top of electricity decarbonisation.

(2) How we now frame it

To avoid duplication, the revised Discussion cross-references the relevant sections (learning-curve response, Fig. 4/system-constraints response) and then summarises:

- **To 2050:** decarbonisation is **primarily limited by low-carbon hydrogen and energy availability**; synthesis-stage improvements offer **incremental** gains.
- **for emerging routes: synthesis-stage innovation itself** (especially electrochemical performance and waste-to-ammonia configurations) can deliver **step-changes** in cost and emissions and may reshape the sector if the disruptive trajectories we explore are realised.

This makes clear that treating hydrogen supply as a constraint does not imply that ammonia synthesis is irrelevant; it clarifies **when** and **for which routes** synthesis-level improvements are decisive.

15 In SI, several parameter assumptions and their temporal evolution require clearer justification. For example, parameters such as CD and FE are assumed to change over time, whereas lifetime is kept constant. While it is reasonable to assume that performance metrics improve with technology maturation, the manuscript does not provide sufficient evidence or references to support the assumed time-dependent trajectories. For electrolysis in particular, technology learning would also be expected to increase stack/system lifetime. In this context, the assumption of 40,000 hours in 2030 appears potentially aggressive and should be justified with credible sources and/or sensitivity ranges.

Author Response:

We agree that the temporal evolution of electrolysis parameters required clearer explanation, and we have revised the SI to address this.

(1) Clarifying what is (and is not) assumed about lifetime

We have updated the Supplementary Information to make clear that we **do not claim** a universal 40,000 h lifetime for all electrolysis technologies at 2030:

- **Tables S12–S15** have been introduced to show, for each electrolysis-based technology and time slice, the **breakdown of key parameters as they develop over time**, including current density, faradaic efficiency, energy use, CAPEX and OPEX components.
- These tables make it explicit that **lifetime is treated pragmatically within the costing framework** and is not assumed to grow aggressively beyond values consistent with current PEM electrolyser projections. No single "40,000 h by 2030" claim is made across all technologies.

We also note in the text that shortening the effective lifetime would mainly increase CAPEX-related costs for electrochemical routes and would **further disadvantage** them relative to green/blue Haber–Bosch, reinforcing rather than weakening our main conclusions.

(2) Justification of CD and FE targets and their evolution

To better justify the CD and FE trajectories, we have added explicit text to SI Section 1.2:

"Targets for faradaic efficiency are assumed to be 100% for all technologies, and current density targets for T4, 7 and 8 are selected to be 1 A cm^{-2} due to their low starting values, and T5 and 6 are targeted 3 A cm^{-2}, mirroring hydrogen electrolysis targets[20]."

This:

- clarifies that **FE targets** represent the theoretical upper bound adopted for all routes, consistent with standard electrolysis practice;
- ties **current density targets** directly to **published hydrogen electrolyser targets** (ref. 20 in the SI), rather than being arbitrary; and
- makes clear that the sigmoidal learning applied to CD and FE is intended to move technologies towards these literature-informed targets, not beyond them.

Together, the new tables and added SI text make our parameter evolution assumptions more transparent, show that we are not asserting an unrealistic lifetime step-change at 2030, and ground the CD and FE trajectories in published electrolyser targets.

16 It does not clearly account for technology learning-by-doing and economies of scale. If demand increases and deployment scales up, key cost parameters (e.g., T1, T2, T3, and other cost coefficients) would likely evolve over time rather than remain fixed. Please clarify whether such learning effects are considered; if not, discuss how excluding them may bias the cost trajectories and scenario comparisons.

Author Response: We agree that our treatment of learning needed to be stated more clearly.

(1) Sigmoidal learning does incorporate learning-by-doing and scale effects

As described in SI Section 1.2, we use a **sigmoidal learning formulation** (following Odenweller et al., Nat Energy 2022, and related work on energy technology diffusion) to govern the time evolution of key electrochemical performance parameters (current density and faradaic efficiency) and of green hydrogen costs. This type of S-curve is widely interpreted as the combined result of:

- **learning-by-doing** (cost and performance improvements as cumulative deployment grows), and
- **economies of scale** (larger plants, more standardised manufacturing, supply-chain maturation),

even though it does not explicitly separate or quantify each underlying mechanism.

We now state this explicitly in SI 1.2 and in the Methods, with an explicit reference to Odenweller et al. and the broader learning-curve literature, to make clear that our sigmoidal formulation is **intended to capture the aggregate effect** of learning-by-doing and scale-up for electrolysis and green hydrogen.

(2) Limitation: we do not disentangle individual learning drivers

At the same time, we acknowledge that:

- our implementation focuses on **aggregate performance/cost trajectories**, and
- we do **not** attempt to decompose improvements into separate contributions from plant scaling, manufacturing learning, R&D breakthroughs, or supply-chain effects.

We have added a sentence in the Discussion noting this as a limitation:

"Our sigmoidal learning formulation reflects the combined effect of learning-by-doing, economies of scale, and technology maturation for electrolysis and green hydrogen, in line with standard practice in energy-systems literature. However, it does not disentangle these individual drivers."

We also note there that this limitation **does not alter our main conclusions**: grey/blue remain relatively cheap but carbon-intensive; green's competitiveness hinges on hydrogen cost; and electrochemical routes only approach competitiveness in the longer term or under disruptive performance improvements.

17 From an LCA perspective, the SI currently lacks several essential elements. Most importantly, the system boundary and functional unit are not clearly stated. In addition, the LCIA framework (impact categories/method and characterization approach) is not sufficiently described, and inventory-level details are missing: the inputs/outputs of each unit process should be transparent to enable interpretation and reproducibility. The assumptions in Table S6 appear insufficiently supported; the authors should provide references, rationale, or uncertainty ranges for these values.

Author Response: The definition of the system boundary and functional unit have been added to the SI, and as mentioned in previous responses discussion of the LCA methodology has been strengthened.

"The LCA is performed as a cradle to gate assessment of ammonia technologies, using a functional unit of one tonne of ammonia produced."

Additionally, inventories have been improved, and a hotspot analysis has been added to improve the transparency of the LCA. This spreadsheet is available in the Figshare folder. The data in table S6 (now S7) are supported in the main text with "For example, in the United States, historical policy reliance on CCS strongly favours blue ammonia adoption and hence only blue ammonia is considered, as shown in Fig 3a[27,28]. The EU policy has a strong focus on green hydrogen to drive the economy, making green ammonia likely a preferred route[29,30]. Here, in Fig 3d, we assume 3:1 green/blue mixed ratio for new technologies deployments during the transition. In contrast, the People's Republic of China, as the world's largest

ammonia producer, adopts a more incremental approach focused on efficiency improvements and gradual CCS deployment[31,32]. Therefore, a lower green/blue ratio of 2:3 is assumed, as shown in Fig 3g."

And in the SI with

"USA and PRC are assigned 0.25 as their policies are more equivocal favouring traditional technologies and carbon capture, whereas the EU has a strong environmental and economic commitment to green hydrogen and is assigned a 0.5[32–34]. The fast, slow, and medium roll-out scenarios represent the 5th and 95th percentiles and median values for deployment when k is allowed to vary normally."

Furthermore, the HYSYS files of the production routes are provided which contain the full unit input/output for mass and energy balances. Hysys Files can be found in the Figshare folder.

Reviewer #4 (Remarks to the Author):

1) The final sentence of the first paragraph in the introduction is: "This puts us at a crucial crossroads where decisions on which ammonia route to follow are being taken." It would be beneficial to the reader to provide more clarification on ammonia route. Is this to mean "production route"? Perhaps this could be re-written as "This puts us at a crucial crossroads where decisions on the way forward for ammonia production and utilization are being taken."
Author Response: We have updated this sentence to read "This puts us at a crucial crossroads where decisions on which ammonia production route to follow are being taken"

2) The language for the "green ammonia" classification in Box 1 should be more precise. In this production scenario, Haber-Bosch is not powered by green hydrogen but rather fed with green hydrogen. Additionally, have any constraints been placed on the carbon intensity of the energy source used to power water electrolysis in the green hydrogen scenario? Perhaps the authors could consider including some additional detail about the energy source for green hydrogen production in the green ammonia scenario.
Author Response: The box has been updated to a table and green ammonia description now reads "Haber–Bosch fed with green hydrogen from low temperature water electrolysis."

3) The TEA and LCA for green ammonia production assume constant hydrogen availability (per my understanding of the Supporting Information). How is this reconciled with the variable nature of renewable energy likely to be used to power water electrolysis for green hydrogen production? Specifically, how is this variability buffered and what effect does this have on TEA and LCA results?
Author Response: For the purposes of establishing a baseline comparative analysis, our TEA and LCA assume steady-state hydrogen and renewable energy availability and do not explicitly model dynamic buffering or storage as dynamic modelling of energy buffering falls outside the scope of our steady-state TEA and LCA. If buffering were included, it would likely increase the initial capital expenditure in the TEA due to the requirement for storage infrastructure and slightly alter the LCA footprint depending on the battery chemistry used. However, as hybrid renewable and battery systems have been shown to reach a levelised cost of energy as low as $0.051/kWh [Renewable power generation costs in 2024, IRENA, 2025], which is expected to drop further by the time electrochemical ammonia scales up towards 2050, we anticipate that the techno-economic penalty of buffering this variability will be minimised in future real-world applications.

We have now:

- clarified in the Methods and SI that we assume steady-state supplies and do not explicitly simulate intra-day variability or storage; and
- added a brief note in the Discussion acknowledging this as a limitation and explaining that explicitly modelling buffering would likely slightly increase costs and impacts for green routes, but would not change our qualitative conclusion that low-cost, large-scale green hydrogen remains the critical constraint for deep decarbonisation of ammonia.

4) It would be more informative to the reader if the vertical axes titles in Figure 1b-d could be written as “LCA rank”, “LCC rank”, and “SCLA rank” respectively rather than simply “rank” for each subfigure. Also, please be consistent with the use of the terms “levelized cost” and “life cycle” cost as it pertains to the meaning of the abbreviation LCC.
Author Response: These have been added to Figure 1 as suggested, and greater attention has been given to the use of levelised cost vs life cycle.

5) Why is a longer time horizon (2060 vs. 2050) considered for the PRC’s ammonia transition relative the U.S. and the EU?
Author Response: This point has been made clearer in the main text, with this text added “PRC results include 2060 values where applicable, due to PRC policy targets in 2060 rather than 2050.”

6) Perhaps the authors could comment on what causes the emissions intensity of grey, blue, and green ammonia to be higher than the direct emissions reported in literature. What are the biggest contributors to the indirect emissions for each ammonia production pathway?

Author Response: An LCA hotspot analysis for GWP has been added to the supplementary information section 2.3.1, and this text has been added “For technologies 4-8, the energy utility driving the reaction is the highest emitter of carbon, other than T7 where green hydrogen (and the energy required therein) is highest. T3 – T8 offer substantial emissions savings over the traditionally produced T1 & T2, whose carbon emissions are due to the energy driving the high temperature ammonia production reaction and the steam methane reforming step. Notably for T2, carbon capture, despite offsetting 90% of direct emissions (assumed at 2.25 tonnes of CO_2 per tonne of ammonia) bring life cycle emissions of its own so only reduces total life cycle emissions by 1.34 tonnes (CO_2 equivalent).”

7) Is the 3:1 ratio of green/blue ammonia deployment assumed for both the U.S. and the EU? If so, please reconcile this with the previous statements that U.S. policy

favors blue ammonia production whereas EU policy favors green ammonia. If not, please better clarify these ratios for both the U.S. and EU as has been done for the PRC in the original manuscript.

Author Response: This point has been made clearer, and the following text has been added “in the United States, historical policy reliance on CCS strongly favours blue ammonia adoption and hence only blue ammonia is considered” into the Net zero transition pathways section.

Response to Reviewer 3

We sincerely thank the reviewer for their continued engagement with our manuscript. We deeply appreciate this final round of feedback, which correctly identifies the need to explicitly define the epistemological boundaries, system coupling, and ranking sensitivities of our framework.

We agree that transparency regarding these methodological limitations is essential to ensure the multi-criteria rankings are interpreted correctly. To address all three of your outstanding points, we have added a dedicated sub-section within the **Discussion** to explicitly define these boundaries, and we have removed redundant sentences from earlier in the text to ensure a cohesive narrative flow.

Please find our detailed point-by-point responses in red below.

Reviewer Comment 1: *The revised manuscript now explicitly states that the feasibility and cost of low-carbon ammonia are controlled primarily by hydrogen and energy supply systems before 2050. This raises an important question regarding what is fundamentally being compared in the present work: (i) intrinsic ammonia synthesis technologies, or (ii) broader hydrogen production system configurations coupled to ammonia production. At present, it remains difficult to distinguish the contribution of the ammonia synthesis stage itself from the contribution of upstream processes. The manuscript would therefore benefit from a clearer separation... At minimum, the authors should discuss this limitation more explicitly and clarify the extent to which the current framework should be interpreted as a “hydrogen-energy system comparison” rather than an intrinsic ammonia synthesis technology comparison.*

Author Response: We thank the reviewer for this thoughtful comment and agree that it is important to clarify what is fundamentally being compared in our framework, and how far upstream hydrogen/electricity supply can be separated from the intrinsic ammonia synthesis technologies.

We would like to clarify our hotspot analysis (Supplementary Table 17) has already shown the attributions of emissions from the synthesis (i.e., electrolyser) and the energy and utility sources. We have added a statement in the Discussion to refer Readers to this information more explicitly.

We would also like to further clarify that, for conventional grey and blue ammonia, a clean attribution of impacts between “upstream” and “synthesis” stages is not physically meaningful. Steam methane reforming, process heat generation, and the Haber–Bosch loop are co-located and tightly integrated, sharing high-temperature heat and utilities. As our hotspot analysis (Supplementary Table 17) shows, the dominant contributions to Global Warming Potential and cost arise from fuel combustion and reforming; however, these contributions are generated on the same site and within the same coupled process as the synthesis loop. In practice, hydrogen production and ammonia synthesis form a single thermodynamic unit, so

any attempt to assign a distinct fraction of the footprint to “upstream hydrogen” versus “intrinsic synthesis” would be largely arbitrary. We now state this explicitly in the Discussion and clarify that, for grey and blue routes, the framework compares **integrated production systems** rather than a cleanly separable upstream/synthesis split.

By contrast, for the fully electrified pathways the upstream energy system and the synthesis stage can be meaningfully distinguished. Electricity and green hydrogen are supplied from external systems, and the on-site synthesis loop has low emissions/impact. In these cases, almost all environmental burdens are associated with the upstream generation of electricity and hydrogen. However, the synthesis technology still matters because its efficiency determines how much upstream electricity and hydrogen are required per tonne of ammonia. Therefore, our framework is not a purely energy system comparison only. **To make this explicit, we have added a new summary table (Supplementary Table 19)** that reports the share of Global Warming Potential attributable to upstream electricity/hydrogen supply versus the on-site synthesis stage. This table illustrates that the direct contribution of the synthesis loop to emissions is small, but that differences in efficiency translate into different levels of upstream energy demand. Also the red route (high temperature) shows a hybrid feature.

We have revised the Discussion accordingly to (i) explain why impact attribution between upstream and synthesis is not possible in a rigorous way for grey and blue ammonia, (ii) highlight the clear upstream–synthesis separation that exists for fully electrified routes, and (iii) clarify that our framework compares technology–utility systems in which the synthesis step’s efficiency governs upstream energy requirements, and they together determine each route’s sustainability performance and ranking.

Reviewer Comment 2: *Although the revised manuscript now clarifies the rationale behind differentiated utility assumptions, comparability across pathways remains somewhat difficult to interpret because the environmental and economic outcomes are still strongly coupled to the assumed electricity and heat sources. Even if a full harmonized re-analysis is outside the scope of the current study, the authors should discuss more explicitly how strongly the current rankings depend on upstream utility choices and how this may affect interpretation of the comparative results. This is particularly important for interpreting the relative performance of blue, green, and red ammonia pathways.*

Author Response: We appreciate the reviewer’s request to clarify how strongly our conclusions depend on upstream utility choices. There are two layers to this dependence, which we now make explicit in the Discussion.

First, at a structural level, each ammonia technology can in principle be paired with multiple plausible electricity and heat sources. Exploring all such combinations would effectively extend the “Ammonia Rainbow” with additional colours, each representing

a distinct technology–utility system. Our current study selects a limited set of representative pairings (for example, low-temperature electrolysis with variable renewables, solid oxide electrolysis with nuclear power) to reflect likely industrial configurations. We now clarify that alternative but reasonable pairings, for instance, coupling a given electrolysis route to a more carbon-intensive grid or to a different low-carbon supply would introduce additional options into the comparison and could change the relative ordering of pathways, particularly among low-carbon routes.

Second, even within the specific pairings we adopt, the rankings are strongly influenced by the intensity of the utilities. The hotspot analysis shows that, for fully electrified routes, upstream electricity and hydrogen supply typically account for more than 95% of total Global Warming Potential and a similarly dominant share of operating costs. Consequently, varying the carbon or cost intensity of the assumed utilities by realistic margins changes total impacts by tens of percent and can shift technologies by several positions in the LCA or LCC rankings. Our existing uncertainty and sensitivity analysis of the ranking (Supplementary Figure 21) has already implied this conclusion well, but was not obviously mentioned in the main text in the previous version. We have now made it more explicit to readers about that.

In response to the reviewer, we have revised the Discussion to summarise these sensitivities and to state explicitly that our results should be interpreted as rankings of technology–utility systems. The manuscript now explains that (i) different plausible pairings would effectively expand the set of options and could alter the relative ordering, and (ii) even for the chosen pairings, the rankings remain strongly dependent on the carbon and cost intensity of the upstream utilities and refer the readers to the ranking uncertainty analysis in the SI.

Reviewer Comment 3: *The revised manuscript provides a clearer explanation of the observed rank inversions and the role of the multi-criteria framework. However, the updated discussion also suggests that several conclusions are highly sensitive to the relative ranking methodology. This raises an important methodological concern regarding the robustness and interpretability of the ranking-based conclusions, particularly for emerging technologies with highly uncertain inventories and performance assumptions... The manuscript would therefore benefit from a more explicit discussion of: (i) the dependence of conclusions on the chosen ranking framework; (ii) the potential instability of rankings under different weighting assumptions; and (iii) the distinction between absolute performance changes and relative ranking shifts.*

Author Response: This is a highly valid methodological concern. We agree that readers must be explicitly instructed to view the absolute performance data

alongside the rankings. To resolve this, we have added the following dedicated paragraph to the Discussion:

The interpretation of our comparative results also depends on how the ranking framework is constructed. The LCC, LCA and SLCA rankings are sensitive to the implicit weighting of indicators. We adopt equal weighting across all indicators as a transparent baseline, but stakeholders who prioritise, for example, climate mitigation over resource depletion, or affordability over social risk, would obtain different composite scores and a different ordering of technologies. It is therefore important to distinguish between absolute performance changes and relative ranking shifts. For instance, the absolute cost and emissions trajectories for grey and blue ammonia evolve only modestly over time, yet their positions in the strand-specific rankings decline markedly as new technologies enter the comparison set.

Editor Comments regarding formatting guidelines:

1) Abstract should be in one paragraph limit to 200 words at most;

Author Response: We have revised the Abstract to ensure it is a single paragraph and falls strictly within the 200-word limit.

2) Arrange all manuscript sections in the following order: Title, Author list, Affiliations, Abstract, Introduction(no subheading), Results (with one level subheadings), Discussion (no subheading), Methods(with one level subheadings), Data Availability, Code Availability, References, Acknowledgements, Funding, Author Contributions Statement (in one paragraph with descriptive sentences), Competing Interests, Tables, Figure Legends/Captions (for main-text figures); Note: The Data Availability and Code Availability sections must appear immediately below the Methods section;

Author Response: The manuscript has been entirely restructured to follow this exact sequence. All subheadings have been removed from the Introduction and Discussion sections, and subheadings in the Results and Methods sections have been restricted to a single level. The Data Availability statements now appear immediately after the Methods section.

3) The section of methods should be clear enough that the whole study could be reproduced;

Author Response: We have carefully reviewed our Methods section against this requirement and can confirm it meets this standard. As established during the previous round of peer review, the methodology is comprehensively detailed to ensure full reproducibility. All governing equations, probabilistic learning parameters, utility and system boundary assumptions, and complete mass and energy balances

are explicitly provided within the main Methods text, the accompanying Supplementary Information, and the source data files.

4) Please use sentence to describe the authors contributions, for example, “Z.Z. and X.X. designed the study. M.C., C.G., L.Z. carried out the modeling and analyses; ”

Author Response: The Author Contributions Statement has been rewritten into a single paragraph using descriptive sentences and author initials, in accordance with the provided sample.

5) The code should be uploaded to online repositories and share the link in the code availability statement,

Author Response: We clarify that no custom computer code or algorithm is used to generate results that are reported in the paper and central to its main claims. The only code we use is to visualise data in some figures but the data itself is generated by commercial software. To avoid confusion, we have deleted the Code Availability Statement.

6) Supplementary items are labelled and contain only these elements: Supplementary Figure 1 / Supplementary Table 1 / Supplementary Note 1 / Supplementary Discussion / Supplementary Methods / Supplementary References.

Author Response: All supplementary items have been renamed and reformatted to strictly follow this labelling convention.
